# Advancing nutrition measurement: Developing quantitative measures of nutrition service quality for pregnant women and children in low‐ and middle‐income country health systems

**DOI:** 10.1111/mcn.13279

**Published:** 2021-11-03

**Authors:** Shannon E. King, Ashley Sheffel, Rebecca Heidkamp, Yvonne Yiru Xu, Shelley Walton, Melinda K. Munos

**Affiliations:** ^1^ Department of International Health Johns Hopkins Bloomberg School of Public Health Baltimore Maryland USA

**Keywords:** child public health, indicator development, malnutrition, maternal public health, nutritional interventions, quantitative methods

## Abstract

The health sector plays an important role in the delivery of high‐quality nutrition interventions to women and children in low‐ and middle‐income countries (LMICs). However, there are no standardized approaches to defining and measuring nutrition service quality in these contexts. This study aims to systematically develop quality of care indices for direct health systems nutrition interventions using a five‐step process: (1) identify recommended interventions for inclusion in indices, (2) extract service readiness, provision of care, and experience of care items from intervention‐specific clinical guidelines, (3) map items to data available in global health facility surveys, (4) conduct an expert survey to prioritize interventions and items, and (5) use findings from previous steps to propose quality of care metrics. Thirty‐two recommended interventions were identified, for which the guidelines review yielded 763 unique items that were reviewed by experts. The proposed nutrition quality of care indices for pregnant women reflects eight interventions and the indices for children under 5 reflects six interventions. The indices provide a standardized measure for nutrition intervention quality and can be operationalized using existing health facility assessment data, facilitating their use by LMIC decision makers for planning and resource allocation.

**Key Messages**

- Achieving meaningful reductions in the burden of maternal and child malnutrition across low‐ and middle‐income countries requires both increasing the coverage of heath sector nutrition interventions and ensuring high‐quality delivery of these services. However, many countries lack information on nutrition intervention quality.
- Standardized indices for nutrition quality of care can help facilitate consistent measurement of nutrition service quality and support global monitoring and country planning processes.
- This study proposes a set of nutrition quality of care indices for pregnant women and children, which is an important step towards improving availability of data on nutrition intervention quality that will enable LMIC governments to prioritize and allocate resources towards more effective services.
- Revision of existing health facility assessments to better support quality of care measurement for nutrition interventions as well as further refinement of global implementation guidance for nutrition interventions to facilitate translation of research to practice would bolster efforts to improve quality of nutrition services delivered through the health system in LMICs.

## 1 INTRODUCTION

Maternal and child malnutrition remains a prominent issue in low‐ and middle‐income countries (LMICs) where there remains a high prevalence of undernutrition and a growing prevalence of overweight and obesity (Food and Agriculture Organization of the United Nations, 2019; Food Security Information Network, 2019; Ng et al., 2014; Victora et al., 2021). Addressing this multifaceted problem is challenging as it requires a multisectoral approach to address the underlying causes (Bhutta et al., 2008; Ruel et al., 2013). Within a multisectoral approach to reducing malnutrition across LMICs, the health sector plays an important role in delivering direct interventions through health facilities (Heidkamp et al., 2021; World Health Organization [WHO], 2019). Evidence‐based nutrition interventions delivered by the health sector have the potential to accelerate progress towards global nutrition targets if they are scaled at high quality (Bhutta et al., 2013; Kruk et al., 2018). However, there is limited data on nutrition intervention coverage and even less data specific to the quality of nutrition service delivery (Amouzou et al., 2019; Bhutta et al., 2020; Buckland et al., 2020; International Food Policy Research Institute, 2014).

As the maternal, newborn, and child health community recognizes the importance of quality of care (QoC) to reaching population health goals, there is a shifting focus on improving and therefore measuring and monitoring service quality (Amouzou et al., 2019; WHO, n.d.‐a, n.d.‐b). While, assessing and improving QoC has been a growing priority for the maternal, newborn and child health community, it has yet to gain momentum within the nutrition community (Mallick et al., 2019; Moxon et al., 2018; Sheffel et al., 2019; Tripathi et al., 2015). The first step in measuring nutrition QoC is to more specifically define QoC for nutrition services and to develop clear and measurable indicators to operationalize the definition.

Challenges exist to defining nutrition QoC, making this a complex undertaking. Integration of nutrition interventions into the delivery of other health services such as antenatal care (ANC) and sick and well child visits has facilitated delivery of nutrition interventions. However, this integration presents challenges to clearly delineating nutrition services from other interventions and in turn to developing nutrition QoC indicators. Nutrition interventions are typically integrated across multiple broader service area guidelines (e.g., ANC and integrated management of childhood illness [IMCI]) and not necessarily distinguished as nutrition‐focused (Gillespie et al., 2019). There is also a lack of specific guidelines for how to operationalize nutrition interventions at country or global level, particularly those involving behaviour change counselling.

Despite these challenges, monitoring of nutrition QoC is critical to ensuring delivery of high‐quality nutrition services to women and children. Establishing standardized measures of nutrition QoC will enable consistent measurement of nutrition service quality and support both global monitoring and country planning processes. Establishing measures of service quality which utilize data from commonly implemented health facility assessments in LMICs, such as the Service Provision Assessment (SPA) and Service Availability and Readiness Assessment (SARA), will support feasibility of country operationalization (ICF, n.d.; Sheffel et al., 2018; WHO, n.d.‐a, n.d.‐b). This study aims to systematically develop QoC measures for prioritized nutrition services delivered to pregnant women and children in LMICs that are feasible to measure using extant data in LMICs.

## 2 METHODS

### 2.1 Overview

To define QoC measures for direct health systems nutrition interventions, we followed a five‐step process: (1) identify globally recommended interventions for pregnant women and children in LMICs delivered through the health sector for inclusion in indices; (2) extract service readiness, provision of care, and experience of care items from intervention‐specific clinical and service implementation guidelines; (3) map the identified items from the guidance documents to available data in global health facility surveys; (4) conduct an expert survey to prioritize interventions and items; and (5) develop QoC metrics informed by QoC frameworks, clinical guidelines, and expert survey results.

### 2.2 Identification of interventions

We conducted a structured literature review (search terms in the Supporting Information) and a review of guidance documents identified through the WHO Library of Evidence for Nutrition Actions to develop a comprehensive list of nutrition interventions in LMICs (Figure S1/Table S1). Interventions were selected for the next step of guideline review and extraction if they met five criteria: (1) nutrition‐direct interventions (Keats et al., 2021), (2) targeting women during pregnancy and children under five, (3) globally recommended for LMICs based on evidence of impact on nutrition status, (4) delivered through the health sector, and (5) facility‐based service delivery.

### 2.3 Guideline extraction

The aim of the guideline extraction step was to identify discrete elements or ‘items’ recommended for intervention delivery (Table S2). For each intervention that met the previously mentioned criteria, we first reviewed WHO facility‐level service delivery guidelines; where those were lacking, we identified and reviewed guidelines from other credible global institutions (e.g., USAID, the Hellen Keller Foundation, and UNICEF), country‐specific guidelines, and/or published peer‐reviewed literature.

Using the WHO Quality of Care Framework for Maternal and Newborn Health, we classified items into the following dimensions: (1) provision of care, (2) experience of care, and (3) service readiness (Tunçalp et al., 2015; WHO, 2016). Provision of care refers to the quality of delivery of interventions by providers to clients (i.e., the content of care), which includes following evidence‐based practices for routine care and management of complications. In many assessments, including the SPA, provision of care is assessed by observing consultations and recording what occurs. The provision of care dimension was further categorized into domains based on the Nutrition Care Process Model, an internationally supported standardized workflow model used by nutrition and dietetic professionals (Swan et al., 2017). Experience of care refers to the client's experience including effective communication by the care provider about the services provided, client expectations, and client rights; care provided with respect and preservation of dignity; and client access to emotional and social support of their choice. Service readiness refers to the capability of health facilities to provide a service of minimum acceptable standards and is measured by the availability of both physical resources and human resources. Service readiness was further categorized into six domains: basic amenities, equipment, medicines and commodities, diagnostics, guidelines, and staff training.

### 2.4 Mapping items to data sources

Each item identified during the guideline extraction process was matched with available items from the SPA and SARA standard questionnaires. The SPA and SARA are facility‐based surveys implemented in LMICs that collect data on health service delivery (ICF, n.d.; WHO, n.d.‐a, n.d.‐b). While these data sources capture indicators relevant to nutrition, they do not present globally agreed‐upon indicators to capture all nutrition interventions provided through the health system. However, the SPA and SARA surveys are the most widely implemented health facility surveys in LMICs and provide nationally representative data on QoC (Sheffel et al., 2018). Utilizing these surveys in the design of the nutrition QoC measures provides a means to operationalize the quality scores using extant data in LMICs.

The level of agreement between the item in the guideline and item in the SPA and SARA was classified as an exact match, partial match, or nonmatch. Exact matches were items from the guidance documents for which an exact indicator was available within the SPA and SARA questionnaires (e.g., haemoglobin testing or providing instruction on how to take IFA). Nonmatches were items for which there was no appropriate match within the SPA and SARA questionnaires (e.g., balanced energy proteins supplements). Partial matches were subsequently divided into high and low partial matches based on the specificity of the SPA and SARA item compared to the guidance document. For example, specific intervention guidelines (e.g., IFA supplementation) were considered a high partial match to broader service areas guidelines that include that intervention (e.g., ANC guidelines). Staff trained in a specific nutrition intervention (e.g., IFA supplementation) were considered a low partial match to SPA and SARA indicator of staff trained in a broader service area package (e.g., ANC training) because it is not clear whether the specific intervention was included in training.

### 2.5 Expert survey design

The criteria for participation in the expert survey was self‐reported (1) knowledge of nutrition interventions delivered through the health system in LMICs, (2) clinical knowledge of protocols, and physical and human resources requirements for high‐quality delivery of nutrition interventions through LMIC health systems, and (3) expertise in nutrition for children or pregnant women. The recruitment of survey participants began by reaching out to known nutrition experts through a global nutrition network to identify a pool of qualified respondents. This process resulted in the identification of 92 nutrition experts meeting the criteria that were sent the expert survey.

The survey design was informed by prior research with similar aims of developing QoC metrics (Sheffel et al., 2019). The survey was divided into three sections: (1) respondent demographic information, (2) nutrition interventions during pregnancy, and (3) nutrition interventions for children under 5. Respondents self‐selected whether to respond to one or both intervention sections based on their area(s) of expertise. Nutrition interventions provided during delivery or in the immediate postnatal period (e.g., kangaroo mother care) were not included because SPA/SARA do not have data on the provision of care or experience of care for these interventions.

Table 1 lists all survey items associated with each target group (pregnancy and childhood). Items were consolidated into three main dimensions and a total of 10 domains: (1) service readiness (basic amenities, equipment, medicines and commodities, diagnostics, guidelines, and staff training), (2) provision of care (assessment, intervention, and documentation), and (3) experience of care. All matched items (exact, high partial, or low partial) were included in the expert survey. Unmatched items were excluded from the expert survey with a few notable exceptions. Tracer commodities for nutrition interventions for which the key commodity was not available in the SPA or SARA data were included in the expert survey (e.g., calcium and multiple micronutrient supplements). A list of basic amenities was included based on existing SARA indicators (WHO, 2015). Very few interventions had guidelines that addressed aspects of experience of care; those that did varied significantly in terms of items and level of detail. Therefore, for the experience of care items, we used the SPA Exit Interview Questionnaire regarding satisfaction of service interactions in the overall visit (i.e., not specific to the nutrition interventions). A complete version of the expert survey can be found in the Supporting Information.

**Table 1:** Expert survey scores for items of nutrition interventions delivered to pregnant women and children under 5

Dimension/domain	Item	Mean Likert	N for ranking	Mean ranking	Sutrop index
**Pregnant women**					
Service readiness					
Basic amenities	Improved water source	3.67	18	13.61	0.0575
	Place for women to sit/lie down	3.58	14	13	0.0468
	Emergency transportation	3.38	11	11.18	0.0428
	Sanitation facilities	3.5	14	14.93	0.0408
	Power	3.25	10	10.7	0.0406
	Clean environment	3.62	15	17.47	0.0373
	Room with auditory and visual privacy	3.21	8	14.12	0.0246
	Communication equipment	2.67	2	22.5	0.0039
	Computer with email/internet	2.42	1	32	0.0014
Equipment	Adult weighing scale	3.65	16	6.31	0.1102
	MUAC tape	3.52	13	7	0.0807
	Handwashing soap	3.54	13	9.08	0.0623
	Disposable gloves	3.33	11	8.36	0.0572
	Stadiometer or height rod	3.29	13	10.15	0.0557
	Auto‐disable syringes with needles; single use standard disposable syringes with needles	3.35	13	11.15	0.0507
	Disinfectant (environmental)	2.87	5	5.8	0.0375
	Visual aids for education	3.17	8	10.88	0.0320
	Alcohol‐based hand rub	3.04	5	8.4	0.0259
	Hot air oven/boiling mechanism/autoclave	2.91	7	13	0.0234
	Sharps container	2.96	6	14.5	0.0180
	Waste receptacle (pedal bin) with lid and plastic bin liner	2.83	5	20.2	0.0108
	Other, non‐hazardous waste receptacle	2.33	1	20	0.0022
Medicines and commodities	Folic acid tablet (either stand‐alone or in combination with iron)	3.88	20	8.95	0.0972
	Iron tablets (stand‐alone tablets)	3.83	19	9.21	0.0897
	Sulfadoxine‐pyrimethamine (SP) for IPTp	3.29	12	10.5	0.0497
	Albendazole/Mebendazole	3.58	14	12.29	0.0495
	Calcium supplements	3.12	10	10.5	0.0414
	Balanced energy and protein supplements	2.61	3	6.33	0.0206
	MMS formulation: UNIMAPP formula	2.59	5	12	0.0181
	Vitamin A capsules	2.96	6	16.83	0.0155
Diagnostics	Capability to test haemoglobin levels	3.88	21	10.95	0.0834
	Capability to test blood glucose levels	3.58	14	13.43	0.0453
Guidelines	Guidelines for antenatal care (ANC)	3.88	22	12.91	0.0741
	Guidelines for infant and young child feeding (IYCF)	3.58	16	16.31	0.0426
	Guidelines for intermittent preventive treatment of malaria during pregnancy	3.58	17	17.53	0.0422
Staff trained	Staff trained in counselling for ANC (e.g., nutrition, FP, and newborn care)	3.67	17	8	0.0924
	Staff trained in ANC screening (e.g., blood pressure, urine glucose, and protein)	3.67	17	10.59	0.0698
	Staff trained in antenatal care (broad ANC training)	3.58	15	10.53	0.0619
	Staff trained in complications of pregnancy and their management	3.67	17	12	0.0616
	Staff trained in nutritional assessment of the pregnant woman, such as BMI calculation and MUAC measurement	3.33	13	11.08	0.0510
	Staff trained in infant and young child feeding	3.58	15	13.27	0.0492
	Staff trained in intermittent preventive treatment of malaria in pregnancy	3.29	10	17.8	0.0244
	Staff trained in standard precautions for safe blood collection	3.04	4	20.25	0.0086
Provision of care					
Assessment	Asked about, performed, or referred the client for haemoglobin testing	3.73	16	2.94	0.2593
	Asked about when the clients last menstrual period began	3.55	14	2.79	0.2393
	Inspected conjunctiva or examined the client for pallor	3.36	11	3.64	0.1441
Intervention	Provided or prescribed iron pills or folic acid pills or both			4.47	0.2022
	Discussed nutrition (i.e., quantity or quality of food to eat) during the pregnancy	3.86	19	4.07	0.1756
	Explained the purpose of iron or folic acid	3.68	15	4.76	0.1699
	Explained how to take iron or folic acid pills	3.73	17	6.36	0.1049
	Advised on potential side effects of IFA	3.64	14	5.82	0.0900
	Discussed early initiation and prolonged breastfeeding	3.36	11	6.73	0.0779
	Provided or prescribed preventive treatment: IPTp‐SP	3.45	11	6.29	0.0530
	Provided albendazole or mebendazole	3.19	7	8.67	0.0494
	Discussed exclusive breastfeeding	3.36	9	8.67	0.0494
	Explained the purpose of deworming	3.18	9	9.5	0.0401
	Explained the purpose of preventative treatment with anti‐malaria medicines	3.27	8	9.43	0.0354
	Explained potential side effects of IPTp‐SP	3.23	7	10	0.0095
Documentation	Documented IFA supplement provision	3.64	14	8	0.0833
	Documented iron supplement provision/prescription	3.64	14	9	0.0741
	Documented provision of deworming medication	3.45	10	10.5	0.0454
	Documented IPTp‐SP provision	3.24	8	9.5	0.0401
	Documented multiple micronutrient supplement provision	3.00	5	7.6	0.0313
	Documented vitamin A supplement provision	2.86	6	10.17	0.0281
	Documented calcium supplement provision	2.90	4	9.5	0.0201
	Documented balanced energy protein supplement provision	2.57	0	NA	NA
Experience of care					
	Client is able to discuss problems or concerns about pregnancy with provider	3.86	16	1.38	0.6465
	Client satisfied with how the staff treated them	3.67	13	2.62	0.2762
	Client satisfied with the amount of explanation received about the problem or treatment	3.76	13	3.08	0.2347
	Client has privacy from having others hear the consultation	3.38	8	3.38	0.1317
	Client has privacy from having others see the consultation	3.33	7	3.14	0.1237
	Client satisfied with the cost for services or treatments	3.10	5	3.6	0.0772
	Client satisfied with the availability of medicines at the facility	3.19	6	4.33	0.0769
	Client satisfied with the cleanliness of the facility	3.05	5	4.6	0.0604
	Client satisfied with the wait time	2.81	3	4.33	0.0385
	Client satisfied with the hours of service at the facility	2.67	3	5	0.0333
	Client satisfied with the number of days services are available at the facility	2.48	0	NA	NA
**Children under 5**					
Service readiness					
Basic amenities	Improved water source	3.78	5	15.33	0.0510
	Clean environment	3.70	5	19.53	0.0378
	Sanitation facilities	3.65	8	19.20	0.0340
	Power	3.13	12	18.00	0.0169
	Emergency transportation	3.17	11	20.62	0.0169
	Room with auditory and visual privacy	3.04	10	25.00	0.0122
	Communication equipment	2.91	13	28.50	0.0061
	Computer with email/internet	2.26	5	NA	NA
Equipment	Infant weighing scale (100‐gram graduation)	3.87	20	6.75	0.1288
	Height or length board	3.83	20	6.85	0.1269
	Growth charts	3.78	19	7.11	0.1163
	Tape for measuring circumference	3.61	15	7.47	0.0873
	Handwashing soap	3.74	18	10.22	0.0766
	Regular thermometer	3.57	16	14.12	0.0492
	Blank/unused individual child vaccination cards or booklets	3.48	12	12.00	0.0435
	Visual aids for teaching care givers	3.30	10	13.60	0.0320
	Disposable gloves	3.17	8	13.38	0.0260
	Auto‐disable syringes with needles or single use standard disposable syringes with needles	3.04	8	15.25	0.0228
	Alcohol‐based hand rub	3.00	6	11.83	0.0220
	Disinfectant (environmental)	2.83	4	8.00	0.0217
	Cup and spoon	2.52	4	11.75	0.0148
	Nasogastric tube	2.67	5	17.20	0.0126
	Intravenous infusion kit	2.91	5	18.20	0.0119
	Sharps container	2.82	5	19.20	0.0113
	Hot air oven/boiling mechanism/autoclave	2.41	3	14.33	0.0091
	Waste receptacle (pedal bin) with lid and plastic bin liner	2.82	3	18.33	0.0071
	Other, non‐hazardous waste receptacle	2.41	2	23.50	0.0037
Medicines and commodities	Oral rehydration salts (ORS) sachets	3.74	18	13.00	0.0602
	Vitamin A capsules	3.65	17	16.00	0.0462
	Zinc tablets	3.65	15	14.13	0.0461
	Ready‐to‐use therapeutic foods	3.48	14	14.00	0.0435
	F‐75 formula	3.23	12	15.42	0.0338
	Albendazole/Mebendazole	3.43	12	16.33	0.0319
	Multiple micronutrient powders	3.09	8	12.00	0.0290
	Artemisinin‐based combination therapy	2.95	8	16.62	0.0209
	Multiple micronutrient supplements in tablet form for children	2.74	7	14.71	0.0207
	Iron tablets	2.70	5	13.80	0.0158
	Ampicillin/gentamycin injection	2.95	7	19.71	0.0154
	Insecticide treated bednets (LLITN)	2.82	3	14.67	0.0089
	Folic acid tablet (stand‐alone only)	2.43	3	14.67	0.0089
	Normal saline (5% dextrose)	2.95	4	20.50	0.0085
	Small quantity‐lipid nutrition supplement (SQ‐LNS)	2.50	3	15.67	0.0083
	Ringers lactate	2.88	4	24.25	0.0072
Diagnostics	Capability to test haemoglobin levels	3.74	17	19.06	0.0388
	Capability to test for malaria (RDT or microscopy)	3.35	10	17.20	0.0253
	Capability to test blood glucose levels	3.14	9	23.89	0.0164
	Capability to test blood chemistry (serum creatinine and liver function tests)	2.68	3	19.00	0.0069
Guidelines	Guidelines for growth monitoring in children	3.96	22	18.09	0.0529
	Guidelines for infant and young child feeding counselling	3.96	22	18.09	0.0529
	Guidelines for integrated Management of Childhood Illness (IMCI)	3.78	18	20.28	0.0386
	Guidelines for malaria prevention, testing, and diagnosis	3.30	10	23.30	0.0187
Staff trained	Staff trained in breastfeeding	3.96	22	9.86	0.0970
	Staff trained in complementary feeding in children	3.91	21	12.29	0.0743
	Staff trained in integrated Management of Childhood Illness	3.78	18	13.72	0.0570
	Staff trained in micronutrient deficiencies and/or nutritional assessment	3.52	14	11.14	0.0546
	Staff trained in diagnosis and/or treatment of diarrhoea	3.61	14	19.79	0.0308
	Staff trained in nutrition counselling for newborn of mother with HIV/AIDS	3.57	13	19.77	0.0286
	Staff trained in case management/treatment of malaria in children	3.22	8	18.88	0.0184
	Staff trained in malaria diagnosis using RDT or microscopy	2.83	4	13.25	0.0131
	Staff trained in malaria treatment and dosing	2.96	4	19.25	0.0090
Provision of care					
Assessment	Asked about feeding or breastfeeding habits or practices for child during illness	3.65	16	3.94	0.1767
	Asked about normal breastfeeding feeding habits or practices when the child is not ill	3.52	14	3.71	0.1639
	Weighed the child	3.87	20	5.80	0.1499
	Asked about normal feeding habits or practices when the child is not ill	3.48	13	4.77	0.1185
	Plotted child weight on growth curve	3.73	16	8.19	0.0850
	Pressed both feet to check for edema	3.65	16	8.62	0.0807
	Asked if the child is unable to drink or breastfeed	3.48	13	9.08	0.0623
	Took child's temperature by thermometer	3.48	14	10.36	0.0588
	Asked if the child has diarrhoea	3.52	14	11.14	0.0546
	Counted respiration (breaths) for 60 s	3.41	12	11.00	0.0474
	Assessed previous vitamin A supplementation history	3.09	7	7.14	0.0426
	Asked if the child has vomited	3.26	11	12.09	0.0396
	Assessed palm pallor	3.14	10	11.60	0.0375
	Auscultated child (listen to chest with stethoscope) or count pulse	3.27	11	12.91	0.0370
	Asked if the child has convulsions	3.14	10	12.00	0.0362
	Checked skin turgor for dehydration (e.g., pinch abdominal skin)	3.23	10	12.40	0.0351
	Asked about presence of cough, or difficulties breathing (e.g., fast breathing or chest‐in drawing)	3.30	10	13.70	0.0317
	Asked if the child has a fever	3.04	8	11.00	0.0316
	Asked if the child vomits everything	3.09	9	13.00	0.0301
	Looked at the child's immunization card or asked caretaker about child vaccination history	3.39	10	15.10	0.0288
	Felt the child for fever or body hotness	3.00	7	11.57	0.0263
	Provided malaria testing results	3.26	10	17.10	0.0254
	Looked into child's mouth	3.00	9	16.11	0.0243
	Offered the child something to drink or asked the mother to put the child to the breast	2.96	8	15.38	0.0226
	Looked in child's ear	2.86	9	17.44	0.0224
	Felt behind child's ear	2.77	8	18.88	0.0184
	Checked for enlarged lymph nodes in 2 or more of the following sites: Neck, axillae, groin	2.90	6	20.33	0.0128
	Assessed previous deworming history	2.78	4	14.50	0.0120
	Checked for neck stiffness	2.73	5	18.60	0.0117
Intervention	Mentioned the child's weight or growth to the caretaker, or discussed growth chart	3.74	19	12.21	0.0677
	Provided general information about feeding or breastfeeding the child even when not sick	3.74	17	12.29	0.0601
	Told the caretaker to continue feeding the child during this illness	3.70	16	18.00	0.0386
	Discussed follow‐up visit for the sick child	3.65	17	22.47	0.0329
	Provided education on home treatment and preparation of ORS	3.52	13	19.85	0.0285
	Prescribed zinc	3.48	13	19.92	0.0284
	Prescribed or provided initial ORT in facility	3.30	12	19.67	0.0265
	Provided/prescribed vitamin A	3.43	13	21.77	0.0260
	Provided ACT for malaria treatment if child diagnosed with malaria	3.52	11	21.55	0.0222
	Referred the child to appropriate level of care	3.43	13	25.46	0.0222
	Advised and encouraged adherence on continued use of zinc	3.43	11	22.55	0.0212
	Prescribed or provided RUTF or supplementary foods	3.04	8	18.75	0.0186
	Prescribed or provided extra feeding liquids	2.95	8	20.88	0.0167
	Prescribed home ORT	3.13	8	22.88	0.0152
	Administered deworming medication	3.09	6	31.50	0.0083
	Prescribed or provided multiple micronutrient powders	2.61	3	19.00	0.0069
	Provided instruction on correct dosing of iron supplementation in children	2.70	5	31.40	0.0069
	Provided/prescribed iron supplement	2.70	5	32.00	0.0068
	Prescribed or provided multiple micronutrient supplements	2.45	1	22.00	0.0020
	Prescribed or provided SQ‐LNS	2.05	0	NA	NA
	Provided instructions on correct supplement dosing and use of SQ‐LNS	1.95	0	NA	NA
Documentation	Documented vitamin A supplement provision	3.74	17	22.71	0.0326
	Documented multiple micronutrient powder provision	3.13	11	20.91	0.0229
	Documented deworming medication provision	3.39	12	23.17	0.0225
	Documented iron supplement provision	3.39	11	25.64	0.0187
	Documented multiple micronutrient supplement provision	3.00	6	24.67	0.0106
	Documented SQ‐LNS provision	2.65	2	15.50	0.0056
Experience of care					
	Client is able to discuss problems or concerns about child's illness with provider	3.96	20	1.85	0.5148
	Client satisfied with the amount of explanation received about the problem or treatment	3.74	17	2.53	0.3201
	Client satisfied with how the staff treated them	3.57	12	1.83	0.3117
	Client satisfied with the availability of medicines at the facility	3.26	11	4.45	0.1221
	Client satisfied with the cost for services or treatments	3.18	9	4.00	0.1129
	Client has privacy from having others hear the consultation	3.26	9	4.78	0.0871
	Client has privacy from having others see the consultation	3.17	6	4.17	0.0686
	Client satisfied with the cleanliness of the facility	3.13	7	6.00	0.0614
	Client satisfied with the wait time	2.65	3	4.00	0.0357
	Client satisfied with the number of days services are available at the facility	2.70	4	5.75	0.0331
	Client satisfied with the hours of service at the facility	2.65	3	5.67	0.0212

Under each domain, respondents were first asked to categorize each item using a 5‐point Likert scale based on its relative importance in the delivery of a high‐quality package of nutrition services (*unimportant*, *somewhat important*, *very important*, *essential*, or *don't know*). Next, respondents were presented with all the items they categorized as “essential” within a dimension and asked to rank order these items according to their relative importance for inclusion in a summary measure of nutrition service quality. Finally, an open‐ended question asked respondents to identify interventions and/or items they thought were missing from the survey. The survey was administered using Qualtrics software (Qualtrics, 2020).

### 2.6 Data analysis

Survey responses were analysed using STATA 14 (StataCorp, 2015). Answers from partially completed surveys were included for each completed dimension. First, descriptive statistics including response frequencies, means, and medians of Likert scores as well as means and medians of item rankings were generated. Don't know responses were excluded from the analysis. Subsequently, the Sutrop score, a measure of salience that takes into account both frequency and position of rank that ranges from 0 (low salience; low importance in both the frequency and position of rank) to 1 (high salience; high importance in both the frequency and position of rank), was calculated using FLARESv1.0 (Wencelius et al., 2017). The Sutrop score accounted for each respondent having a different number and subset of items to rank given their Likert score responses.

### 2.7 Development QoC indices

We aimed to develop QoC indices that reflected sets of priority nutrition interventions for the two subpopulations (pregnant women, children under 5) and the essential items required to deliver those interventions. Prioritization of items was important to ensure the QoC indices focused on nutrition interventions specifically. We also aimed to limit indices to a reasonable number of items so they were feasible to collect and calculate and in turn more likely to be used.

Within each of the dimensions (service readiness, provision of care, and experience of care), items that fell in the bottom tertile of the range of Sutrop scores were excluded. We arrived at this exclusion cut off by comparing several thresholds. As a sensitivity analysis, we examined other statistics including the mean Likert score, the frequency of essential responses within the Likert score, a product of the rank score and the number deeming the item essential, and the Smith index, an alternative salience measure to the Sutrop score (Smith & Borgatti, 1997), and found the set of included items to be quite similar across all approaches.

We identified several inconsistencies in items between the readiness and provision of care dimensions by intervention (e.g., the provision of the commodity was included but the availability of the commodity was excluded) and included both items in the QoC indices to ensure a consistent measure. For example, based on expert survey results alone, oral rehydration salts (ORS) was included in the readiness dimension, but the provision of oral rehydration therapy (ORT) was excluded in the provision of care dimension. This was resolved by adding ORT provision back to the provision of care dimension. Finally, we examined the remaining items to identify interventions that were completely excluded from the QoC indices. Interventions excluded based on survey cut‐off criteria but identified by the research team as important for maternal or child nutritional status were added back into the index if health facility assessment data for related items was available.

Finally, we assessed possible methods for combining the QoC index items into a score for readiness and a score for provision and experience of care for pregnant women and children under 5, respectively. We considered the distribution of items within dimensions to decide on a simple or weighted average approach to calculating the index scores. If the number of items was similar in each dimension, then a simple average would be used, if not then a dimension weighted average would be used.

### 2.8 Ethical consideration

The institutional review board at the Johns Hopkins Bloomberg School of Public health deemed this non‐human subjects research.

## 3 RESULTS

### 3.1 Identification of interventions

A total of 71 interventions were identified through the literature and document review and 28 interventions met the inclusion criteria for guideline review and extraction (Table S1). Many of the excluded interventions were related to overnutrition and do not have global recommendations. We included four interventions that did not adhere to all inclusion criteria but are categorized as nutrition services in LMICs: growth monitoring and promotion programs, deworming in children, ORS treatment for diarrhoea, and zinc treatment of diarrhoea (Gillespie et al., 2019; WHO, 2018a). (Figure S1).

### 3.2 Guideline extraction and mapping items to data sources

Table S2 includes the list of items by dimension identified through the review of guidelines for the 32 interventions. Most guidance documents provided specific information on screening assessments and the age specific dosing for interventions when required. However, guidance documents for behaviour change counselling and education interventions lacked specificity about key messages and effective communication strategies.

Table S3 presents the mapping of items from guidelines to the items available within the SPA and SARA questionnaires. There were 204 items that had exact matches available in the SPA and SARA, 123 items with high‐partial matches, 124 items with low‐partial matches, and 314 items with no matches. The overall list of items was generated by assessing items required per nutrition intervention. Some nutrition interventions require the same items. As such the item count has overlap and therefore is not a count of unique items. Many of the exact matches were service readiness items required to provide health services generally, but not specific to nutrition (e.g., scales, syringes, sharps containers, equipment to conduct biospecimen samples). Similarly, many exact matches for provision of care items pertained to the initial assessment of the patient and are not unique to nutrition.

Partial and nonmatches reflected a number of common limitations in the SPA and SARA specific to nutrition services. There was a lack of items on specific nutrition training topics and a lack of specificity about guideline content for nutrition services. No dosage information is captured in SPA and SARA for micronutrient supplements, but this information is required to confirm readiness for specific interventions (e.g., vitamin A dosing varies across children 6–11 months, children 12–59 months, and pregnant women). The SPA and SARA counselling items were also too broad to capture nutrition intervention specific messaging.

There were also several nutrition interventions that were not included within the current SPA and SARA, as evidenced by a lack of data on service readiness (commodities that would be required for the interventions) and provision of care (observations of what was provided). Both the SPA and SARA lack essential core items for the following nutrition interventions: calcium supplementation, balanced energy and protein supplementation, multiple micronutrient supplementation, treatment of noncomplicated severe acute malnutrition, inpatient treatment of complicated severe acute malnutrition, and multiple micronutrient powder supplementation.

### 3.3 Expert survey results

Of the 92 individuals who received the survey, 30 fully completed it and three partially completed it. The section on nutrition for pregnant women was completed by 24 respondents, whereas 25 respondents completed the section on nutrition for children under 5. Respondents were from 12 countries, with the majority based in North America (*n* = 9) and sub‐Saharan Africa (*n* = 13). Respondents were from a variety of organizations including academic institutions (*n* = 10), donor agencies (*n* = 5), UN agencies (*n* = 3), Ministry of Health (*n* = 1), and nongovernmental organizations headquarters (*n* = 2) and in‐country offices (*n* = 6).

**Figure 1:**
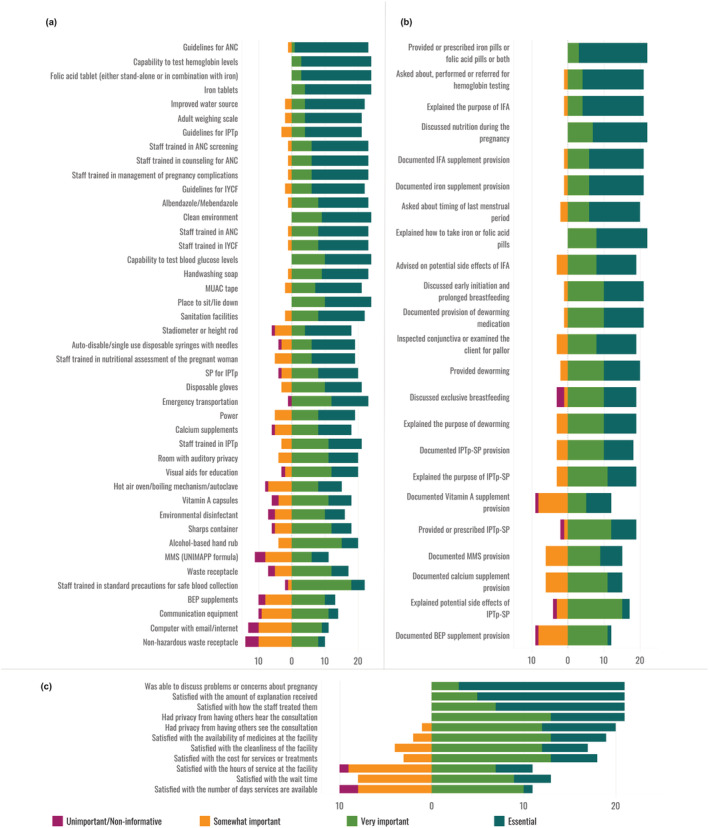
Likert scores for items related to nutrition interventions for pregnant women; (a) services readiness items, (b) provision of care items, (c) experience of care items

Likert scores for each item for pregnant women are presented in Figure 1. Across all interventions for pregnant women, the proportion of respondents categorizing an item as essential ranged from 92% (*n* = 22) to 5% (*n* = 1). In the dimension of service readiness, guidance documents and staff training items had the highest number of “essential” responses. Within the provision of care dimension, items from the nutrition assessment domain were deemed essential by the most respondents. Looking at the prioritization by intervention, across both service readiness and provision of care, items relating to IFA supplementation, assessment and treatment of anaemia, and deworming were the interventions that received the most “essential” responses. Experience of care items that touched upon the communication between the care provider and the client, such as feeling comfortable discussing problems or concerns with the provider, being satisfied with how the staff treated them, and being satisfied with the amount of explanation received about the problem for treatment had the highest number of “essential” responses.

**Figure 2:**
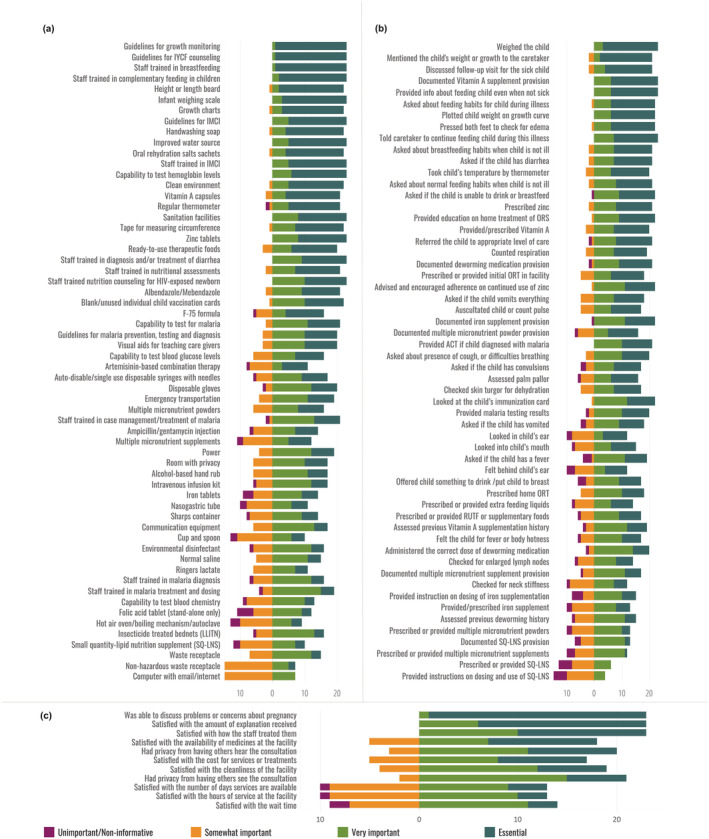
Likert scores for items related to nutrition interventions for children; (a) services readiness items, (b) provision of care items, (c) experience of care items

Likert scores for each item for children under 5 are presented in Figure 2. Across all interventions for children under 5, the proportion of respondents categorizing an item as essential ranged from 96% (*n* = 22) to 0% (*n* = 0). Similar to nutrition for pregnant women, items in the guidance documents and staff training domains had the highest number of “essential” responses. By intervention, service readiness items related to growth monitoring and promotion, and complementary feeding received a high number of “essential” responses whereas provision of care items related to breastfeeding and infant and young child feeding (IYCF) counselling, assessment of illness and feeding during illness, and growth monitoring and promotion received a high number of “essential” responses. The same experience of care items that had the highest number of “essential” responses for pregnant women also had the highest number of “essential” responses for children under 5.

The Sutrop scores presented in Table 1 suggest four different typologies of prioritization based on frequency and relative rank of items: (1) nonessential, ranked low; (2) nonessential, ranked high; (3) essential, rank variably; and (4) essential, rank high. Type 1 were deemed essential by few individuals and overall ranked poorly against other items. Most of these items were for general health services not specific to nutrition interventions, (e.g., waste receptacles) or for interventions not generally considered specific to nutrition (e.g., malaria diagnostic and treatment). Type 2 were items deemed essential by few individuals, but also ranked highly by these respondents (e.g., environmental disinfectant). Type 3 were items deemed essential by most respondents and were ranked variably (e.g., guidance documents). Types 2 and 3 indicate a lack of consensus around the importance of general service delivery items. Type 4 were items deemed essential by most respondents and ranked very highly, indicating a general consensus that these items are critical to include in a nutrition QoC measure. The majority of these items were related to commodity driven nutrition specific interventions, for example, iron and folic acid.

For both pregnant women and children under 5, the service readiness items show little trend in prioritization by domain; however, the provision of care items consistently show a low prioritization of documentation items. The same experience of care item, the client's ability to discuss problems or concerns with the provider, had the highest Sutrop score of all experience of care items for both pregnant women (0.6465) and children under 5 (0.5148). For pregnant women, readiness and provision of care items related to IFA supplementation and anaemia assessment and treatment were among the highest Likert and Sutrop scores. For childhood interventions, readiness items related to growth monitoring and promotion and treatment of diarrhoea with both ORS and zinc were among the highest Sutrop scores while the provision of care items related to breastfeeding and IYCF counselling, assessment of illness and feeding during illness, and growth monitoring and promotion had the highest Sutrop scores.

### 3.4 Proposed nutrition QoC indices for pregnant women and children

After excluding items that fell in the bottom tertile of the range of Sutrop scores, the maternal QoC indices had 40 items and the child QoC indices had 31 items. For the maternal QoC indices, all type 1 items were excluded, all type 4 items were included, and the majority of the type 3 items were included. In addition, only one type 2 item was included as it was deemed essential by few respondents but very highly ranked. For the child QoC indices, all type 1 and type 2 items were excluded, whereas the majority of type 4 and some type 3 items were included.

To ensure consistency between readiness and provision of care items, we added two provision of care items back into the pregnancy index (“provided albendazole or mebendazole,” and “provided or prescribed preventative treatment: IPTp‐SP”). Similarly, four provision of care items were added back into the child index (“prescribed home ORT,” “provided or prescribed RUTF or supplementary food,” “provided or prescribed Vitamin A,” and “prescribed zinc”).

Finally, the research team identified one pregnancy intervention (breastfeeding promotion) and two child health interventions (assessment and treatment of anaemia and deworming) that were excluded from the QoC scores as key interventions for child nutritional status. Therefore two items for breastfeeding promotion (“discussed exclusive breastfeeding” and “discussed early initiation and prolonged breastfeeding”), two items related to assessment and treatment of anemia (“iron tablets,” “assessed palm pallor”), two items related to deworming for children (“albendazole/mebendazole,” “administered deworming medication”), and one item related to counselling on feeding for diarrhoea (“told the caregiver to continue feeding the child during illness”) were added back into the QoC indices.

Table 2 presents the full set of items included in the nutrition QoC indices, and Table 3 presents the items by nutrition intervention. For pregnant women, the final QoC indices included eight interventions (Box 1). Excluded interventions include multiple micronutrient supplementation (MMS), maternal balanced energy and protein supplementation, and maternal vitamin A supplementation. The final service readiness index for pregnant women includes 29 items and the provision and experience of care index for pregnant women includes 15 items. For children under 5, the final QoC indices includes 11 interventions (Box 1). Excluded interventions include inpatient treatment of complicated SAM, preventative iron supplementation in children age 6–23 months, preventative iron supplementation in children aged 2–12 years of age, multiple micronutrient powder (MNP) for children 6–12 months, multiple micronutrient supplementation in different forms for children 2–12 years of age, and SQ‐LNS. The service readiness index for children under 5 includes 19 items, and the provision and experience of care index for children under 5 includes 21 items.

**Table 2:** Proposed items in the nutrition quality of care indices for pregnant women and children under five

Dimension	Domain	Item	Data availability notes
**Pregnant women**			
Service readiness	Basic amenities	Clean environment	Not collected in SARA
	Basic amenities	Power	
	Basic amenities	Sanitation facilities	
	Basic amenities	Emergency transportation	
	Basic amenities	Place for women to sit/lie down	Not collected in SARA
	Basic amenities	Improved water source	
	Equipment and supplies	Disinfectant (environmental)	SARA only asks about this item in the OPD
	Equipment and supplies	Auto‐disable syringes with needles; single use standard disposable syringes with needles	
	Equipment and supplies	Disposable gloves	SARA only asks about this item in the OPD
	Equipment and supplies	Handwashing soap	SARA only asks about this item in the OPD
	Equipment and supplies	Stadiometer or height rod	Not collected in SARA; collected for some SPAs
	Equipment and supplies	MUAC tape^a^	Not collected in SARA or SPA
	Equipment and supplies	Adult weighing scale	
	Medicines and commodities	Calcium supplements^a^	Not collected in SARA or SPA
	Medicines and commodities	Albendazole/Mebendazole	
	Medicines and commodities	Sulfadoxine‐pyrimethamine (SP) for IPTp	Collected only for malaria endemic countries
	Medicines and commodities	Iron tablets (stand‐alone tablets)	
	Medicines and commodities	Folic acid tablet (either stand‐alone or in combination with iron)	
	Diagnostics	Blood glucose levels	
	Diagnostics	Haemoglobin levels	
	Guidelines	Guidelines for antenatal care (ANC)	
	Guidelines	Guidelines for infant and young child feeding (IYCF)	Collected in SPA only for countries with HIV prevalence
	Guidelines	Guidelines for intermittent preventive treatment of malaria during pregnancy	Collected only for malaria endemic countries
	Staff trained	Staff trained in the past 2 years in antenatal care (broad ANC training)	
	Staff trained	Staff trained in the past 2 years in ANC screening (e.g., blood pressure, urine glucose, and protein)	Not collected in SARA
	Staff trained	Staff trained in the past 2 years in counselling for ANC (e.g., nutrition, family planning and newborn care)	Not collected in SARA
	Staff trained	Staff trained in the past 2 years in nutritional assessment of the pregnant woman, such as body mass index calculation and mid‐upper arm circumference measurement	Not collected in SARA
	Staff trained	Staff trained in the past 2 years in infant and young child feeding	
	Staff trained	Staff trained in the past 2 years in complications of pregnancy and their management	Not collected in SARA
Provision of care	Assessment	Inspected conjunctiva or examined the client for pallor	Not collected in SARA
	Assessment	Asked about when the clients last menstrual period began^b^	Not collected in SARA
	Assessment	Asked about, performed or referred the client for haemoglobin testing	Not collected in SARA
	Intervention	Provided albendazole or mebendazole	Not collected in SARA
	Intervention	Discussed exclusive breastfeeding	Not collected in SARA
	Intervention	Provided or prescribed preventive treatment: IPTp‐SP	Not collected in SARA; collected only for malaria endemic countries
	Intervention	Discussed early initiation and prolonged breastfeeding	Not collected in SARA
	Intervention	Advised on potential side effects of IFA	Not collected in SARA
	Intervention	Explained how to take iron or folic acid pills	Not collected in SARA
	Intervention	Explained the purpose of iron or folic acid	Not collected in SARA
	Intervention	Discussed nutrition (i.e., quantity or quality of food to eat) during the pregnancy	Not collected in SARA
	Intervention	Provided or prescribed iron pills or folic acid pills or both	Not collected in SARA
Experience of care	Experience of care	Client satisfied with the amount of explanation received about the problem or treatment	Not collected in SARA
	Experience of care	Client satisfied with how the staff treated them	Not collected in SARA
	Experience of care	Client is able to discuss problems or concerns about pregnancy with provider	Not collected in SARA
**Children under 5**			
Service readiness	Basic amenities	Improved water source	
	Equipment and supplies	Handwashing soap	
	Equipment and supplies	Blank/unused individual child vaccination cards or booklets	
	Equipment and supplies	Growth charts	
	Equipment and supplies	Height or length board	
	Equipment and supplies	Infant weighing scale (100‐gram graduation)	
	Equipment and supplies	Regular thermometer	
	Equipment and supplies	MUAC tape^a^	Not collected in SARA or SPA
	Medicines and commodities	Albendazole/Mebendazole	
	Medicines and commodities	Iron tablets (stand‐alone tablets)	
	Medicines and commodities	Oral rehydration salts (ORS) sachets	
	Medicines and commodities	Ready‐to‐use therapeutic foods^a^	Not collected in SARA or SPA
	Medicines and commodities	Vitamin A capsules	
	Medicines and commodities	Zinc tablets	
	Guidelines	Guidelines for growth monitoring in children	
	Guidelines	Guidelines for infant and young child feeding counselling	Collected within the context of PMTCT service delivery
	Staff trained	Staff trained in the past 2 years in integrated Management of Childhood Illness (IMCI)	
	Staff trained	Staff trained in the past 2 years in complementary feeding in children	
	Staff trained	Staff trained in the past 2 years in breastfeeding	
	Staff trained	Staff trained in the past 2 years in micronutrient deficiencies and/or nutritional assessment	
Provision of care	Assessment	Asked about feeding or breastfeeding habits or practices for child during illness	Not collected in SARA
	Assessment	Asked about normal feeding habits or practices when the child is not ill	Not collected in SARA
	Assessment	Asked if the child is unable to drink or breastfeed	Not collected in SARA
	Assessment	Assessed palm pallor	Not collected in SARA
	Assessment	Pressed both feet to check for edema	Not collected in SARA
	Assessment	Took child's temperature by thermometer	Not collected in SARA
	Assessment	Weighed the child	Not collected in SARA
	Intervention	Administered deworming medication	Not collected in SARA
	Intervention	Mentioned the child's weight or growth to the caretaker, or discussed growth chart	Not collected in SARA
	Intervention	Plotted child weight on growth curve	Not collected in SARA
	Intervention	Prescribed home ORT	Not collected in SARA
	Intervention	Prescribed or provided RUTF or supplementary foods^a^	Not collected in SARA or SPA
	Intervention	Prescribed zinc	Not collected in SARA
	Intervention	Provided general information about feeding or breastfeeding the child even when not sick	Not collected in SARA
	Intervention	Provided/prescribed vitamin A	Not collected in SARA
	Intervention	Told the caretaker to continue feeding the child during this illness	Not collected in SARA
Experience of care	Experience of care	Client satisfied with how the staff treated them	Not collected in SARA
	Experience of care	Client satisfied with the amount of explanation received about the problem or treatment	Not collected in SARA
	Experience of care	Client is able to discuss problems or concerns about child's illness with provider	Not collected in SARA

^a^ Items to be excluded if operationalizing indices with SPA data because item not collected in SPA questionnaire.

^b^ Items to be excluded if operationalizing indices with SPA data because intervention not captured in SPA questionnaire.

**Table 3:** Proposed items in the nutrition quality of care indices for pregnant women and children under five by intervention

Intervention	Dimension	Domain	Item (italicized items are included once in the score but correspond to multiple interventions)
**Pregnant women**			
Assessment and treatment of anaemia during pregnancy	Service readiness	Basic amenities	*Clean environment*
		Basic amenities	*Place for women to sit/lie down*
		Basic amenities	Power
		Diagnostics	Haemoglobin levels
		Equipment and supplies	*Auto‐disable syringes with needles; single use standard disposable syringes with needles*
		Equipment and supplies	*Disinfectant (environmental)*
		Equipment and supplies	*Disposable gloves*
		Equipment and supplies	*Handwashing soap*
		Medicines and commodities	Iron tablets (stand‐alone tablets)
		Guidelines	Counselling for ANC (e.g., nutrition, FP and newborn care)
		Guidelines	*Guidelines for antenatal care (ANC)*
		Staff trained	*Staff trained in the past 2 years in ANC screening (*e.g.*, blood pressure, urine glucose, and protein)*
		Staff trained	*Staff trained in the past 2 years in antenatal care (broad ANC training)*
	Provision of care	Assessment	Asked about, performed or referred the client for haemoglobin testing
		Assessment	Inspected conjunctiva or examined the client for pallor
		Intervention	*Advised on potential side effects of IFA*
		Intervention	*Discussed nutrition (*i.e.*, quantity or quality of food to eat) during the pregnancy*
		Intervention	*Explained how to take iron or folic acid pills*
		Intervention	*Explained the purpose of iron or folic acid*
		Intervention	*Provided or prescribed iron pills or folic acid pills or both*
Blood glucose testing during pregnancy	Service readiness	Basic amenities	*Clean environment*
		Basic amenities	*Place for women to sit/lie down*
		Equipment and supplies	*Auto‐disable syringes with needles; single use standard disposable syringes with needles*
		Equipment and supplies	*Disinfectant (environmental)*
		Equipment and supplies	*Disposable gloves*
		Equipment and supplies	*Handwashing soap*
		Diagnostics	Blood glucose levels
		Guidelines	*Guidelines for antenatal care (ANC)*
		Staff trained	*Staff trained in the past 2 years in ANC screening (*e.g.*, blood pressure, urine glucose, and protein)*
Calcium supplementation during pregnancy	Service readiness	Medicines and commodities	Calcium supplements
		Guidelines	*Guidelines for antenatal care (ANC)*
		Staff trained	*Staff trained in the past 2 years in antenatal care (broad ANC training)*
		Staff trained	Staff trained in the past 2 years in complications of pregnancy and their management
		Staff trained	*Staff trained in the past 2 years in counselling for ANC (*e.g.*, nutrition, family planning and newborn care)*
	Provision of care	Assessment	Asked about when the clients last menstrual period began
		Intervention	*Discussed nutrition (*i.e.*, quantity or quality of food to eat) during the pregnancy*
Daily IFA during pregnancy	Service readiness	Medicines and commodities	*Folic acid tablet (either stand‐alone or in combination with iron)*
		Guidelines	*Guidelines for antenatal care (ANC)*
		Staff trained	*Staff trained in the past 2 years in antenatal care (broad ANC training)*
		Staff trained	*Staff trained in the past 2 years in counselling for ANC (*e.g.*, nutrition, family planning and newborn care)*
	Provision of care	Intervention	*Advised on potential side effects of IFA*
		Intervention	*Discussed nutrition (*i.e.*, quantity or quality of food to eat) during the pregnancy*
		Intervention	*Explained how to take iron or folic acid pills*
		Intervention	*Explained the purpose of iron or folic acid*
		Intervention	*Provided or prescribed iron pills or folic acid pills or both*
Deworming in pregnant women	Service readiness	Medicines and commodities	Albendazole/Mebendazole
		Guidelines	*Guidelines for antenatal care (ANC)*
	Provision of care	Intervention	Provided albendazole or mebendazole
Intermittent IFA during pregnancy	Service readiness	Medicines and commodities	*Folic acid tablet (either stand‐alone or in combination with iron)*
		Guidelines	*Guidelines for antenatal care (ANC)*
		Staff trained	*Staff trained in the past 2 years in antenatal care (broad ANC training)*
		Staff trained	*Staff trained in the past 2 years in counselling for ANC (*e.g.*, nutrition, family planning and newborn care)*
	Provision of care	Intervention	*Advised on potential side effects of IFA*
		Intervention	*Discussed nutrition (*i.e.*, quantity or quality of food to eat) during the pregnancy*
		Intervention	*Explained how to take iron or folic acid pills*
		Intervention	*Explained the purpose of iron or folic acid*
		Intervention	*Provided or prescribed iron pills or folic acid pills or both*
IPT during pregnancy	Service readiness	Medicines and commodities	Sulfadoxine‐pyrimethamine (SP) for IPTp
		Guidelines	Guidelines for intermittent preventive treatment of malaria during pregnancy
	Provision of care	Intervention	Provided or prescribed preventive treatment: IPTp‐SP
Nutrition education and counselling during pregnancy	Service readiness	Equipment and supplies	MUAC tape
		Equipment and supplies	Stadiometer or height rod
		Equipment and supplies	Adult weighing scale
		Guidelines	*Guidelines for antenatal care (ANC)*
		Guidelines	Guidelines for infant and young child feeding counselling (IYCF)
		Staff trained	Staff trained in the past 2 years in infant and young child feeding
		Staff trained	*Staff trained in the past 2 years in counselling for ANC (*e.g.*, nutrition, family planning and newborn care)*
	Provision of care	Intervention	Discussed early initiation and prolonged breastfeeding
		Intervention	Discussed exclusive breastfeeding
		Intervention	*Discussed nutrition (*i.e.*, quantity or quality of food to eat) during the pregnancy*
General items for antenatal care nutrition interventions	Service readiness	Basic amenities	Sanitation facilities
		Basic amenities	Emergency transportation
		Basic amenities	Improved water source
		Equipment and supplies	Disinfectant (environmental)
	Experience of care	Experience of care	Client satisfied with the amount of explanation received about the problem or treatment
		Experience of care	Client satisfied with how the staff treated them
		Experience of care	Client is able to discuss problems or concerns about pregnancy with provider
**Children under 5**			
Assessment and treatment of anaemia	Service readiness	Medicines and commodities	Iron tablets (stand‐alone tablets)
		Staff trained	*Staff trained in the past 2 years in integrated Management of Childhood Illness (IMCI)*
	Provision of care	Assessment	Assessed palm pallor
Complementary feeding counselling	Service readiness	Staff trained	Staff trained in the past 2 years in complementary feeding in children
		Guidelines	Guidelines for infant and young child feeding counselling
	Provision of care	Assessment	*Asked about feeding or breastfeeding habits or practices for child during illness*
		Assessment	Asked about normal feeding habits or practices when the child is not ill
		Intervention	*Provided general information about feeding or breastfeeding the child even when not sick*
		Intervention	*Told the caretaker to continue feeding the child during this illness*
Counselling on feeding for diarrhoea	Provision of care	Assessment	*Asked about feeding or breastfeeding habits or practices for child during illness*
		Intervention	*Told the caretaker to continue feeding the child during this illness*
Deworming in young children aged 12–59 months	Service readiness	Medicines and commodities	Albendazole/Mebendazole
		Staff trained	*Staff trained in the past 2 years in integrated Management of Childhood Illness (IMCI)*
	Provision of care	Intervention	Administered deworming medication
Growth monitoring and promotion	Service readiness	Equipment and supplies	Blank/unused individual child vaccination cards or booklets
		Equipment and supplies	Growth charts
		Equipment and supplies	*Height or length board*
		Equipment and supplies	Infant weighing scale (100‐gram graduation)
		Equipment and supplies	*MUAC tape*
		Guidelines	Guidelines for growth monitoring in children
		Staff trained	*Staff trained in the past 2 years in integrated Management of Childhood Illness (IMCI)*
		Staff trained	*Staff trained in the past 2 years in micronutrient deficiencies and/or nutritional assessment*
	Provision of care	Intervention	Mentioned the child's weight or growth to the caretaker, or discussed growth chart
		Intervention	Plotted child weight on growth curve
		Intervention	*Provided general information about feeding or breastfeeding the child even when not sick*
		Intervention	*Weighed the child*
Oral rehydration solution (ORS) during diarrhoea	Service readiness	Equipment and supplies	*Infant weighing scale (100‐gram graduation)*
		Medicines and commodities	Oral rehydration salts (ORS) sachets
	Provision of care	Assessment	*Asked about feeding or breastfeeding habits or practices for child during illness*
		Assessment	*Asked if the child is unable to drink or breastfeed*
		Assessment	*Pressed both feet to check for edema*
		Assessment	*Took child's temperature by thermometer*
		Intervention	Prescribed home ORT
Postnatal breastfeeding counselling (for early and exclusive breastfeeding, and PMTCT)	Service readiness	Staff trained	Staff trained in the past 2 years in breastfeeding
	Provision of care	Assessment	*Asked about feeding or breastfeeding habits or practices for child during illness*
		Intervention	*Provided general information about feeding or breastfeeding the child even when not sick*
Screening of acute malnutrition	Service readiness	Equipment and supplies	Handwashing soap
		Equipment and supplies	*Height or length board*
		Equipment and supplies	*Infant weighing scale (100‐gram graduation)*
		Equipment and supplies	Regular thermometer
		Equipment and supplies	*MUAC tape*
		Staff trained	*Staff trained in the past 2 years in micronutrient deficiencies and/or nutritional assessment*
	Provision of care	Assessment	*Pressed both feet to check for edema*
		Assessment	*Took child's temperature by thermometer*
Treatment of noncomplicated SAM	Service readiness	Equipment and supplies	*Infant weighing scale (100‐gram graduation)*
		Equipment and supplies	*MUAC tape*
		Medicines and commodities	Ready‐to‐use therapeutic foods
		Staff trained	*Staff trained in the past 2 years in integrated Management of Childhood Illness (IMCI)*
		Staff trained	*Staff trained in the past 2 years in micronutrient deficiencies and/or nutritional assessment*
	Provision of care	Intervention	Prescribed or provided RUTF or supplementary foods
Vitamin A supplementation in children aged 6–59 months	Service readiness	Medicines and commodities	Vitamin A capsules
		Staff trained	*Staff trained in the past 2 years in integrated Management of Childhood Illness (IMCI)*
	Provision of care	Intervention	*Provided general information about feeding or breastfeeding the child even when not sick*
		Intervention	Provided/prescribed vitamin A
Zinc treatment for diarrhoea	Service readiness	Equipment and supplies	*Infant weighing scale (100‐gram graduation)*
		Medicines and commodities	Zinc tablets
		Staff trained	*Staff trained in the past 2 years in integrated Management of Childhood Illness (IMCI)*
	Provision of care	Assessment	*Asked if the child is unable to drink or breastfeed*
		Assessment	*Took child's temperature by thermometer*
		Assessment	*Weighed the child*
		Intervention	Prescribed zinc
		Intervention	*Pressed both feet to check for edema*
General items for child nutrition interventions	Service readiness	Basic amenities	Improved water source
	Experience of care	Experience of care	Client satisfied with how the staff treated them
		Experience of care	Client satisfied with the amount of explanation received about the problem or treatment
		Experience of care	Client is able to discuss problems or concerns about child's illness with provider

**Box 1: Interventions included within the QoC indices**

**Pregnancy interventions**

Assessment and treatment of anaemia
Blood glucose testing
Calcium supplementation
Daily IFA
Intermittent IFA
Nutrition education and counselling during pregnancy
Intermittent preventive treatment for malaria in pregnant women (IPTp)
Deworming

**Children under 5 interventions**

Postnatal breastfeeding counselling (for early and exclusive breastfeeding and PMTCT)
Complementary feeding counselling
Assessment and treatment of anaemia
Deworming in children 12–59 months
Growth monitoring and promotion
Screening for acute malnutrition
Vitamin A supplementation in children 6–59 months
Treatment of non‐complicated SAM
Counselling on feeding for diarrhoea
ORS during diarrhoea
Zinc treatment for diarrhoea

### 3.5 Operationalizing nutrition QoC indices

For both pregnant women and child nutrition indices, we found that items were relatively evenly distributed across domains. Therefore, we recommend a simple additive approach for combining items into a single index score as it is easier for countries to operationalize and it is consistent with other studies which have taken a similar approach for developing QoC indices for antenatal care and child health (Kruk et al., 2016; Nguhiu et al., 2017; O'Neill et al., 2013; Sheffel et al., 2019). The nutrition QoC indices will need to be adapted based on the data source used and the country context. Additional information on operationalizing the nutrition QoC indices is provided in Table 2 and in the Supporting Information.

## 4 DISCUSSION

Our summary indices for nutrition QoC provide a new holistic approach to evaluating facility‐based nutritional care in LMICs in a standardized way using existing data. Through a rigorous multistep process, we have proposed two nutrition QoC indices that can be operationalized by countries with health facility assessment data such as the SPA or SARA. Use of these QoC metrics at a country level can help identify weaknesses in nutrition service delivery and guide strategies for quality improvement.

Our results showed that many evidence‐based nutrition interventions delivered through the health system, such as calcium supplementation during pregnancy and inpatient treatment of complicated severe acute malnutrition, are not reflected in the SPA or SARA. Several of the items needed for the delivery of these interventions were highly prioritized for inclusion in a QoC measure for nutrition interventions by our expert survey yet are not in the assessments or are only partially assessed. In addition, the proposed nutrition QoC indices include several items that are not currently collected in SPA and SARA (e.g., MUAC tape and ready‐to‐use therapeutic foods). Our findings highlight the gaps in existing facility‐based surveys to fully capture the availability, readiness, and provision of key nutrition interventions. While investments in delivery of nutrition interventions to key populations grows, many LMICs have insufficient data to monitor and evaluate their implementation (Gillespie et al., 2019). The ongoing revisions to the SPA and SARA surveys are opportunities to improve the tools and address nutrition monitoring gaps (USAID Advancing Nutrition, 2021; WHO, 2021).

We found the items prioritized for inclusion in the proposed QoC indices reflect more commonly implemented nutrition interventions, yet are not those with the strongest effectiveness. For example, in the child nutrition indices there are six items specific to growth monitoring, a very common intervention across LMICs, but one with questionable evidence of effectiveness (Ashworth et al., 2008). Furthermore, the representation of interventions in our indices is a function of the availability of data in the existing health facility assessments. Items relating to interventions such as assessment of anaemia during pregnancy, provision of IFA during pregnancy, growth monitoring, and assessment of acute malnutrition were well captured in the health facility assessments and were also prioritized for inclusion in our final indices. As global health has embraced the data revolution, there has been an increased focus on the use of high‐quality data to ensure data‐driven decision making. As a result, program planning and implementation are inextricably linked to monitoring and evaluation and information availability (Foreit et al., 2006). In the case of nutrition interventions, data availability for a set of commonly implemented interventions may be emphasizing the implementation of these interventions, and overlooking others due to a lack of data. In order to drive country level political commitment for scaling nutrition interventions, more effort is needed to ensure that data on coverage and quality are available across nutrition interventions (Heidkamp et al., 2021; International Food Policy Research Institute, 2014).

Our process for developing the nutrition QoC indices identified substantial gaps in implementation guidance for nutrition interventions delivered through the health system. Most nutrition interventions delivered through the health system include some component of behaviour change (Pelto et al., 2016; USAID, 2017). Many infant and young child feeding interventions are exclusively counselling based, but even the provision of a supplement requires establishing and maintaining a new behaviour of taking the supplement daily. However, with the exception of recent WHO guidance on breastfeeding counselling guidance, we found that guidance for behaviour change counselling interventions was insufficient (WHO, 2018b). Research on behaviour change for nutrition specific interventions has focused primarily on breastfeeding and complementary feeding counselling, and to a lesser extent behaviour change to ensure effective adherence to prenatal supplements (IFA or MMS) (Gomes et al., 2021; Baker et al., 2013; Graziose et al., 2018; Lamstein et al., 2014; Webb Girard et al., 2020). While counselling programs may differ by context, there is an urgent need for global guidance translating this research to program implementation guidance that can be adapted at country level in order to achieve effective nutrition behaviour change counselling in LMICs and delivery of high‐quality nutrition interventions (Sanghvi et al., 2013). The development of such global guidance is necessary before any assessment of QoC can be standardized.

Findings from our study are subject to some important limitations. First, there was a degree of subjectivity required to identify the sets of interventions considered for inclusion in the indices. To address this challenge, we conducted a literature review and systematically applied inclusion criteria to all interventions under consideration. Second, the proposed QoC indices only capture nutrition interventions delivered through health facilities despite an increasing use of community‐based delivery mechanisms (e.g., for acute malnutrition screening and treatment) (López‐Ejeda et al., 2018). Community‐based services are not captured in current global health facility assessments or other QoC studies. Further efforts are needed in this area. Third, our proposed QoC indices contain several items that are not in the current SPA or SARA surveys. Despite the lack of data availability for some items, we have highlighted how to operationalize the proposed QoC scores using existing SPA and SARA data and ongoing revisions to the SPA and SARA surveys may fill some of these data gaps.

## 5 CONCLUSION

The development of the nutrition QoC indices for pregnant women and children under 5 that reflect global guidelines and expert opinion is an important step towards improving availability of data on nutrition intervention quality that will enable LMIC governments to prioritize and allocate resources towards more effective services. Future research is needed to apply these indices to country data, operationalize the nutrition QoC indices for pregnant women and children under 5, and evaluate the proposed indices for construct validity and reliability. Efforts towards applying findings to guide evidence‐informed decision making must also be prioritized. Improving the QoC of facility‐based nutrition interventions is vital to ensuring the health sector is effectively contributing to a multisectoral approach of reducing malnutrition in LMICs.

## CONFLICTS OF INTEREST

The authors declare that they have no conflicts of interest.

## CONTRIBUTIONS

AS, MKM, and RH contributed to conceptualizing the paper and analysis. SK and YYX conducted the analysis. SK and AS prepared the manuscript. MKM, RH, SW, and YYX critically reviewed and revised the manuscript.

## Supporting information


**Figure S1:** Nutrition intervention selection process
**Table S1:** Nutrition interventions identified through literature review
**Table S2:** Items identified within implementation guidance
**Table S3:** Tabulation of item matching from intervention guidelines to SPA/SARA items

**Data S1.** Supporting Information

## Data Availability

The data that support the findings of this study are openly available in the Harvard Dataverse at https://doi.org/10.7910/DVN/3YYCLE.
