# Supplementary material for: Advancing nutrition measurement: Developing quantitative measures of nutrition service quality for pregnant women and children in low‐ and middle‐income country health systems
Source: Matern Child Nutr. 2021 Nov 3;18(1):e13279. doi: 10.1111/mcn.13279 (PMC8710116; doi:10.1111/mcn.13279)
Supplement: Supplementary file 1 — Figure S1: Nutrition intervention selection process Table S1: Nutrition interventions identified through literature review Table S2: Items identified within implementation guidance Table S3: Tabulation of item matching from intervention guidelines to SPA/SARA items [file MCN-18-e13279-s002.pdf]

Supplementary material: Literature review strategy

An initial literature search of nutrition interventions delivered in LMICs was conducted through PubMed. Search terms included "nutrition interventions", "*nutrition care", "stunting interventions" "micronutrient interventions", "malnutrition interventions", "non-communicable disease intervention", "management of non-communicable disease" along with a LMIC filter. Seminal papers within the LMIC nutrition literature (including the 2008 and 2013 Lancet series) that were not identified within the search were further reviewed and hand-searched to identify other relevant sources. The World Health Organization's e-Library of Evidence for Nutrition Actions was reviewed and all interventions whose guidelines were approved by the WHO Guideline Review Committee were included within the initial intervention identification process. Subsequently WHO guidance documents were reviewed to identify key interventions to address common nutrition problems in LMICs, such as anemia, NCDs, diarrhea, and acute malnutrition. Both nutrition-sensitive and nutrition-specific interventions were identified, without regard to the sector responsible for implementation or the target population. All identified documents were reviewed, and nutrition interventions included within the documents were extracted.

Supplementary Figure 1: Nutrition intervention selection process

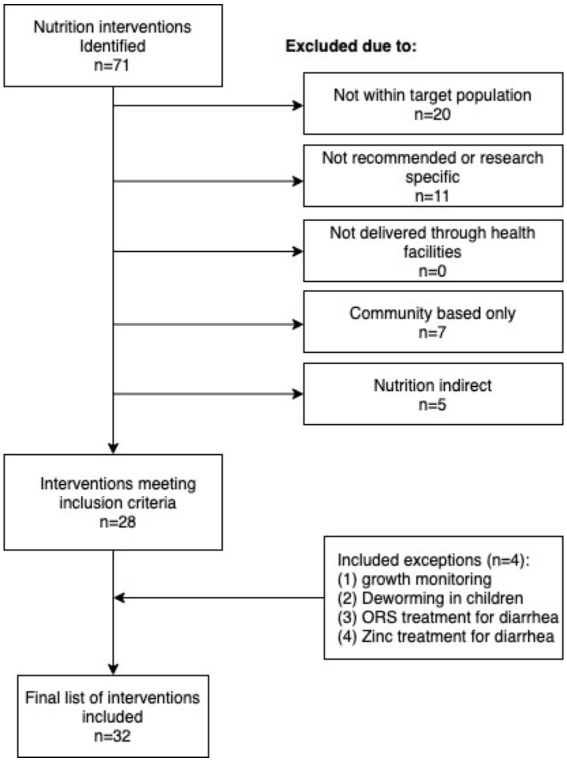

Supplementary Table 1: Nutrition interventions identified through literature review

No	Target group	Intervention	Level of recommendation	Description	Sector responsible				Delivery mechanism		Selection	Reference
				**Intervention description**	**Nutrition direct**	**Nutrition indirect**	**Health sector**	**Other sector**	**Facility**	**Community**	**Met criteria** (* denotes included exception)	
1	**Pregnancy**	Assessment and treatment of anemia during pregnancy	Context specific (WHO)	During pregnancy, women should be assessed for anemia during ANC visits using full blood count or onsite hemoglobin testing with hemoglobinometer. Women who diagnosed with anemia should receive 120mg of elemental iron daily until hemoglobin level returns to above 110g/L	√		√		√		√	WHO recommendations on antenatal care for a positive pregnancy experience. www.who.int/reproductive health/publications/maternal_perinatal_health/anc-positive-pregnancy-experience/en/
2	**Pregnancy**	Blood glucose testing during pregnancy	Recommended (WHO)	During pregnancy, women should receive testing to diagnose gestational diabetes mellitus, or diabetes mellitus in pregnancy using fasting plasma glucose, 1-hour or 2-hour post 75 g oral glucose load.	√		√		√		√	
3	**Pregnancy**	Calcium supplementation during pregnancy	Context specific (WHO)	In populations with low dietary calcium intake, during pregnancy it is recommended women receive a daily supplement of 1.5-2.0 oral elemental calcium to reduce adverse gestational outcomes.	√		√		√	√	√	
4	**Pregnancy**	Daily IFA during pregnancy	Recommended (WHO)	During pregnancy, women should receive daily oral supplementation of 30mg to 60mg of elemental iron and 0.4mg folic acid can improve maternal and perinatal outcomes.	√		√		√	√	√	
5	**Pregnancy**	Intermittent IFA during pregnancy	Context specific (WHO)	Where anemia prevalence among pregnant women is less than 20% , intermittent oral supplementation of 120mg elemental iron and 2.8mg folic acid once weekly can be implemented in lieu of daily supplements to improve maternal and perinatal outcomes. Pregnant women who are intolerant to side-effects of daily iron supplementation may use the same intermittent dosing regimen.	√		√		√	√	√	
6	**Pregnancy**	MMS during pregnancy	Not recommended (WHO) but WHO makes provision for country adoption	Provision of a daily multiple micronutrient supplements containing 13-15 micronutrients including iron and folic acid can be considered in lieu of iron folic acid can improve maternal and perinatal outcomes.	√		√		√	√	√	

No	Target group	Intervention	Level of recommendation	Description	Sector responsible				Delivery mechanism		Selection	Reference
				**Intervention description**	**Nutrition direct**	**Nutrition indirect**	**Health sector**	**Other sector**	**Facility**	**Community**	**Met criteria** (* denotes included exception)	
7	**Pregnancy**	Maternal balanced energy and protein supplementation	Context specific (WHO)	Balanced energy and protein dietary supplements (supplements in which protein provides less than 25% of the energy) is provided to pregnant women in undernourished populations to promote gestational weight gain and improve pregnancy outcomes.	√		√		√	√	√	
8	**Pregnancy**	High-protein supplementation during pregnancy	Not recommended (WHO)	High-protein supplements can improve protein intake amongst pregnant women in undernourished populations to improve maternal and perinatal outcomes.	√		√		√	√		
9	**Pregnancy**	Vitamin A supplementation during pregnancy	Context specific (WHO)	In areas where vitamin A deficiency is highly prevalent, a dose of up to 10,000 IU of Vitamin A per day, or weekly dose of up to 25,000 IU of Vitamin A can prevent night blindness in pregnant women.	√		√		√	√	√	
10	**Pregnancy**	Vitamin B6 supplementation during pregnancy	Not recommended (WHO)	Oral supplementation of Vitamin B6 during pregnancy may alleviate nausea during pregnancy.	√		√		√	√		
11	**Pregnancy**	Zinc supplementation during pregnancy	Not recommended (WHO)/ Research specific/	Oral supplementation of zinc during pregnancy to reduce risk of pre-term birth.	√		√		√	√		
12	**Pregnancy**	Vitamin D supplementation during pregnancy	Not recommended (WHO)	Oral supplementation of Vitamin D during pregnancy to improve perinatal outcomes.	√		√		√	√		
13	**Pregnancy**	Vitamin E and C supplementation during pregnancy	Not recommended (WHO)	Vitamin E and C supplementation is not recommended for pregnant women to improve maternal and perinatal outcomes.	√		√		√	√		

No	Target group	Intervention	Level of recommendation	Description	Sector responsible				Delivery mechanism		Selection	Reference
				**Intervention description**	**Nutrition direct**	**Nutrition indirect**	**Health sector**	**Other sector**	**Facility**	**Community**	**Met criteria** (* denotes included exception)	
14	**Pregnancy**	Nutrition education and counselling during pregnancy	Recommended/ content of counselling is context specific (WHO)	Nutrition education and counselling is recommended to promote healthy eating and keeping physically active during pregnancy to stay healthy and prevent excess weight gain during pregnancy. Topics included in counselling should be tailored to the population and can include diet quality, quantity and diversity, breastfeeding, caffeine reduction, micronutrient supplement use, fortified foods and food supplements.	√		√		√		√	
15	**Pregnancy**	Intermittent preventive treatment during pregnancy	Context specific (WHO)	All pregnant women should receive intermittent preventive treatment with sulfadoxine-pyrimethamine (IPTp-SP) in malaria endemic areas in Africa. Three doses of treatment should be provided at least one month apart, starting the second trimester. This intervention must be delivered along comprehensive malaria prevention strategies.	√		√		√		√	
16	**Pregnancy**	Deworming in pregnant women	Context specific (WHO)	In areas where the baseline prevalence of hookworm and/or T. trichiura infection among pregnancy women is 20% or more, and where anaemia prevalence among pregnant women is 40% or higher, women should receive preventive chemotherapy (deworming), using single-dose albendazole (400 mg) or mebendazole (500 mg) after the first trimester in order to prevent infection and malnutrition.	√		√		√		√	

No	Target group	Intervention	Level of recommendation	Description	Sector responsible				Delivery mechanism		Selection	Reference
				**Intervention description**	**Nutrition direct**	**Nutrition indirect**	**Health sector**	**Other sector**	**Facility**	**Community**	**Met criteria** (* denotes included exception)	
17	**Pregnancy**	MNP for point-of-use fortification of foods consumed by pregnant women	Not recommended (WHO)	Substituting daily iron and folic supplementation with multiple micronutrient powders during pregnancy to improve maternal and infant health outcomes has been considered but is not recommended.	√		√			√		Use of multiple micronutrient powders for point-of-use fortification of foods consumed by pregnant women guideline https://apps.who.int/iris/bitstream/handle/10665/204639/9789241549516_eng.pdf?sequence=1<br><br>Multiple micronutrient powders for home (point-of-use) fortification of foods in pregnant women https://www.cochranelibrary.com/cdsr/doi/10.1002/14651858.CD011158.pub2/full
18	**Pregnancy**	Vitamin A supplementation in postpartum women	Not recommended (WHO)	Postpartum Vitamin A supplementation to increase Vitamin A content of breast milk for the prevention of maternal and infant morbidity and mortality.	√		√		√	√		Guideline: Vitamin A supplementation in postpartum women https://apps.who.int/iris/bitstream/handle/10665/44623/9789241501774_eng.pdf?sequence=1

No	Target group	Intervention	Level of recommendation	Description	Sector responsible				Delivery mechanism		Selection	Reference
				**Intervention description**	**Nutrition direct**	**Nutrition indirect**	**Health sector**	**Other sector**	**Facility**	**Community**	**Met criteria** (* denotes included exception)	
19	**Pregnancy**	Delaying age at first pregnancy	Recommended (WHO)	Strengthening measures to delay age at first pregnancy including prevention of early marriage and childbearing especially before the age of 19.		√	√	√		√		Association between maternal age at childbirth and child and adult outcomes in the offspring: a prospective study in five low-income and middle-income countries (COHORTS collaboration) https://www.thelancet.com/journals/langlo/article/PIIS2214-109X(15)00038-8/fulltext<br><br>Guidelines for preventing early pregnancy and poor reproductive outcomes among adolescents in developing countries https://www.who.int/immunization/hpv/target/preventing_early_pregnancy_and_poor_reproductive_outcomes_who_2006.pdf
20	**Pregnancy**	Birth spacing	Recommended (WHO)	Pregnancies should be spaced 24 months apart in order to reduce the risk of adverse maternal, perinatal and infant outcomes.		√	√	√		√		Report of a WHO Technical Consultation on Birth Spacing https://apps.who.int/iris/bitstream/handle/10665/69855/WHO_RHR_07.1_eng.pdf?sequence=1<br><br>Family Planning Improves Nutrition, Evidence from Studies in Low and Middle-Income Countries https://www.healthpolicyproject.com/pubs/691_FPandNutritionBrief.pdf

No	Target group	Intervention	Level of recommendation	Description	Sector responsible				Delivery mechanism		Selection	Reference
				**Intervention description**	**Nutrition direct**	**Nutrition indirect**	**Health sector**	**Other sector**	**Facility**	**Community**	**Met criteria** (* denotes included exception)	
21	**Infant/Newborn**	Delayed cord clamping	Recommended (WHO)	Delayed cord clamping by more than one minute after the birth will allow blood flow to continue between the placenta and neonate, which can improve iron status in the infant for up to six months after birth.	√		√		√	√	√	Delayed umbilical cord clamping for improved maternal and infant health and nutrition outcomes https://apps.who.int/iris/bitstream/handle/10665/148793/9789241508209_eng.pdf?ua=1<br><br>WHO recommendations Intrapartum care for a positive childbirth experience https://apps.who.int/iris/bitstream/handle/10665/260178/9789241550215-eng.pdf;jsessionid=7E800B590A164DC7FC879E73B480D6FC?sequence=1

No	Target group	Intervention	Level of recommendation	Description	Sector responsible				Delivery mechanism		Selection	Reference
				**Intervention description**	**Nutrition direct**	**Nutrition indirect**	**Health sector**	**Other sector**	**Facility**	**Community**	**Met criteria** (* denotes included exception)	
22	**Infant/Newborn**	Vitamin K administration	Recommended (WHO)	To prevent haemorrhagic disease, all newborns should receive 1 mg of vitamin K intramuscularly (IM) prophylactically after the first hour of birth.	√		√		√		√	WHO recommendations Intrapartum care for a positive childbirth experience https://apps.who.int/iris/bitstream/handle/10665/260178/9789241550215-eng.pdf;jsessionid=7E800B590A164DC7FC879E73B480D6FC?sequence=1<br><br>WHO Recommendations for management of common childhood conditions: evidence for technical update of pocket book recommendations https://apps.who.int/iris/bitstream/handle/10665/44774/9789241502825_eng.pdf?sequence=1<br><br>Controversies Concerning Vitamin K and the Newborn https://pediatrics-aappublications-org.proxy1.library.jhu.edu/content/112/1/191.long

No	Target group	Intervention	Level of recommendation	Description	Sector responsible				Delivery mechanism		Selection	Reference
				**Intervention description**	**Nutrition direct**	**Nutrition indirect**	**Health sector**	**Other sector**	**Facility**	**Community**	**Met criteria** (* denotes included exception)	
23	**Infant/Newborn**	Kangaroo mother care	Recommended (WHO)	Newborns weighing 2000 g or less at birth should initiate continuous kangaroo mother in health-care facilities as soon as the newborns are clinically stable as a way to provide thermal care and support early initiation of breastfeeding to improve perinatal and infant outcomes.	√		√		√		√	WHO recommendations on interventions to improve preterm birth outcomes https://apps.who.int/iris/bitstream/handle/10665/183037/9789241508988_eng.pdf?sequence=1<br><br>The effects of kangaroo mother care on the time to breastfeeding initiation among preterm and LBW infants: a meta-analysis of published studies https://internationalbreastfeedingjournal.biomedcentral.com/articles/10.1186/s13006-019-0206-0

No	Target group	Intervention	Level of recommendation	Description	Sector responsible				Delivery mechanism		Selection	Reference
				**Intervention description**	**Nutrition direct**	**Nutrition indirect**	**Health sector**	**Other sector**	**Facility**	**Community**	**Met criteria** (* denotes included exception)	
24	**Infant/Newborn**	Skin-to-skin contact	Recommended (WHO)	To promote early initiation of breastfeeding and prevent hypothermia, stable newborns should maintain skin-to-skin contact with mothers in the first hour after birth.	√		√		√		√	WHO Recommendations for management of common childhood conditions: evidence for technical update of pocket book recommendations https://apps.who.int/iris/bitstream/handle/10665/44774/9789241502825_eng.pdf?sequence=1<br><br>WHO recommendations Intrapartum care for a positive childbirth experience https://apps.who.int/iris/bitstream/handle/10665/260178/9789241550215-eng.pdf;jsessionid=7E800B590A164DC7FC879E73B480D6FC?sequence=1<br><br>UNICEF Implementation guide for Baby Friendly Hospital Initiative https://www.unicef.org/nutrition/files/Baby-friendly-Hospital-Initiative-implementation-2018.pdf

No	Target group	Intervention	Level of recommendation	Description	Sector responsible				Delivery mechanism		Selection	Reference
				**Intervention description**	**Nutrition direct**	**Nutrition indirect**	**Health sector**	**Other sector**	**Facility**	**Community**	**Met criteria** (* denotes included exception)	
25	**Infant/Newborn**	Vitamin A supplementation in neonates	Not recommended (WHO)	Supplementing Vitamin A to reduce mortality and morbidity amongst neonates up to 28 days of age.	√		√		√			Guideline: Neonatal vitamin A supplementation https://apps.who.int/iris/bitstream/handle/10665/44626/9789241501798_eng.pdf?sequence=1<br><br>Neonatal vitamin A supplementation for the prevention of mortality and morbidity in term neonates in low and middle income countries https://www.cochranelibrary.com/cdsr/doi/10.1002/14651858.CD006980.pub3/full
26	**Child (1-5 months)**	Vitamin A supplementation in infants 1-5 months of age	Not recommended (WHO)	Supplementing Vitamin A to reduce mortality and morbidity amongst infants 1–5 months of age.	√		√		√			Vitamin A supplementation for infants 1–5 months of age Guideline https://www.who.int/nutrition/publications/micronutrients/guidelines/vas_infants_1-5/en/<br><br>Vitamin A supplementation for the prevention of morbidity and mortality in infants six months of age or less https://www.cochranelibrary.com/cdsr/doi/10.1002/14651858.CD007480.pub2/full
27	**Child (2-9 months)**	Intermittent preventive treatment for infants 2-9 months	Recommended (WHO)	In sub-Saharan African areas with moderate-to-high malaria transmission and low parasite resistance to sulfadoxine-pyrimethamine, SP-IPTi should be provided along with DTP2/Penta2, DTP3/Penta3 and measles immunization to infants as part of routine EPI. This should be delivered along with a comprehensive package of malaria prevention and control measures.		√	√		√			Intermittent preventive treatment for infants using sulfadoxine-pyrimethamine (IPTi-SP) for malaria control in Africa: implementation field guide https://www.who.int/malaria/publications/atoz/whoivb11_07/en/

No	Target group	Intervention	Level of recommendation	Description	Sector responsible				Delivery mechanism		Selection	Reference
				**Intervention description**	**Nutrition direct**	**Nutrition indirect**	**Health sector**	**Other sector**	**Facility**	**Community**	**Met criteria** (* denotes included exception)	
28	**Child (0-23 month)**	Postnatal breastfeeding counselling (for early and exclusive breastfeeding, and PMTCT)	Recommended (WHO)	Breastfeeding counselling should be provided to all mothers with young children to promote optimal breastfeeding practices for up to 24 months or longer postnatally. Optimal breastfeeding practices implies exclusive breast feeding until 6 months of age and continue frequent and on-demand breast feeding until 2 years of age or beyond.	√		√		√		√	Guideline: counselling of women to improve breastfeeding practices https://apps.who.int/iris/bitstream/handle/10665/280133/9789241550468-eng.pdf?ua=1<br><br>Global strategy for infant and young child feeding https://apps.who.int/iris/bitstream/handle/10665/42590/9241562218.pdf?sequence=1<br><br>UNICEF Implementation guide for Baby Friendly Hospital Initiative https://www.unicef.org/nutrition/files/Baby-friendly-Hospital-Initiative-implementation-2018.pdf
29	**Child (0-23 month)**	Rotavirus and measles vaccinations	Recommended (WHO)	Immunization of rotavirus and measles helps reduce deaths from diarrhoea by preventing rotavirus infection that directly cause diarrhea, and by preventing measles that can lead to complications such as diarrhoea, which both impact child nutrition status.		√	√		√			Diarrhoea: why children are still dying and what can be done. UNICEF/WHO https://www.who.int/maternal_child_adolescent/documents/9789241598415/en/<br><br>Table 1: Summary of WHO Position papers-Recommendation for Routine Immunization (April 2019) https://www.who.int/immunization/policy/Immunization_routine_table1.pdf?ua=1

No	Target group	Intervention	Level of recommendation	Description	Sector responsible				Delivery mechanism		Selection	Reference
				**Intervention description**	**Nutrition direct**	**Nutrition indirect**	**Health sector**	**Other sector**	**Facility**	**Community**	**Met criteria** (* denotes included exception)	
30	**Child (6-59 months)**	Assessment and treatment of anemia in children	Recommended (WHO)	Daily oral iron supplementation is a preventive strategy for implementation at the population level. If a child is diagnosed with anaemia, national guidelines for the treatment of anaemia should be followed.	√		√		√		√	Daily iron supplementation in infants and children https://www.who.int/nutrition/publications/micronutrients/guidelines/daily_iron_supp_childrens/en/
31	**Child (6-23 months)**	Complementary feeding counselling	Recommended (WHO)	Complementary feeding counselling should be provided to mothers of young children to promote optimal complementary feeding practices starting from 6 months of age. Counselling topics include responsive feeding, safe preparation and storage of complementary foods, amount, frequency and content of complementary food, food consistency, vitamin-mineral supplements and fortified foods.	√		√		√		√	Global strategy for infant and young child feeding https://apps.who.int/iris/bitstream/handle/10665/42590/9241562218.pdf?sequence=1<br><br>Guiding principles for complementary feeding of the breastfed child http://iris.paho.org/xmlui/handle/123456789/752<br>UNICEF Programming guide for IYCF https://www.unicef.org/nutrition/files/Final_IYCF_programming_guide_2011.pdf
32	**Child (6-23 months)**	Iron supplementation in children aged 6-23 months	Recommended (WHO)	In settings where the prevalence of anemia is 40% or higher in this age group, children should receive daily iron supplementation of 10-12.5mg of elemental iron for three consecutive months in a year for preventing iron deficiency and anemia.	√		√		√	√	√	Daily iron supplementation in infants and children https://www.who.int/nutrition/publications/micronutrients/guidelines/daily_iron_supp_childrens/en/

No	Target group	Intervention	Level of recommendation	Description	Sector responsible				Delivery mechanism		Selection	Reference
				**Intervention description**	**Nutrition direct**	**Nutrition indirect**	**Health sector**	**Other sector**	**Facility**	**Community**	**Met criteria** (* denotes included exception)	
33	**Child (6-23 months)**	SQ-LNS for children	Recommended (Cochrane)	Small quantity lipid nutrition supplement (SQ-LNS) are food products which contain energy, protein, essential fatty acids, minerals and vitamins. Within these products, the majority of the energy comes from lipids and are intended to be provided in conjunction with local complementary foods, not as a replacement. Provision of SQ-LNS with complementary feeding can improve growth outcomes and reduce the occurrence of stunting, moderate underweight, moderate wasting, and anaemia especially when supplemented for a duration of more than 12 months.	√		√		√	√	√	Effect of lipid-based nutrient supplementation on infants and young children https://www.cochrane.org/CD012611/BEHAV_effect-lipid-based-nutrient-supplementation-infants-and-young-children
34	**Child (6-23 months)**	Multiple Micronutrient Powder for children aged 6-23 months	Recommended (WHO)	In populations where the prevalence of anemia in children is 20% or higher, point-of-use fortification of complementary foods with iron-containing micronutrient powders of 90 sachets given over a period of 6 months in children aged 6–23 months is recommended, to improve iron status and reduce anemia. Each sachet contains:10-12.5 mg of elemental iron; 300 µg of retinol; 5mg of zinc; with or without other micronutrients.	√		√		√	√	√	Use of multiple micronutrient powders for point-of-use fortification of foods consumed by infants and young children aged 6–23 months and children aged 2–12 years https://apps.who.int/iris/bitstream/handle/10665/252540/9789241549943-eng.pdf?ua=1

No	Target group	Intervention	Level of recommendation	Description	Sector responsible				Delivery mechanism		Selection	Reference
				**Intervention description**	**Nutrition direct**	**Nutrition indirect**	**Health sector**	**Other sector**	**Facility**	**Community**	**Met criteria** (* denotes included exception)	
35	**Child (12-59 months)**	Deworming in young children aged 12-59 months	Recommended (WHO)	In areas where the baseline prevalence of any soil-transmitted infection is between 20-50%, children between the age of 12-59 months should receive annual preventive chemotherapy (deworming) of a dose of albendazole (400mg) or mebendazole (500mg) in order to reduce the worm burden of soil-transmitted helminths. In areas where the baseline prevalence is 50% or more, all children aged 12-59 months should receive bi-annual preventive chemotherapy. Note children aged 12-23 should only receive half dose of albendazole (200mg).		√	√		√		√*	Preventive chemotherapy to control soil-transmitted helminth infections in at-risk population groups https://apps.who.int/iris/bitstream/handle/10665/258983/9789241550116-eng.pdf?sequence=1
36	**Child (6-59 months)**	Growth monitoring	Not recommended (WHO)	Growth monitoring is the regular collection of data on a child's anthropometry including weight and height. Thi s information is plotted as a growth curve and compared to growth standards to assess status of child growth and identify children experiencing growth faltering prior to becoming under-nourished.	√		√		√	√	√*	Growth monitoring and promotion: review of evidence of impact https://onlinelibrary.wiley.com/doi/full/10.1111/j.1740-8709.2007.00125.x<br><br>The WHO Child Growth Standards https://www.who.int/childgrowth/standards/en/<br><br>Revisiting the concept of growth monitoring and its possible role in the community-based nutrition programs https://journals.sagepub.com/doi/pdf/10.1177/156482651103200105

No	Target group	Intervention	Level of recommendation	Description	Sector responsible				Delivery mechanism		Selection	Reference
				**Intervention description**	**Nutrition direct**	**Nutrition indirect**	**Health sector**	**Other sector**	**Facility**	**Community**	**Met criteria** (* denotes included exception)	
37	**Child (6-59 months)**	Screening of acute malnutrition	Recommended (WHO)	Children aged 6-59 months should be screened for acute malnutrition to identify both severe acute malnutrition and moderately acute malnutrition. In community settings, trained community health workers and community members should measure the mid-upper arm circumference, while in primary health-care facilities and hospitals, health-care workers should assess the mid-upper arm circumference or the weight-for-height/weight-for-length status. In both settings infants and children should be examined for bilateral pitting oedema.	√		√		√	√	√	WHO child growth standards and the identification of severe acute malnutrition in infants and children https://apps.who.int/iris/bitstream/handle/10665/44129/9789241598163_eng.pdf?ua=1
38	**Child (6-59 months)**	Vitamin A supplementation in children aged 6-59 months	Recommended (WHO)	In settings where the prevalence of night blindness or vitamin A deficiency is a public health problem, all children aged 6-59 months should receive high-dose oral vitamin A supplementation. Infants aged 6-11 months should receive 100,000 IU once, and young children aged 12- 59 months should receive a dose of 200,000 IU every 4-6 months.	√		√		√		√	Vitamin A supplementation for infants and children 6-59 months of age https://www.who.int/nutrition/publications/micronutrients/guidelines/vas_6to59_months/en/

No	Target group	Intervention	Level of recommendation	Description	Sector responsible				Delivery mechanism		Selection	Reference
				**Intervention description**	**Nutrition direct**	**Nutrition indirect**	**Health sector**	**Other sector**	**Facility**	**Community**	**Met criteria** (* denotes included exception)	
39	**Child (6-59 months)**	Community-based MAM treatment	Recommended (WHO)	Infants and children aged 6–59 months with moderate acute malnutrition need to consume nutrient-dense foods to meet their extra needs for weight and height gain and functional recovery which is frequently provided through ready-to-use supplementary foods or fortified blended foods. The routine management of MAM cases and distribution of supplementary foods should be carried out at the community level instead of primary health-care facilities.	√		√			√		Technical note: Supplementary foods for the management of moderate acute malnutrition in infants and children 6–59 months of age. https://apps.who.int/iris/bitstream/handle/10665/75836/9789241504423_eng.pdf?sequence=1<br><br>Guideline: assessing and managing children at primary health-care facilities to prevent overweight and obesity in the context of the double burden of malnutrition. Updates for the Integrated Management of Childhood Illness (IMCI) https://apps.who.int/iris/bitstream/handle/10665/259133/9789241550123-eng.pdf?sequence=1<br><br>USAID: Community-based Management of Acute Malnutrition https://www.usaid.gov/sites/default/files/documents/1864/CMAM-technical-guidance-brief-508-revFeb2017.pdf

No	Target group	Intervention	Level of recommendation	Description	Sector responsible				Delivery mechanism		Selection	Reference
				**Intervention description**	**Nutrition direct**	**Nutrition indirect**	**Health sector**	**Other sector**	**Facility**	**Community**	**Met criteria** (* denotes included exception)	
40	**Child (6-59 months)**	Inpatient treatment of complicated SAM	Recommended (WHO)	Children who are identified as having severe acute malnutrition who present with medical complications, severe oedema, fail the appetite test, or present with one or more IMCI danger signs should be treated as inpatients. Practice guidelines for treating SAM in inpatient or emergency feeding centers settings including correcting hypothermia, hypoglycemia, electrolyte imbalance, infection, micronutrient deficiencies, and initiation of cautious feeding using F75 and F-100 formula.	√		√		√		√	Guidelines for the inpatient treatment of severe acute malnutrition https://www.who.int/nutrition/publications/guide_inpatient_text.pdf<br><br>Management of Severe Malnutrition: A Manual for Physicians and Other Senior Health Workers https://apps.who.int/iris/bitstream/handle/10665/41999/a57361.pdf?sequence=1
41	**Child (6-59 months)**	Treatment of non-complicated SAM	Recommended (WHO)	Children who are identified as having severe acute malnutrition who pass the appetite test and are clinically well and alert should be treated as outpatients. Children recovering from inpatient treatment who have resolved medical complications, have good appetite and are alert should be transferred to outpatient treatment. Children identified as requiring outpatient treatment can be treated with ready-to-use therapeutic foods (RUTF) until fully recovered. Dosing of RUTF depends on child size and RUTF product specifications.	√		√		√		√	Guideline: updates on the management of severe acute malnutrition in infants and children. https://apps.who.int/iris/bitstream/handle/10665/95584/9789241506328_eng.pdf?ua=1<br><br>Management of Severe Malnutrition: A Manual for Physicians and Other Senior Health Workers https://apps.who.int/iris/bitstream/handle/10665/41999/a57361.pdf?sequence=1

No	Target group	Intervention	Level of recommendation	Description	Sector responsible				Delivery mechanism		Selection	Reference
				**Intervention description**	**Nutrition direct**	**Nutrition indirect**	**Health sector**	**Other sector**	**Facility**	**Community**	**Met criteria** (* denotes included exception)	
42	**Child (0-5 years)**	Counselling on feeding for diarrhea	Recommended (WHO)	Mothers should receive education and counselling on continued feeding including breastfeeding during diarrhea episodes and the use of appropriate fluids available in home when oral rehydration salt are not available.	√		√		√	√	√	Guiding principles for complementary feeding of the breastfed child http://iris.paho.org/xmlui/handle/123456789/752 Implementing the New Recommendations on the<br><br>Clinical Management of Diarrhoea https://apps.who.int/iris/bitstream/handle/10665/43456/9241594217_eng.pdf?sequence=1<br><br>The treatment of diarrhoea - A manual for physicians and other senior health workers https://apps.who.int/iris/bitstream/handle/10665/43209/9241593180.pdf?sequence=1<br><br>Diarrhoea: Why children are still dying and what can be done? https://apps.who.int/iris/bitstream/handle/10665/44174/9789241598415_eng.pdf?sequence=1

No	Target group	Intervention	Level of recommendation	Description	Sector responsible				Delivery mechanism		Selection	Reference
				**Intervention description**	Nutrition direct	Nutrition indirect	Health sector	Other sector	Facility	Community	Met criteria (* denotes included exception)	
43	**Child (0-5 years)**	Preventative zinc supplementation	Not recommended (WHO)	Preventative zinc supplementation in children under 5 years of age may improve linear growth and reduce infection.	√		√		√			Zinc supplementation for preventing mortality, morbidity, and growth failure in children aged 6 months to 12 years of age https://www.cochranelibrary.com/cdsr/doi/10.1002/14651858.CD009384.pub2/full<br><br>Zinc supplementation for the prevention of pneumonia in children aged 2 months to 59 months https://www.cochranelibrary.com/cdsr/doi/10.1002/14651858.CD005978.pub3/full
44	**Child (0-5 years)**	Oral rehydration solution (ORS) during diarrhea	Recommended (WHO)	Children experiencing dehydration due to diarrhea should receive oral rehydration therapy (ORT) with ORS solution in a health facility. Health workers should education parents on proper preparation of ORS.		√	√		√	√	√*	Implementing the New Recommendations on the Clinical Management of Diarrhoea https://apps.who.int/iris/bitstream/handle/10665/43456/9241594217_eng.pdf?sequence=1<br><br>The treatment of diarrhoea A manual for physicians and other senior health workers https://apps.who.int/iris/bitstream/handle/10665/43209/9241593180.pdf?sequence=1<br><br>Proposal to include an additional listing of co-packaged ORS and zinc for management of diarrhea in children on the WHO Model List of Essential Medicines for Children

No	Target group	Intervention	Level of recommendation	Description	Sector responsible				Delivery mechanism		Selection	Reference
				**Intervention description**	**Nutrition direct**	**Nutrition indirect**	**Health sector**	**Other sector**	**Facility**	**Community**	**Met criteria** (* denotes included exception)	
45	**Child (0-5 years)**	Zinc treatment for diarrhea	Recommended (WHO)	Children experiencing diarrhea should receive 20 mg per day of zinc supplementation for 10-14 days (10 mg per day for infants under the age of six months) provided by health care worker to reduce duration and severity of diarrhea episode.		√	√		√	√	√*	Implementing the New Recommendations on the Clinical Management of Diarrhoea https://apps.who.int/iris/bitstream/handle/10665/43456/9241594217_eng.pdf?sequence=1<br><br>The treatment of diarrhoea A manual for physicians and other senior health workers https://apps.who.int/iris/bitstream/handle/10665/43209/9241593180.pdf?sequence=1
46	**Child (2-12 years)**	Iron supplementation in young children aged 2-12 years of age	Recommended (WHO)	In settings where the prevalence of anemia in children aged 2-12 years is 40% or higher, children should receive daily iron supplementation of iron (30mg elemental iron for children 2-5 years; 30-60mg elemental iron for children 5-12 years) for three consecutive months in a year to prevent iron deficiency and anaemia.<br>In settings where the prevalence of anemia is between 20% - 40%, children should receive intermittent iron supplementation(25 mg elemental iron for children 2-5 years, and 45 mg elemental iron for children 5-12 years) once per week for 3 months, followed by 3 months of no supplementation before restarting the cycle of supplementation.	√		√		√	√	√	Daily iron supplementation in infants and children Guideline https://apps.who.int/iris/bitstream/handle/10665/204712/9789241549523_eng.pdf?sequence=WHO.<br><br>Guideline: Intermittent iron supplementation in preschool and school-age children https://apps.who.int/iris/bitstream/handle/10665/44648/9789241502009_eng.pdf;jsessionid=2C3ACE8238101EA26EDEEF3E091A43DA?sequence=1

No	Target group	Intervention	Level of recommendation	Description	Sector responsible				Delivery mechanism		Selection	Reference
				**Intervention description**	**Nutrition direct**	**Nutrition indirect**	**Health sector**	**Other sector**	**Facility**	**Community**	**Met criteria** (* denotes included exception)	
47	**Child (2-12 years)**	Multiple micronutrient supplementation in different forms for children 2-12 years of age	Recommended (WHO)	In populations where the prevalence of anemia in children is 20% or higher, supplementation of iron-containing multiple micronutrients in different forms. Currently available guidelines include point-of-use fortification with iron-containing micronutrient powders of 90 sachets given over a period of 6 months in children aged 24-59 months is recommended to improve iron status and reduce anemia. Each sachet contains:10-12.5 mg of elemental iron for 2-5 years and 12.5-30.0 mg of elemental iron for 5-12 years; 300 µg of retinol; 5mg of zinc; with or without other micronutrients.	√		√		√	√	√	Use of multiple micronutrient powders for point-of-use fortification of foods consumed by infants and young children aged 6–23 months and children aged 2–12 years https://apps.who.int/iris/bitstream/handle/10665/252540/9789241549943-eng.pdf?ua=1
48	**Child (2-15 years)**	Reducing marketing of food and non-alcoholic beverages on children	Recommended (WHO)	A set of 12 recommendations by WHO, aimed at reducing the impact of marketing foods high in saturated fats, trans-fatty acids, free sugars or salt to children.	√		√	√		√		Set of recommendations on the marketing of foods and non-alcoholic beverages to children https://www.who.int/dietphysicalactivity/publications/recsmarketing/en/
49	**Child (2-15 years)**	Increase potassium intake from food to control blood pressure (in children and adults at risk of CVD)	Recommended (WHO)	Increasing potassium intake from food to control blood pressure in children aged 2–15 years with a recommended intake adjusted based on child's energy requirement using adults intake of at least 90 mmol/day as a upper reference.	√		√			√		Guideline: Potassium intake for adults and children https://apps.who.int/iris/bitstream/handle/10665/77986/9789241504829_eng.pdf?sequence=1
50	**Child (2-15 years)**	Reducing sodium intake to control blood pressure	Recommended (WHO)	Reducing sodium intake to control blood pressure in children aged 2–15 years with a recommended intake adjusted based on child's energy requirement using adult intake of less than 2g/day as a upper reference.	√		√			√		Guideline: Sodium intake for adults and children https://apps.who.int/iris/bitstream/handle/10665/77985/9789241504836_eng.pdf?sequence=1
51	**Child (2-15 years)**	Reducing sugar intake to reduce risk of NCDs	Recommended (WHO)	Reducing intake of free sugars throughout the life course to less than 10% of total energy intake for children.	√		√			√		Guideline: sugars intake for adults and children. https://apps.who.int/iris/bitstream/handle/10665/149782/9789241549028_eng.pdf?sequence=1

No	Target group	Intervention	Level of recommendation	Description	Sector responsible				Delivery mechanism		Selection	Reference
				**Intervention description**	Nutrition direct	Nutrition indirect	Health sector	Other sector	Facility	Community	Met criteria (* denotes included exception)	
52	**Adolescent girls/ WRA**	Deworming in non-pregnant adolescent girls and WRA	Recommended (WHO)	In areas where prevalence of any soil-transmitted helminth infection is 20% or more among adolescent girls and women of reproductive age (aged 12-49 years), preventive chemotherapy (deworming), using annual single-dose albendazole (400 mg) or mebendazole (500 mg), is recommended as a public health intervention for all non-pregnant adolescent girls and women of reproductive age living to reduce the worm burden of soil-transmitted helminths. In areas where the baseline prevalence is 50% or more, all children should receive bi-annual preventive chemotherapy.		√	√		√			Preventive chemotherapy to control soil-transmitted helminth infections in at-risk population groups https://apps.who.int/iris/bitstream/handle/10665/258983/9789241550116-eng.pdf?sequence=1
53	**Adolescent girls/ WRA**	Iron supplementation in adult women and adolescent girls	Recommended (WHO)	In settings where the prevalence of anemia is higher than 40% amongst adult women and adolescent girls, they should receive daily iron supplementation of 30-60mg elemental iron for 3 consecutive months in a year.	√		√		√	√		Guideline: Daily iron supplementation in adult women and adolescent girls https://apps.who.int/iris/bitstream/handle/10665/204761/9789241510196_eng.pdf?sequence=1
54	**Adolescent girls/ WRA**	Malaria prevention in women or pregnant women	Recommended (WHO)	All people who reside in malaria endemic areas, including vulnerable populations such as pregnant women and infants, should receive one long-lasting insecticidal net at point of contact with health facility or health worker.		√	√		√			Insecticide-Treated Nets for the Prevention of Malaria in Pregnancy: A Systematic Review of Randomised Controlled Trials https://journals.plos.org/plosmedicine/article?id=10.1371/journal.pmed.0040107<br><br>Insecticide-treated nets for preventing malaria in pregnancy https://www.cochranelibrary.com/cdsr/doi/10.1002/14651858.CD003755.pub2/full

No	Target group	Intervention	Level of recommendation	Description	Sector responsible				Delivery mechanism		Selection	Reference
				**Intervention description**	**Nutrition direct**	**Nutrition indirect**	**Health sector**	**Other sector**	**Facility**	**Community**	**Met criteria** (* denotes included exception)	
55	**Adults**	Nutrition assessment and counselling for those with tuberculosis	Recommended (WHO)	All individuals with active TB should receive nutrition assessment and appropriate counselling based on their nutritional status at diagnosis and throughout treatment.	√		√		√			Guideline: Nutritional care and support for patients with tuberculosis. https://apps.who.int/iris/bitstream/handle/10665/94836/9789241506410_eng.pdf?sequence=1
56	**Adults**	Supplemental nutrition with dietary advice for older people affected by undernutrition	Recommended (WHO)	Oral nutrition supplemental with dietary advice should be provided for older people affected by undernutrition.	√		√		√			Integrated care for older people: guidelines on community-level interventions to manage declines in intrinsic capacity https://apps.who.int/iris/bitstream/handle/10665/258981/9789241550109-eng.pdf?sequence=1<br><br>Dietary advice with or without oral nutritional supplements for disease-related malnutrition in adults https://www.cochranelibrary.com/cdsr/doi/10.1002/14651858.CD002008.pub4/full
57	**Adults**	Increase potassium intake from food to control blood pressure (in children and adults at risk of CVD)	Recommended (WHO)	Adults should increase potassium intake from foods to at least 90mmol/day to control blood pressure and reduce the risk of cardiovascular disease.	√		√		√	√		Guideline: Potassium intake for adults and children https://apps.who.int/iris/bitstream/handle/10665/77986/9789241504829_eng.pdf?sequence=1
58	**Adults**	Reducing sodium intake to control blood pressure	Recommended (WHO)	Adults should reduce sodium intake to less than 2g/day (5g/day salt) to lower blood pressure and the risk of cardiovascular disease, stroke and coronary heart disease.	√		√	√	√	√		Guideline: Sodium intake for adults and children https://apps.who.int/iris/bitstream/handle/10665/77985/9789241504836_eng.pdf?sequence=1
59	**Adults**	Reducing sugar intake to reduce risk of NCDs	Recommended (WHO)	Adults should reduce intake of free sugars to less than 10% of total energy intake.	√		√	√	√	√		Guideline: sugars intake for adults and children. https://apps.who.int/iris/bitstream/handle/10665/149782/9789241549028_eng.pdf?sequence=1

No	Target group	Intervention	Level of recommendation	Description	Sector responsible				Delivery mechanism		Selection	Reference
				**Intervention description**	**Nutrition direct**	**Nutrition indirect**	**Health sector**	**Other sector**	**Facility**	**Community**	**Met criteria** (* denotes included exception)	
60	**Adults**	Nutrition counselling for reducing risk of multiple NCDs	Recommended	Adults who are at risk for or have non-communicable diseases such as cardiovascular disease, diabetes, hypertension, and dyslipidemia should receive nutrition counselling to improve diets and reduce dietary risk factors. Topics include reducing intake/calories, improved dietary quality and micronutrient intakes, increasing physical activity etc,	√		√		√	√		Joint WHO/FAO Expert Consultation on Diet, Nutrition and the Prevention of Chronic Diseases https://apps.who.int/iris/bitstream/handle/10665/42665/WHO_TRS_916.pdf?ua=1
61	**Adults**	Health education on food hygiene and management	Recommended (WHO)	Adults should receive education on proper food hygiene and management which include:<br>keeping clean; separating raw and cooked; cooking thoroughly ; keeping food at safe temperatures; and safe water and raw materials usage.	√	√	√			√		WHO Five Keys to Safer Food communication campaign - Evidence-based simple messages with a global impact<br><br>https://www.sciencedirect.com/science/article/abs/pii/S0956713519300647
62	**Adults**	Tobacco cessation to reduce NCD risk	Recommended (WHO)	Comprehensive tobacco-control policies must be in place to achieve effective and sustainable smoking cessation which include regulations of a supportive environment, offering of treatment for cessation, availability of national policies, social and community awareness, trained and skilled health professionals, and active multisectoral collaborations.		√						WHO Tools for Advancing Tobacco Control in the XXIst century: Policy Recommendations for Smoking Cessation and Treatment of Tobacco Dependence: Tools for public health https://www.who.int/tobacco/resources/publications/tobacco_dependence/en/
63	**Population level**	Fortification of maize flour and corn meal	Context specific (WHO)	Fortification of maize flour and corn meal with iron can prevent iron deficiency particularly in vulnerable populations such as children and women. Fortification of maize flour and corn meal with folic acid can reduce the risk of occurrence of births with neural tube defects.	√		√	√		√		Fortification of maize flour and corn meal with vitamins and minerals https://www.who.int/nutrition/publications/micronutrients/guidelines/maize-corn-fortification/en/

No	Target group	Intervention	Level of recommendation	Description	Sector responsible				Delivery mechanism		Selection	Reference
				**Intervention description**	**Nutrition direct**	**Nutrition indirect**	**Health sector**	**Other sector**	**Facility**	**Community**	**Met criteria** (* denotes included exception)	
64	**Population level**	Fortification of rice	context specific (WHO)	Fortification of rice with iron, vitamin A, or folic acid can improve micronutrient status of populations, specifically in populations where rice is a staple food.	√		√	√		√		Guideline: fortification of rice with vitamins and minerals as a public health strategy https://apps.who.int/iris/bitstream/handle/10665/272535/9789241550291-eng.pdf?ua=1
65	**Population level**	Fortification of wheat flour	context specific (WHO)	Wheat flour fortification can be used as vehicle for nutrient delivery when industrially produced flour is regularly consumed by the population. Decisions about which nutrients to add and the appropriate amounts to add should be based on local context.	√		√	√		√		e-Library of Evidence for Nutrition Action (eLENA): Fortification of wheat flour https://www.who.int/elena/titles/wheat-flour-fortification/en/
66	**Population level**	Iodized salt	Recommended (WHO)	All food-grade salt, used in household and food processing should be fortified with iodine as a safe and effective strategy for the prevention and control of iodine deficiency disorders in populations living in stable and emergency settings.	√		√	√		√		Guideline: fortification of food-grade salt with iodine for the prevention and control of iodine deficiency disorders. https://apps.who.int/iris/bitstream/handle/10665/136908/9789241507929_eng.pdf?ua=1
67	**Population level**	Dietary diversification and enhancing the bioavailability of micronutrients to address anemia	Recommended (WHO)	Increasing the production and consumption of iron-rich foods (animal-source foods such as red meat, poultry, and fish, and iron-rich plant sources such as legumes) to reduce the prevalence of anemia.	√			√				WHO Nutritional anaemias: Tools for Effective Prevention and Control https://apps.who.int/iris/bitstream/handle/10665/259425/9789241513067-eng.pdf;jsessionid=A302614E1CECFE4E11BAF5A3543C1912?sequence=1
68	**Population level**	Handwashing promotion	Recommended (WHO)	Promoting optimal hand washing practices can reduce infection and diarrhea and prevent malnutrition.		√		√		√		WHO Nutritional anaemias: Tools for Effective Prevention and Control https://apps.who.int/iris/bitstream/handle/10665/259425/9789241513067-eng.pdf;jsessionid=A302614E1CECFE4E11BAF5A3543C1912?sequence=1

No	Target group	Intervention	Level of recommendation	Description	Sector responsible				Delivery mechanism		Selection	Reference
				**Intervention description**	**Nutrition direct**	**Nutrition indirect**	**Health sector**	**Other sector**	**Facility**	**Community**	**Met criteria** (* denotes included exception)	
69	**Population level**	Provision of safe drinking water	Recommended (WHO)	Promoting treatment of water and safe storage of drinking water in the household can reduce infection, diarrhea and prevent malnutrition.		√		√		√		WHO Nutritional anaemias: Tools for Effective Prevention and Control https://apps.who.int/iris/bitstream/handle/10665/259425/9789241513067-eng.pdf;jsessionid=A302614E1CECFE4E11BAF5A3543C1912?sequence=1
70	**Population level**	Access to safely managed sanitation	Recommended (WHO)	Promoting safe, hygienic methods of sanitation and faeces management to reduce infection, diarrhea and prevent malnutrition.		√		√		√		WHO Nutritional anaemias: Tools for Effective Prevention and Control https://apps.who.int/iris/bitstream/handle/10665/259425/9789241513067-eng.pdf;jsessionid=A302614E1CECFE4E11BAF5A3543C1912?sequence=1
71	**Population level**	Promotion of wearing shoes	Recommended (WHO)	Promote wearing of shoes (for control of soil-transmitted helminths) to prevent transmission of soil-transmitted helminths that contribute to malnutrition.		√		√		√		WHO Nutritional anaemias: Tools for Effective Prevention and Control https://apps.who.int/iris/bitstream/handle/10665/259425/9789241513067-eng.pdf;jsessionid=A302614E1CECFE4E11BAF5A3543C1912?sequence=1

Supplementary Table 1: Items identified within implementation guidance

Deleted: 2

Intervention	Service readiness					Provision of care				Experience of care	References
	**Equipment and supplies (includes equipment and infection prevention items)**	**Medicines and commodities**	**Diagnostics**	**HR/Trained staff and guidelines**	**Basic amenities**	**Nutrition assessment (Nutrition focused physical measurement and examination; Laboratory and medical assessment; Client and dietary history)**	**Medical and nutrition diagnosis**	**Nutrition intervention (Food and supplement (or care) provision; Nutrition counselling)**	**Nutrition monitoring and evaluation (Monitoring and record tracking; Follow-up visit)**		
Pregnancy											
Assessment and treatment of anemia during pregnancy	(1) disposable gloves<br>(2) finger pricks or lancets<br>(3) cotton balls<br>(4) disinfectant<br>(5) alcohol-based hand rub<br>(6) handwashing soaps<br>(7) single use standard disposable syringes with needles or<br>(8) auto-disable syringes with needles<br>(9) sharps container<br>(10) hazardous waste receptacle<br>(11) other waste receptacle<br>(12) band-aids or gauze<br>(13) hot air oven/boiling mechanism/autoclave (when disposable is not available/appropriate)<br>(14) behavior change materials/counselling materials	(1) 120mg elemental iron: equals 600 mg of ferrous sulfate heptahydrate, 360 mg of ferrous fumarate or 1000 mg of ferrous gluconate.	(1) hematology analyzer<br>(2) electronic counter (Coulter counter)<br>(3) lab wipes<br>(4) test tubes<br>(5) pipettes<br>(6) dilutants<br>(7) Drabkin solution<br>(8) Haemoglobinometer (HemoCue)<br>(9) microcuvette<br>(10) Haemoglobin colour scale<br>(11) box test strips<br>(12) testing reference values	(1) training in standard precautions for safe blood collection<br>(2) training in anemia diagnosis<br>(3) training in processing and analyzing blood samples<br>(4) training in anemia treatment with iron supplement and correct dosing<br>(5) training in nutrition counselling<br>(6) available guidelines on assessment and treatment of anemia during pregnancy	(1) electricity for coulter counter<br>(2) clean environment<br>(3) place for women to sit/lie down particularly if feeling faint	(1) inspection of conjunctiva or pallor<br>(2) Hemoglobin testing	(1) diagnosing of anemia	(1) provide/ prescribe iron supplement of 120mg elemental iron daily until Hgb rises to 110g/L and resume regular doses based on population recommendations<br><br>Provide, explain, or counsel on:<br>(2) instructions on correct dosing/regime<br>(3) purpose and importance of supplement<br>(4) potential side effects<br>(5) locally available foods high in iron	(1) documentation of supplement provision<br>(2) follow up for re-assessment of Hgb and re-assess continuation of iron dose	(1) the provision of effective clinical practices, relevant and timely information, psychosocial and emotional support by knowledgeable, supportive, and respective staff	[1-5]
Blood glucose testing during pregnancy	(1) disposable gloves<br>(2) cotton balls<br>(3) disinfectant<br>(4) alcohol-based hand rub<br>(5) handwashing soaps<br>(6) single use standard disposable syringes with needles or<br>(7) auto-disable syringes with needles<br>(8) sharps container<br>(9) hazardous waste receptacle<br>(10) other waste receptacle<br>(11) band-aids or gauze<br>(12) hot air oven/boiling mechanism/autoclave (when disposable is not available/appropriate)		(1) glucometer<br>(2) glucometer test strips (within expiration date)<br>(3) 75g oral glucose equivalent	(1) training in standard precautions for safe blood collection<br>(2) training in glucose testing and GDM and DM in pregnancy diagnosis<br>(3) available guidelines on blood glucose testing during pregnancy	(1) clean environment<br>(2) place for women to sit/lie down particularly if feeling faint	(1) fasting plasma glucose testing<br>(2) 1-hour / 2-hour post 75 g oral glucose testing<br>(3) random glucose testing	(1) diagnosing of gestational diabetes, or diabetes during pregnancy		(1) refer to nutrition counselling during pregnancy	(1) the provision of effective clinical practices, relevant and timely information, psychosocial and emotional support by knowledgeable, supportive, and respective staff	[6, 7]
Calcium supplementation during pregnancy	(1) behavior change materials/counselling materials	(1) 1.5-2.0g oral elemental calcium		(1) training in nutrition counselling<br>(2) training counselling on complications of pregnancy and their management<br>(3) training in		(1) asking about stage of pregnancy		(1) Provide/prescribe calcium supplement of:<br>1.5-2.0g/day oral elemental calcium<br><br>Provide, explain, or counsel on:<br>(2) instruction on	(1) documentation of supplement provision	(1) the provision of effective clinical practices, relevant and timely information, psychosocial and emotional support by	[1, 8-10]

Intervention	Service readiness					Provision of care				Experience of care	References
	**Equipment and supplies (includes equipment and infection prevention items)**	**Medicines and commodities**	**Diagnostics**	**HR/Trained staff and guidelines**	**Basic amenities**	**Nutrition assessment (Nutrition focused physical measurement and examination; Laboratory and medical assessment; Client and dietary history)**	**Medical and nutrition diagnosis**	**Nutrition intervention (Food and supplement (or care) provision; Nutrition counselling)**	**Nutrition monitoring and evaluation (Monitoring and record tracking; Follow-up visit)**		
				provision and supplement dosing of calcium supplement<br>(4) available guidelines on calcium supplementation during pregnancy				correct dosing<br>(3) purpose and importance of calcium supplement<br>(4) knowledge on calcium rich foods<br>(5) knowledge about interactions between iron and calcium supplementation<br>(6) consuming calcium with food and in divided doses to improve acceptability<br>(7) improving acceptability and adherence of calcium supplementation		knowledgeable, supportive, and respective staff	
Daily IFA during pregnancy	(1) behavior change materials/counselling materials	IFA 2 formulations:<br>(1) for populations with anemia prevalence of 20% or more: 30mg elemental iron and 400ug folic acid<br>(2) for populations with anemia prevalence of 40% of more: 60mg elemental iron (equivalent to 300 mg of ferrous sulfate heptahydrate, 180 mg of ferrous fumarate or 500 mg of ferrous gluconate) and 400ug folic acid		(1) training in nutrition counselling<br>(2)training in provision and supplement dosing of IFA<br>(3) available guidelines on daily IFA supplementation during pregnancy				(1) Provide/prescribe IFA supplement of: Daily IFA with 30-60mg elemental iron + 400ug folic acid<br><br>Provide, explain, or counsel on:<br>(2) instructions on correct dosing<br>(3) purpose and importance of IFA<br>(4) advise on potential side effects<br>(5) locally available food high in iron<br>(6) addressing barriers to adherence including cultural misconceptions	(1) documentation of supplement provision	(1) the provision of effective clinical practices, relevant and timely information, psychosocial and emotional support by knowledgeable, supportive, and respective staff	[1, 11, 12]
Intermittent IFA during pregnancy	(1) behavior change materials/counselling materials	(1) 120mg elemental iron + 2800 ug folic acid<br>(for		(1) training in nutrition counselling for pregnancy<br>(2) training in		(1) assess intolerance to daily IFA dose due to side-effects		(1) Provide/prescribe IFA supplement of: Weekly IFA with 120mg elemental iron +2800ug folic	(1) documentation of supplement provision	(1) the provision of effective clinical practices, relevant and timely	[1, 13]

Intervention	Service readiness					Provision of care				Experience of care	References
	Equipment and supplies (includes equipment and infection prevention items)	Medicines and commodities	Diagnostics	HR/Trained staff and guidelines	Basic amenities	Nutrition assessment (Nutrition focused physical measurement and examination; Laboratory and medical assessment; Client and dietary history)	Medical and nutrition diagnosis	Nutrition intervention (Food and supplement (or care) provision; Nutrition counselling)	Nutrition monitoring and evaluation (Monitoring and record tracking; Follow-up visit)		
		populations with anemia prevalence less than 20% or if daily iron is not acceptable due to side effects)		provision and supplement regime of IFA<br>(3) available guideline on intermittent IFA during pregnancy				acid.<br><br>Provide, explain, or counsel on:<br>(2) instructions on correct dosing<br>(3) purpose and importance of IFA<br>(4) advise on potential side effects<br>(5) locally available food high in iron<br>(6) addressing barriers to adherence including cultural misconceptions		information, psychosocial and emotional support by knowledgeable, supportive, and respective staff	
MMS during pregnancy	(1) behavior change materials/counselling materials	(1) Multiple Micronutrient Supplement: UN Multiple Micronutrient Preparation (UNIMAP)		(1) training in nutrition counselling for pregnancy<br>(2) training in provision and supplement regime of MMS<br>(3) available guidelines on MMS during pregnancy				(1) Provide/ prescribe MMS<br><br>Provide, explain, or counsel on:<br>(2) instructions on correct dosing<br>(3) purpose and importance of MMS<br>(4) promoting nutrient intake through locally available foods<br>(5) addressing barrier to adherence including cultural misconception	(1) documentation of supplement provision	(1) the provision of effective clinical practices, relevant and timely information, psychosocial and emotional support by knowledgeable, supportive, and respective staff	[1, 14]
Maternal balanced energy and protein supplementation	(1) behavior change materials/counselling materials<br>(2) adult weighing scale<br>(3) adult stadiometer	(1) Balanced Energy and Protein supplement (protein content should provide less than 25% of total energy)	(1) MUAC tape	(1) training in nutrition counselling for pregnancy<br>(2) training in nutritional assessment in pregnant women<br>(3) available guidelines on maternal balanced energy and protein supplement		(1) assessment of MUAC<br>(2) assess BMI	(1) diagnosing of underweight/ Protein-Energy Malnutrition in pregnant women	(1) Provide/prescribe Balanced energy and protein supplement and correct dosing<br><br>Provide, explain, or counsel on:<br>(2) Instructions on correct dosing<br>(3) purpose and importance of balanced energy and protein supplement<br>(4) promoting nutrient intake through locally available foods<br>(5) addressing barrier to adherence including cultural	(1) documentation of supplement provision	(1) the provision of effective clinical practices, relevant and timely information, psychosocial and emotional support by knowledgeable, supportive, and respective staff	[1, 15, 16]

Intervention	Service readiness					Provision of care				Experience of care	References
	Equipment and supplies (includes equipment and infection prevention items)	Medicines and commodities	Diagnostics	HR/Trained staff and guidelines	Basic amenities	Nutrition assessment (Nutrition focused physical measurement and examination; Laboratory and medical assessment; Client and dietary history)	Medical and nutrition diagnosis	Nutrition intervention (Food and supplement (or care) provision; Nutrition counselling)	Nutrition monitoring and evaluation (Monitoring and record tracking; Follow-up visit)		
								misconception			
Vitamin A supplementation during pregnancy	(1) behavior change materials/counselling materials	(1) 10 000IU Vitamin A (daily) or (2) 25 000IU Vitamin A (weekly)		(1) training in nutrition counselling for pregnancy (2) training in provision and supplement regime for Vitamin A (3) available guidelines on Vitamin A supplementation during pregnancy				Provide/prescribe Vitamin A supplement: (1) Daily 10 000 IU Vitamin A or (2) Weekly 25 000 IU Vitamin A<br><br>Provide, explain, or counsel on: (3) Instructions on correct dosing (4) purpose and importance of Vitamin A (5) promoting nutrient intake through locally available foods (6) addressing barriers to adherence including cultural misconception	(1) documentation of supplement provision	(1) the provision of effective clinical practices, relevant and timely information, psychosocial and emotional support by knowledgeable, supportive, and respective staff	[1, 17]
Nutrition education and counselling during pregnancy	(1) behavior change materials/counselling materials (2) MUAC tape (3) Stadiometer (4) Adult weighing scale			(1) training in nutrition counselling for pregnancy (2) availability of standardized guidance on nutrition counselling (3) training in nutritional assessment in pregnant women for Protein-Energy Malnutrition (4) available guidelines for nutrition education and counselling during pregnancy (5) training in breastfeeding counselling		(1) assessment of MUAC (2) assessment of BMI	(1) diagnosing of Protein-Energy Malnutrition	Provide, explain, or counsel on: (1) knowledge on diet adequacy in energy, protein, vitamins and minerals through consuming a variety of foods (diet diversity) (2) knowledge on keeping physically active during pregnancy and excessive weight gain during pregnancy. (3) knowledge on appropriate weight gain (4) increasing energy and protein intake in undernourished mothers (5) addressing barriers to adhering to recommendations (6) cultural appropriate dietary		(1) the provision of effective clinical practices, relevant and timely information, psychosocial and emotional support by knowledgeable, supportive, and respective staff	[1]

Intervention	Service readiness					Provision of care				Experience of care	References
	Equipment and supplies (includes equipment and infection prevention items)	Medicines and commodities	Diagnostics	HR/Trained staff and guidelines	Basic amenities	Nutrition assessment (Nutrition focused physical measurement and examination; Laboratory and medical assessment; Client and dietary history)	Medical and nutrition diagnosis	Nutrition intervention (Food and supplement (or care) provision; Nutrition counselling)	Nutrition monitoring and evaluation (Monitoring and record tracking; Follow-up visit)		
								and physical activity choices<br>(7) anticipate and address important challenges and contexts for BF<br>(8) knowledge on benefits of BF such as protection and comfort as well as food<br>(9) support mothers to make informed choices about other feeding choices, if mother does not breastfeed<br>(10) mothers and their families to start a nurturing, caring and responsive relationship with their infant and aim to enable a positive and loving environment.			
Intermittent preventive treatment during pregnancy		(1) intermittent preventive treatment: sulfadoxine-pyrimethamine		(1) training in Intermittent preventive treatment of malaria in pregnancy<br>(2) available guidelines on intermittent preventive treatment during pregnancy<br>(3) available job-aids and checklist				(1) Provision/ prescription of preventive treatment: IPTp-SP, start in the second trimester, at least 3 doses 1 month apart<br><br>Provide, explain, or counsel on:<br>(1) purpose of drug administration<br>(2) side-effects of drug	(1) documentation of drug provision<br>(2) scheduling return visits	(1) the provision of effective clinical practices, relevant and timely information, psychosocial and emotional support by knowledgeable, supportive, and respective staff	[1, 18]
Deworming in pregnant women		(1) albendazole (400 mg)<br>or<br>(2) mebendazole (500 mg)		(1) training in deworming drug provision in pregnant women<br>(2) training in WASH messaging and counselling<br>(3) available guidelines for deworming in pregnancy women				(1) Provision of albendazole or mebendazole<br><br>Provide, explain, or counsel on:<br>(1) purpose of deworming<br>(2) counselling on health and hygiene such as hand washing, use of footwear and proper disposal of faeces	(1) documentation of drug provision	(1) the provision of effective clinical practices, relevant and timely information, psychosocial and emotional support by knowledgeable, supportive, and respective staff	[19]

Intervention	Service readiness					Provision of care				Experience of care	References
	**Equipment and supplies (includes equipment and infection prevention items)**	**Medicines and commodities**	**Diagnostics**	**HR/Trained staff and guidelines**	**Basic amenities**	**Nutrition assessment (Nutrition focused physical measurement and examination; Laboratory and medical assessment; Client and dietary history)**	**Medical and nutrition diagnosis**	**Nutrition intervention (Food and supplement (or care) provision; Nutrition counselling)**	**Nutrition monitoring and evaluation (Monitoring and record tracking; Follow-up visit)**		
Infant/Newborn											
Delayed cord clamping	(1) sterile surgical blade for cutting the umbilical cord<br>(2) cord clips/clamp<br>(3) time tracker (such as apgar timer, clock/watch, phone, timer etc.)<br>(4) bili-light of bili-blanket (for phototherapy)			(1) training in delayed cord clamping<br>(2) available guidelines for delayed cord clamping	(1) clean environment	(1) assessing need for pressure ventilation by checking if baby is breathing spontaneously after thorough drying<br>(2) assessing for infant jaundice		(1) babies which do not need ventilation, should receive late cord clamping approximately 1-3 minutes after birth<br>(2) treatment of jaundice using phototherapy if needed<br>(3) Explaining relevance and benefit of delayed cord clamping to women and family		(1) respectful maternity care – which refers to care organized for and provided to all women in a manner that maintains their dignity, privacy, and confidentiality, ensures freedom from harm and mistreatment, and enables informed choice and continuous support during labour and childbirth in a culturally appropriate and understandable manner	[20, 21]
Vitamin K administration	(1) single use standard disposable syringes with needles<br>(2) or auto-disable syringes with needles<br>(2) alcohol wipes<br>(3) sharps container<br>(4) hazardous waste receptacle<br>(5) other waste receptacle<br>(6) band-aids or gauze<br>(7) weighing scale for infants	(1) IM Vitamin K: 0.5mg-1.0mg<br>or<br>(2) PO Vitamin K: 2.0mg		(1) training in Vitamin K provision in infants<br>(2) training in standard safety precautions<br>(3) training in counselling related to Vitamin K<br>(4) available guidelines for Vitamin K administration	(1) clean environment	(1) weighing of infant		(1) (preferably) Intramuscular Vitamin K within 6 hours of birth and after stabilization: 0.5mg for infants weighing <=1500g; 1.0mg for infants weighing >1500g. or<br>(2) (if reject IM injection) PO Vitamin K: 2.0mg at the time of the first feeding, to be repeated at 2 to 4 and 6 to 8 weeks of age<br><br>Provide, explain, or counsel on:<br>(3) purpose and importance of IM Vitamin K injections«<br>(4) for parents who decline injection on serious health risk of VKD<br>(5) difference between IM injection and oral supplement	(1) documentation of supplement provision	(1) implement strategies that minimize the procedural pain associated with IM injections<br>(2) obtain consent from parents	[21-23]

Intervention	Service readiness					Provision of care				Experience of care	References
	Equipment and supplies (includes equipment and infection prevention items)	Medicines and commodities	Diagnostics	HR/Trained staff and guidelines	Basic amenities	Nutrition assessment (Nutrition focused physical measurement and examination; Laboratory and medical assessment; Client and dietary history)	Medical and nutrition diagnosis	Nutrition intervention (Food and supplement (or care) provision; Nutrition counselling)	Nutrition monitoring and evaluation (Monitoring and record tracking; Follow-up visit)		
Kangaroo mother care	(1) support binder during KMC<br>(2) feeding utensils<br>(3) weighing scales for infants<br>(4) feeding tubes (#5, or #6)<br>(5) equipment to note volume of milk (graduated cups or 10ml syringes)<br>(6) record keeping items (paper, pen)<br>(7) thermometer for baby<br>(8) resuscitation equipment<br>(9) breast pump<br>(10) behavior change materials/counselling materials	(1) preterm formula		(1) facility KMC policy and protocol<br>(2) training in KMC for low-birth-weight babies<br>(3) training on breastfeeding	(1) beds and curtains with privacy<br>(2) facilities for expression of breast milk<br>(3) food provided for the mother<br>(4) handwashing stations<br>(5) station for safe handling of feeding utensils and linen<br>(6) room heaters as needed<br>(7) rooms with privacy<br>(8) toilet facilities<br>(9) refrigerator	(1) weighing of infant	(1) diagnosing of low birth weight (below <=1200g)	(1) KMC initiated on low-birth-weight babies who are stabilized<br>(2) intermittent KMC if baby is not stabilized while a single session should not be less than 60 minutes<br>(3) early discharge of LBW baby when baby is suckling, growing well and family or mother is demonstrating competency in care<br>(4) recreational, educational, or even income generating activities for mother<br><br>Provide, explain, or counsel on:<br>(5) early initiation of breastfeeding, and breastfeeding at home during KMC<br>(6) continues KMC at home<br>(7) establish agreed upon schedule for follow-up visits<br>(8) misconceptions regarding KMC<br>(9) knowledge on appropriate clothing of baby during KMC<br>(10) positioning of baby during KMC<br>(11) father to provide KMC<br>(12) discourage smoking<br>(13) address misconceptions about breastfeeding	Record tracking on<br>(1) feeding<br>(2) weight<br>(3) Schedule return visit	(1) ensure privacy for care provider during KMC<br>(2) respectful maternity care – which refers to care organized for<br>and provided to all women in a manner that maintains their dignity, privacy, and confidentiality, ensures freedom from harm and mistreatment, and enables informed choice and continuous support during labour and childbirth	[24-26]
Skin-to-skin contact				(1) training in importance of skin-to skin<br>(2) training in breastfeeding<br>(3) available guidelines for skin-				(1) advise mother to initiated skin-to-skin contact within the first hour of birth		(1) respectful maternity care – which refers to care organized for and provided to all women in a manner that	[21, 27-29]

Intervention	Service readiness					Provision of care				Experience of care	References
	Equipment and supplies (includes equipment and infection prevention items)	Medicines and commodities	Diagnostics	HR/Trained staff and guidelines	Basic amenities	Nutrition assessment (Nutrition focused physical measurement and examination; Laboratory and medical assessment; Client and dietary history)	Medical and nutrition diagnosis	Nutrition intervention (Food and supplement (or care) provision; Nutrition counselling)	Nutrition monitoring and evaluation (Monitoring and record tracking; Follow-up visit)		
				to-skin contact						maintains their dignity, privacy, and confidentiality, ensures freedom from harm and mistreatment, and enables informed choice and continuous support during labour and childbirth	
Children											
Postnatal breastfeeding counselling (for early and exclusive breastfeeding, and PMTCT)	(1) behavior change materials/counselling materials			(1) training in breastfeeding counselling<br>(2) nutrition counseling for newborn of mother with HIV/AIDS training<br>(3) trained health-care professional and breastfeeding counsellors.<br>(4) training and mentoring programme for counsellors.<br>(5) specialist capacity to counsel mothers with heightened needs such as stressed or traumatized mothers, malnourished infants and mothers, low-birth-weight infants, infant with disability and feeding difficulties.<br>(6) available guidelines for postnatal breastfeeding counselling		(1) ask about current breastfeeding patterns, practices, and challenges		Provide, explain, or counsel on:<br>(1) knowledge on recognizing the milk ejection reflex (or let-down) effective feedings and understanding feeding patterns and growth spurts.<br>(2) anticipate and address important challenges and contexts for BF<br>(3) knowledge on benefits of BF such as protection and comfort as well as food<br>(4) support mothers to make informed choices about other feeding choices, if mother does not breastfeed<br>(5) mothers and their families to start a nurturing, caring and responsive relationship with their infant and aim to enable a positive and loving environment.<br>(6) support and boosting mother's confidence,<br>(7) address concerns about maintaining breastfeeding during	(1) receive a total of 5 BFC sessions postnatally: during and immediately after birth; at 1-2 weeks after birth; in the first 3-4 months; at 6 months; and after 6 months	(1) not a "top-down" intervention of "telling women what to do".<br>(2) focus on empowering women to breastfeed<br>(3) respecting their personal situations and wishes and never be forced upon any women.<br>(4) positive feedback and emotional support are especially needed to support the mothers' confidence and self-efficacy in breastfeeding<br>(5) BFC should be provided as a continuum of care,<br>(6) face-to-face counselling preferably	[30]

Intervention	Service readiness					Provision of care				Experience of care	References
	Equipment and supplies (includes equipment and infection prevention items)	Medicines and commodities	Diagnostics	HR/Trained staff and guidelines	Basic amenities	Nutrition assessment (Nutrition focused physical measurement and examination; Laboratory and medical assessment; Client and dietary history)	Medical and nutrition diagnosis	Nutrition intervention (Food and supplement (or care) provision; Nutrition counselling)	Nutrition monitoring and evaluation (Monitoring and record tracking; Follow-up visit)		
								potential changes in their situation.			
Complementary feeding counselling	(1) behavior change materials/counselling materials			(1) training in nutrition counselling for complementary feeding and feeding during illness<br>(2) available guidelines for counselling on complementary feeding		(1) ask about current complementary feeding practices<br>(2) ask about feeding during illness		Provide, explain, or counsel on:<br>(1) timely introduction at 6 months<br>(2) adequacy: providing sufficient energy, protein and micronutrient considering, amount, meal frequency and nutrient content, and food consistently<br>(3) safe: hygienically stored and prepared and fed with clean hands and utensils.<br>(4) not using bottles or teats<br>(5) properly fed based on child's signals of appetite and satiety, responsive feeding.<br>(7) low-cost complementary food or industrially processed complementary foods when financially permitting.<br>(8) feeding during illness<br>(9) maintenance of breastfeeding<br>(10) addressing challenges during complementary feeding		(1) Providing sound and culture-specific nutrition counselling including local foods.	[31, 32]
Assessment and treatment of anemia in children	(1) disposable gloves<br>(2) finger pricks or lancets<br>(3) cotton balls<br>(4) disinfectant<br>(5) alcohol-based hand rub<br>(6) handwashing soaps<br>(7) single use standard disposable syringes with needles or<br>(8) auto-disable syringes with needles<br>(9) sharps container	(1) elemental iron (dosing varies by age of the child) in the form of syrup<br>(2) elemental iron (dosing varies by age of the child) in the form of iron folate tablets	(1) hematology analyzer<br>(2) electronic counter (Coulter counter)<br>(3) lab wipes<br>(4) test tubes<br>(5) pipettes<br>(6) dilutants<br>(7) Drabkin solution<br>(8)	(1) training in nutrition counselling<br>(2) training in anemia assessment<br>(3) training in iron supplement provision<br>(4) available guidelines on iron supplementation		(1) look for palmar pallor		(1) Provision/ prescription of iron supplement: a daily dose of 20-50mg elemental iron in the form of tablets/syrup for 14 days<br><br>Provide, explain, or counsel on:<br>(2) Instruction on	(1) documentation of supplement provision<br>(2) schedule return visits<br>(3) refer to appropriate level of care		[33]

Intervention	Service readiness				Provision of care				Experience of care	References
	**Equipment and supplies (includes equipment and infection prevention items)**	**Medicines and commodities**	**Diagnostics**	**HR/Trained staff and guidelines**	**Basic amenities**	**Nutrition assessment (Nutrition focused physical measurement and examination; Laboratory and medical assessment; Client and dietary history)**	**Medical and nutrition diagnosis**	**Nutrition intervention (Food and supplement (or care) provision; Nutrition counselling)**	**Nutrition monitoring and evaluation (Monitoring and record tracking; Follow-up visit)**		
	(10) hazardous waste receptacle<br>(11) other waste receptacle<br>(12) band-aids or gauze<br>(13) hot air oven/boiling mechanism/autoclave (when disposable is not available/appropriate)<br>(14) behavior change materials/counselling materials		Haemoglobinometer (HemoCue)<br>(9) microcuvette<br>(10) Haemoglobin colour scale<br>(11) box test strips<br>(12) testing reference values	and treatment in children				correct dosing.<br>(3) diet diversity, and food combinations that improve iron absorption<br>(4) addressing barriers to adherence and acceptability<br>(5) the importance and purpose of return visits in 14 days			
Deworming in young children aged 12-59 months	Equipment for crushing tablets:<br>(1) in a folded paper using a glass bottle<br>or<br>(2) with a mortar and pestle,<br>or<br>(3) between two spoons.	(1) albendazole (400 mg)<br>or<br>(2) mebendazole (500 mg		(1) training in drug administration<br>(2) training in health and hygiene education<br>(3) available guidelines on deworming in children		(1) assess previous deworming history		(1) drug administration of correct dose: albendazole (400mg) or mebendazole (500mg)<br>Note: (a) Annual dose of for areas where prevalence of any soil-transmitted infection is between 20-50% (b) Biannual dose, in areas where the baseline prevalence is 50% or more, (c) children aged 12-23 should only receive half dose of albendazole (200mg)<br>(2) tablets must be crushed into fine powder before administration for children below 3 months<br>(3) inform children 3-5 years old to chew tablet<br><br>Provide, explain, or counsel on:<br>(4) knowledge on health and hygiene<br>(5) knowledge on purpose and benefits of deworming	(1) documentation of supplement provision		[19, 34]
Growth monitoring	(1) measuring tape<br>(2) electronic weighing scale (precision of 0.1kg and up to 150kg, and allows tared weighing)			(1) training in proper anthropometry measure		(1) assess height/length<br>(2) assess weight<br>(3) plotting out growth	(1) identifying growth faltering up until age 18	Provide, explain, or counsel on:<br>(1) growth chart interpretation	(1) documentation of growth curve and faltering if any in child booklet	(1) providing respectful and welcoming services	[35-37]

Intervention	Service readiness					Provision of care				Experience of care	References
	Equipment and supplies (includes equipment and infection prevention items)	Medicines and commodities	Diagnostics	HR/Trained staff and guidelines	Basic amenities	Nutrition assessment (Nutrition focused physical measurement and examination; Laboratory and medical assessment; Client and dietary history)	Medical and nutrition diagnosis	Nutrition intervention (Food and supplement (or care) provision; Nutrition counselling)	Nutrition monitoring and evaluation (Monitoring and record tracking; Follow-up visit)		
	(3) length boards<br>(4) heigh board<br>(5) reference growth chart<br>(6) child booklet			(2) training in growth monitoring, interpretation, and planning<br>(3) available guidelines for growth monitoring in children		curve	months	(2) discussing options with caregivers on care plan<br>(3) discuss optimal child feeding practices	(2) monitoring weight until 12 months of age. Where there are episodes of growth faltering continue to monitor until18 months		
Screening of acute malnutrition	(1) measuring tape<br>(2) Electronic weighing scale (precision of 0.1kg and up to 150kg, and allows tared weighing)<br>(3) Length boards<br>(4) height board<br>(5) MUAC tape<br>(6) regular thermometer<br>(7) disposable gloves<br>(8) cotton balls<br>(9) disinfectant<br>(10) alcohol-based hand rub<br>(11) handwashing soaps<br>(12) single use standard disposable syringes with needles or<br>(13) auto-disable syringes with needles<br>(14) sharps container<br>(15) hazardous waste receptacle<br>(16) other waste receptacle<br>(17) band-aids or gauze<br>(18) hot air oven/boiling mechanism/autoclave (when disposable is not available/appropriate)		(1) glucometer<br>(2) glucometer test strips (within expiration date)<br>(3) Electrolyte blood test (Na, K, Mg)<br>(4) onsite blood test analysis for blood chemistry	(1) training in SAM screening and assessment<br>(2) training in proper anthropometric measurement<br>(3) available guidelines for screening of acute malnutrition	(1) clean environment	(1) assessing for bilateral pitting oedema<br>(2) assess blood glucose<br>(3) assess electrolytes<br>(4) measure temperature<br>(5) conduct appetite test<br>(6) assess IMCI danger signs (unable to drink or breastfeed, history of convulsions, lethargy or unconsciousness, currently convulsing, history of vomiting everything etc.)<br>(7) assess other medical complications and comorbidities (hypoglycemia, hypothermia, dehydration, electrolyte imbalance, infection, history of TB, HIV)	(1) severe acute malnutrition (WHZ <-3 SD or MUAC <115cm or bilateral oedema) and the presence of: severe oedema, failed appetite test, present with one or more IMCI danger signs, or medical complications<br>(2) severe acute malnutrition (WHZ <-3 SD or MUAC <115cm or bilateral oedema) but without the presence of severe edema, failed appetite test or present with one or more IMCI danger signs<br>(3) moderate acute malnutrition: (WHZ -2~-3 SD and without oedema)<br>(4) not acutely malnourished	Explain care plan:<br>(1) admit for inpatient treatment if child has severe acute malnutrition (WHZ <-3 SD or MUAC <115cm or bilateral oedema) and the presence of: severe oedema, failed appetite test, present with one or more IMCI danger signs, or medical complications<br>(2)refer to outpatient care if child has severe acute malnutrition (WHZ <-3 SD or MUAC <115cm or bilateral oedema) but without the presence of severe edema, failed appetite test or present with one or more IMCI danger signs<br>(3)refer to community MAM treatment if child moderate acute malnutrition: (WHZ -2~-3 ZD and without oedema)	(1) referred to appropriate level of care		[37-40]
Vitamin A supplementation in children aged 6-59 months	(1) clean scissors	(1) 200 000 IU in red gelatin capsules<br>(2) 100 000 IU in blue gelatin capsules		(1) training in Vitamin A provision<br>(2) available guidelines for Vitamin A		(1) assess previous Vitamin A supplementation history		(1) supplement administration:<br>for infants 6-11 months of age - one dose of 100 000 IU of Vitamin A (=30mg	(1) documentation of supplement provision	(1) caregivers are welcomed by staff<br>(2) caregiver questions are answered	[41, 42]

Intervention	Service readiness					Provision of care				Experience of care	References
	**Equipment and supplies (includes equipment and infection prevention items)**	**Medicines and commodities**	**Diagnostics**	**HR/Trained staff and guidelines**	**Basic amenities**	**Nutrition assessment (Nutrition focused physical measurement and examination; Laboratory and medical assessment; Client and dietary history)**	**Medical and nutrition diagnosis**	**Nutrition intervention (Food and supplement (or care) provision; Nutrition counselling)**	**Nutrition monitoring and evaluation (Monitoring and record tracking; Follow-up visit)**		
				supplementation in children				RE)<br>for children 12-59 months 200 000 IU Vitamin A (=60mg RE) every 4-6months; in the form of oral liquid.<br><br>Provide, explain, or counsel on:<br>(2) purpose of Vitamin A supplement<br>(3) dietary diversification.<br>(4) potential side-effects		(3) child's name is requested<br>(4) child is not forced to take supplement<br>(5) child is not given supplement when crying<br>(6) ensure child is comfortable<br>(7) caregiver and child are thanks for the attendance	
Inpatient treatment of complicated SAM	(1) regular thermometer<br>(2) nasogastric tubes<br>(3) sterile IV infusion set<br>(4) disposable gloves<br>(5) finger pricks or lancets<br>(6) cotton balls<br>(7) disinfectant<br>(8) alcohol-based hand rub<br>(9) handwashing soaps<br>(10) low reading thermometer<br>(11) warm blankets<br>(12) heater or lamp or hot water bottle<br>(13) feeding pump<br>(14) respiratory monitor<br>(15) patient chart record<br>(16) single use standard disposable syringes with needles or<br>(17) auto-disable syringes with needles<br>(18) feeding utensils (cup, spoon, dropper, or syringe)<br>(19) Electronic weighing scale (precision of 0.1kg and up to 150kg, and allows tared weighing)<br>(20) sharps container<br>(21) hazardous waste receptacle<br>(22) other waste receptacle<br>(23) band-aids or gauze	(1) sugar, or 10% glucose or sucrose solution<br>(2) F-75 formula<br>(3) antibiotics (Co-trimoxazole, Ampicillin, Gentamicin, Chloramphenicol)<br>(4) Rehydration Solution for Malnutrition (ReSoMal) or ORS<br>(5) Vitamin A supplement (50000 IU; 100000 IU; 200000IU)<br>(6) Multivitamin supplement<br>(7) folic acid 1mg/d doses<br>(8) Electrolyte/mineral/vitamin mix<br>(9) Zinc 2mg/kg/d dose<br>(10) Copper 0.3mg/kg/d doses	(1) glucometer<br>(2) glucometer test strips (within expiration date)<br>(3) Electrolyte blood test (Na, K, Mg)<br>(4) onsite blood test analysis for blood chemistry	(1) training in SAM treatment and management<br>(2) available guidelines on inpatient treatment of complicated SAM	(1) clean environment<br>(2) place for child to rest and lie down	(1) assess shock; lethargic or unconsciousness; cold hands; slow or wear capillary refill, rapid pulse and low blood pressure<br>(2) assess signs of dehydration: skin turgor, sunken eyes, very slow skin pinch<br>(3)assess for severe palmar pallor<br>(4)assess for bilateral pitting oedema<br>(5) assess for eye signs of Vitamin A deficiency: dry conjunctiva or cornea, Bitot spots, corneal ulcerations, keratomalacia<br>(6) assess for localized signs of infection: ear, throat, skin, pneumonia<br>(7) asses axillary temperature (fever)<br>(8) assess rectal temperature (hypothermia)<br>(9) assess for mouth ulcers<br>(10) assess blood glucose<br>(11) assess blood chemistry electrolytes:	(1) diagnosis of hypoglycemia<br>(2) diagnosis of hypothermia<br>(3) diagnosis of dehydration<br>(4) diagnosis of hypokalemia, hyponatremia and hypomagnesia<br>(5) diagnosis of other medical complications and comorbidities (skin lesions in kwashiorkor, meningitis, parasitic infection, contact with measles, TB, HIV etc)<br>(6) presence of eye signs of Vitamin A deficiency	Provision of:<br>(1) stabilization of blood glucose: if child is conscious feed with 50mL of 10% glucose or sucrose solution orally or by nasogastric tube; if child is unconscious treat with IV 10% glucose at 5ml/kg or nasogastric tube, or one teaspoon of sugar sublingually<br>(2) treatment of hypothermia: cover child's head, cover with warmed blankets and place a heater or lamp nearby, or carry out skin-to-skin with caregiver<br>(3) rehydrate using ReSoMal fluid orally or by nasogastric tube or give half strength ORS<br>(4) correct electrolyte imbalance using potassium 3-4mmol/kg and magnesium 0.4-0.6 mmol/kg per day if not already in the	(1) patient charting<br>(2) monitor blood glucose<br>(3) monitor temperature<br>(4) monitor level of consciousness<br>(5) monitor pulse rate<br>(6) monitor respiratory rate<br>(7) monitor urinary frequency<br>(8) monitor stool/vomit frequency<br>(9) monitor amounts offered from therapeutic feeding and amounts left over<br>(10) daily body weight monitoring<br>(11) weekly weight gain calculation<br>(12) monitor signs of rehydration including: moist in eyes, mouth, tears<br>(13) advise for regular follow-up check-ups<br>(14) ensure booster immunizations are given<br>(15) Ensure Vitamin A supplement	(1) ensure tender loving care<br>(2) ensure a cheerful, stimulating environment	[21, 38, 40, 43]

Intervention	Service readiness					Provision of care				Experience of care	References
	Equipment and supplies (includes equipment and infection prevention items)	Medicines and commodities	Diagnostics	HR/Trained staff and guidelines	Basic amenities	Nutrition assessment (Nutrition focused physical measurement and examination; Laboratory and medical assessment; Client and dietary history)	Medical and nutrition diagnosis	Nutrition intervention (Food and supplement (or care) provision; Nutrition counselling)	Nutrition monitoring and evaluation (Monitoring and record tracking; Follow-up visit)		
		(10) Iron 3mg/kg/d dose<br>(11) F100 formula<br>(12) Ringer's lactate solution with 5% dextrose<br>(13) 0.45% saline +5% dextrose solution				K, Na, Mg<br>(12) assess recent intake of food and fluids<br>(13) assess usual diet prior to current illness<br>(14) assess breastfeeding practices<br>(15) assess duration and frequency of diarrhoea and vomiting<br>(16) assess type of diarrhoea<br>(17) assess loss of appetite<br>(18) assess cough >2 weeks<br>(19) assess for other medical complications and comorbidities (skin lesions in kwashiorkor, meningitis, parasitic infection, malaria, measles, TB, HIV etc)		feed.<br>(5) provide routine broad spectrum antibiotics for treatment of infection<br>(6) if there if presence of eye signs Vitamin A deficiency: provision of Vitamin A supplement (for age <6 mo 50000 IU ;age 6-12mo 100000 IU; 13 mo older 200000IU)<br>(7) start feeding with F-75 Milk based formula fed at 100kcal/kg/d rate, based on recommended feeding schedule and progression of 10ml (stabilization phase)<br>(8) F-100 milk based formula and progress to reach up to 150-220 kcal/kg/d (rehabilitation phase)<br>(9) correct micronutrient deficiencies using: Multivitamin supplement; folic acid 1mg/d doses; Electrolyte/mineral/vitamin mix; Zinc 2mg/kg/d dose; Copper 0.3mg/kg/d doses; Iron 3mg/kg/d dose<br>(10) provide structured play therapy 15-30min/day<br>(11) treatment of other complications and comorbidities<br>(12) encourage breastfeeding<br>(13) encourage maternal involvement	received every 6 months<br>(16) transition to outpatient care when medical complications including oedema are resolved, have good appetite and are clinically well and alert.		

Intervention	Service readiness					Provision of care				Experience of care	References
	Equipment and supplies (includes equipment and infection prevention items)	Medicines and commodities	Diagnostics	HR/Trained staff and guidelines	Basic amenities	Nutrition assessment (Nutrition focused physical measurement and examination; Laboratory and medical assessment; Client and dietary history)	Medical and nutrition diagnosis	Nutrition intervention (Food and supplement (or care) provision; Nutrition counselling)	Nutrition monitoring and evaluation (Monitoring and record tracking; Follow-up visit)		
								(14) provide knowledge to parent or carer on feeding: frequent feeding with energy-nutrient- dense foods, and encourage structured play therapy			
Treatment of non-complicated SAM	(1) electronic weighing scale (precision of 0.1kg and up to 150kg, and allows tared weighing)<br>(2) MUAC tape	(1) ready-to-use therapeutic foods<br>(2) ready-to-use supplementary foods<br>(3) medium-quantity lipid-based nutrient supplements		(1) training in non-complicated SAM treatment and feeding<br>(2) available guidelines on treatment of non-complicated SAM<br>(3) training in proper anthropometric measurement		(1) assess weight<br>(2) assess height<br>(3) assess MUAC		(1) provision of food supplement in the form of RUTF, or supplementary<br><br>Provide, explain, or counsel on:<br>(2) emotional and physical stimulation<br>(3) to continue care at home	(1) monitor WHZ or MUAC (depending on initial measure used during screening)<br>(2) discharge from treatment program if weight for heigh length >=-2 SD or MUAC >125mm and have had no oedema for at least two weeks<br>(3) schedule for community follow-up periodically to avoid relapse.		[38]
Counselling on feeding for diarrhea	(1) behavior change materials/counselling materials			(1) training in child feeding during diarrhea<br>(2) available guidelines on counselling on feeding for diarrhea		1) ask about current breast feeding and child feeding practices during diarrhea		Provide, explain, or counsel on:<br>(1) continuation of usual healthy feeding practices (or increased breastfeeding) during diarrhea episodes<br>(2) knowledge on suitable available home fluids immediately upon onset of diarrhea<br>(3) infants who are given formula should be fed at least every three hours if possibly by cup. And avoid special commercial formulas advertised for diarrhoea<br>(4) offer child food every 3-4 hours (6 times a day), frequent small			[44-46]

Intervention	Service readiness					Provision of care				Experience of care	References
	Equipment and supplies (includes equipment and infection prevention items)	Medicines and commodities	Diagnostics	HR/Trained staff and guidelines	Basic amenities	Nutrition assessment (Nutrition focused physical measurement and examination; Laboratory and medical assessment; Client and dietary history)	Medical and nutrition diagnosis	Nutrition intervention (Food and supplement (or care) provision; Nutrition counselling)	Nutrition monitoring and evaluation (Monitoring and record tracking; Follow-up visit)		
								feedings<br>(5) choose energy-rich foods<br>(6) for infants under 6months, continue and increase exclusive breastfeeding<br>(7) children with persistent diarrhea should be given supplementary multivitamin and minerals			
Oral rehydration solution (ORS) during diarrhea	(1) feeding utensils (spoon, cup, syringes)<br>(2) behavior change materials/counselling materials<br>(3) electronic weighing scale (precision of 0.1kg and up to 150kg, and allows tared weighing)<br>(4) guidelines for treating children and adults with some dehydration<br>(5) nasogastric tubes<br>(6) measuring cup with 100mL precision	(1) Oral Rehydration Solution: glucose: 75 mEq sodium: 75 mEq or mmol/L chloride: 65 mEq or mmol/L potassium: 20 mEq or mmol/L citrate: 10 mmol/L osmolarity: 245 mOsm/L glucose: 13.5 g/L sodium chloride: 2.6 g/L potassium chloride: 1.5 g/L trisodium citrate dihydrate*: 2.9 g/L (trisodium citrate dihydrate may be replaced by sodium hydrogen carbonate (sodium bicarbonate) 2.5 g/L. However, as the stability of this latter formulation is very poor under tropical conditions, it is		(1) training in diagnosis and treatment of diarrhea in children<br>(2) training in proper use of ORS treatment and preparation<br>(3) training in counselling<br>(4) available guidelines on the proper use of ORS during diarrhea		(1) assess general condition (i.e. alertness, restless, irritable, lethargy)<br>(2) assess eyes (normal or sunken)<br>(3) assess ability to tolerate fluids<br>(4) assess skin turgor<br>(5) assess presence of blood in stool<br>(6) assess duration of diarrhoea<br>(7) assess number of watery stools per day<br>(8) assess number of episodes of vomiting<br>(9) assess presence of fever, cough or other important problems<br>(10) type and amount of fluids and food taken during illness<br>(11) drugs or other remedies taken<br>(12) immunization history	(1) diagnose degree of dehydration	(1) provide ORS orally at facility if child shows signs of some dehydration or severe dehydration while setting up IV fluids<br>(2) give ORS solution by nasogastric tube if dehydration does not improve through oral administration<br>(3) give two 1-litre packets of new ORS for home-use<br><br>Provide, explain, or counsel on:<br>(4) treat with home therapy if child has no signs of dehydration, and provide ORS at home with other salt containing fluids<br>(5) education on home treatment and preparation of ORS<br>(6) knowledge on other suitable fluids to prevent dehydration at home<br>(7) knowledge on amount of fluids to provide<br>(8) knowledge on signs of dehydration<br>(9) advice to bring child to health worker when	(1) monitor tolerance to rehydration using ORS solution and sigs of dehydrations.<br>(2) reassess child status after 4 hours<br>(3) return to facility for follow-up		[44-46]

Intervention	Service readiness				Provision of care				Experience of care	References
	Equipment and supplies (includes equipment and infection prevention items)	Medicines and commodities	Diagnostics	HR/Trained staff and guidelines	Basic amenities	Nutrition assessment (Nutrition focused physical measurement and examination; Laboratory and medical assessment; Client and dietary history)	Medical and nutrition diagnosis	Nutrition intervention (Food and supplement (or care) provision; Nutrition counselling)	Nutrition monitoring and evaluation (Monitoring and record tracking; Follow-up visit)		
		recommended only when manufactured for immediate use.)						presenting with signs of dehydrations			
Zinc treatment for diarrhea	(1) behavior change materials/counselling materials<br>(2) electronic weighing scale (precision of 0.1kg and up to 150kg, and allows tared weighing)	(1) zinc (10-20mg)		(1) training in diagnosis and treatment of diarrhea in children<br>(2) training in us of Zinc for diarrhea treatment<br>(3) training in counselling<br>(4) available guidelines on zinc treatment for diarrhea		(1) general condition (i.e. alertness, restless, irritable, lethargy)<br>(2) eyes normal or sunken<br>(3) ability to tolerate fluids<br>(4) skin turgor<br>(5) assess muscle wasting (marasmus, oedema, wasted)<br>(6) assess weight<br><br>(7) presence of blood in stool<br>(8) duration of diarrhoea<br>(9) number of watery stools per day<br>(10) number of episodes of vomiting<br>(11) presence of fever, cough or other important problems<br>(12) type and amount of fluids and food taken during illness<br>(13) drugs or other remedies taken<br>(14) immunization history	(1) diagnose degree of dehydration	(1) Provision/ prescription of Zinc as syrup or tablets, and should be given as soon as diarrhoea starts and continued for 10 to 14 days for home treatment<br>(2) provision/ prescription of zinc as soon as the child is able to eat following initial four-hour rehydration period for children with some dehydration<br><br>Provide, explain, or counsel on:<br>(3) Advise and encourage adherence on continued use of zinc for 10-14 days post intervention even if diarrhoea has ended.	(1) identifying correct treatment plan		[44-46]
Iron supplementation in young children aged 2-12 years of age	(1) disposable gloves<br>(2) finger pricks or lancets<br>(3) cotton balls<br>(4) disinfectant<br>(5) alcohol-based hand rub<br>(6) handwashing soaps<br>(7) single use standard disposable syringes with needles or<br>(8) auto-disable syringes with needles<br>(9) sharps container<br>(10) hazardous waste receptacle<br>(11) other waste receptacle<br>(12) band-aids or gauze<br>(13) hot air oven/boiling mechanism/autoclave (when disposable is not available/appropriate)	(1) daily iron: 30mg of elemental iron for children 2-5 (150mg of ferrous sulfate heptahydrate, 90mg of ferrous fumarate or 250mg of ferrous gluconate.<br>(2) intermittent iron: 25 mg elemental iron (drops/ syrups)<br>(3) malaria	(1) Malaria rapid diagnostic test kit<br>(2) onsite malaria testing using microscopy	(1) training in nutrition counselling<br>(2) training in iron supplement provision<br>(3) training in malaria diagnosis using RDT or microscopy<br>(4) training in malaria treatment and dosing<br>(5) training in the counselling for malaria prevention at home<br>(6) available		(1) malaria testing	(1) diagnosing malaria	Provision/ prescription of (for children 1-5 years old):<br>(1) daily supplement of 30mg elemental iron in the form of drops/syrups/tablets for three consecutive months in a year<br>(2) intermittent supplementation of 25 mg of element iron 3 months on and 3 months off and if feasible should be given through the school or calendar	(1) documentation of supplement provision<br>(2) schedule return to facility for follow-up		[47-50]

Intervention	Service readiness					Provision of care				Experience of care	References
	Equipment and supplies (includes equipment and infection prevention items)	Medicines and commodities	Diagnostics	HR/Trained staff and guidelines	Basic amenities	Nutrition assessment (Nutrition focused physical measurement and examination; Laboratory and medical assessment; Client and dietary history)	Medical and nutrition diagnosis	Nutrition intervention (Food and supplement (or care) provision; Nutrition counselling)	Nutrition monitoring and evaluation (Monitoring and record tracking; Follow-up visit)		
	(14) insecticide treated bednets<br>(15) behavior change materials/counselling materials	treatment: Artemisinin-based combination therapies (ACT)		guidelines on iron supplementation in children aged 2-12 years<br>(7) available guidelines on malaria prevention, testing, and diagnosis				year.<br>(3) provision of ACT for malaria treatment if child diagnosed with malaria<br>(4) providing insecticide-treated bednets<br><br>Provide, explain, or counsel on:<br>(5) Instruction on correct dosing of iron supplementation<br>(6) address barriers to adherence to iron supplementation<br>(7) ways to improve acceptability of supplementation to children and caregiver<br>(8) the use of insecticide-treated bed nets at home<br>(9) the importance and purpose of return visits for periodic doses			
Multiple micronutrient supplementation in different forms for children 2-12 years of age	(1) behavior change materials/counselling materials	(1) Multiple Micronutrient supplements in tablet form<br>(2) Multiple Micronutrient Powders: Composition of iron 10-12.5 mg of elemental iron for children aged 2-4 years; Vitamin A: 300 microgram retinol. Zinc 5mg elemental zinc. With or without other micronutrients to achieve 100% RNI.		(1) training in nutrition counselling<br>(2) training in supplement provision and demonstration of proper use<br>(3) available guidelines on MMS in children 2-12 years				(1) provision of multiple micronutrient powder with point-of-use administration: 90 sachets/doses<br><br>Provide, explain, or counsel on:<br>(2) consider the number of sachets that are provided to the caregiver each time, in order to promote adherence and proper use)<br>(3) correct use of sachets<br>(4) proper and hygienic preparation<br>(5) assess baseline caregiver awareness	(1) documentation of supplement provision		[51]

Intervention	Service readiness					Provision of care				Experience of care	References
	**Equipment and supplies (includes equipment and infection prevention items)**	**Medicines and commodities**	**Diagnostics**	**HR/Trained staff and guidelines**	**Basic amenities**	**Nutrition assessment (Nutrition focused physical measurement and examination; Laboratory and medical assessment; Client and dietary history)**	**Medical and nutrition diagnosis**	**Nutrition intervention (Food and supplement (or care) provision; Nutrition counselling)**	**Nutrition monitoring and evaluation (Monitoring and record tracking; Follow-up visit)**		
								knowledge or MNP (6) address misconceptions and misuse			

[1] World Health Organization, "WHO recommendations on antenatal care for a positive pregnancy experience," World Health Organization, Geneva, Switzerland, 9789241549912, 2016 2016.
[2] E. M. DeMaeyer, "Preventing and Controlling Iron Deficiency Anaemia Through Primary Health Care," World Health Organization, Geneva, Switzerland, 1989. [Online]. Available: https://apps.who.int/iris/bitstream/handle/10665/39849/9241542497_eng.pdf?sequence=1&isAllowed=y
[3] S. E. Burger and J. N. Pierre-Louis, "How to Assess Iron Deficiency Anemia and use the Hemocue?," Helen Keller International, 2002 2002. [Online]. Available: https://pdf.usaid.gov/pdf_docs/PNACW824.pdf
[4] COPACK GMBH. "Haemoglobin Colour Scale – How it works." https://haemoglobincolourscale.com/hcs-how.html (accessed March, 2020).
[5] G.-M. Hinnouho *et al.*, "Comparison of haemoglobin assessments by HemoCue and two automated haematology analysers in young Laotian children," (in eng), *Journal of Clinical Pathology,* vol. 71, no. 6, pp. 532-538, 2018/06// 2018, doi: 10.1136/jclinpath-2017-204786.
[6] World Health Organization, "Diagnostic Criteria and Classification of Hyperglycaemia First Detected in Pregnancy," World Health Organization, Geneva, Switzerland, 2011 2011. [Online]. Available: https://apps.who.int/iris/bitstream/handle/10665/85975/WHO_NMH_MND_13.2_eng.pdf?sequence=1
[7] Centers for Disease Control and Prevention, "National Health and Nutrition Examination Survey (NHANES): Oral Glucose Tolerance Test (OGTT) Procedures Manual," Centers for Disease Control and Prevention, Atlanta, GA, 2007 2007.
[8] M. O. Omotayo, K. L. Dickin, K. O. O'Brien, L. M. Neufeld, L. M. De Regil, and R. J. Stoltzfus, "Calcium Supplementation to Prevent Preeclampsia: Translating Guidelines into Practice in Low-Income Countries," (in en), *Advances in Nutrition,* vol. 7, no. 2, pp. 275-278, 2016/03/01/ 2016, doi: 10.3945/an.115.010736.
[9] Cornell University, "Calcium and Iron Folic Acid Supplementation for Pregnant Women: A Trainer's Guide for Healthcare Providers and Community Health Workers," 2015. [Online]. Available: https://cpb-us-e1.wpmucdn.com/blogs.cornell.edu/dist/2/5237/files/2015/01/MICa-Cornell-Ca-IFA-training-materials-21xauo3.pdf
[10] Maternal Health Division Ministry of Health & Family Welfare Government of Indonesia, "National Guidelines for Calcium Supplemenation During Pregnancy and Lactation," Ministry of Health and Family Welfare Government of Indonesia, New Delhi, 2014 2014. [Online]. Available: http://www.nrhmhp.gov.in/sites/default/files/files/NG_calcium.pdf
[11] Nutrition International, "Iron-Folic Acid Supplementation in Pregnancy: Program Logic Model," Nutrition International, 2019. [Online]. Available: https://www.nutritionintl.org/content/user_files/2019/11/NI-IFA-Program-logic-model-and-considerations.pdf
[12] World Health Organization, "Developing and validating an iron and folic acid supplementation indicator for tracking progress towards global nutrition monitoring framework targets," World Health Organization, Geneva, Switzerland, 2018 2018.
[13] J. P. Peña-Rosas, L. M. De-Regil, T. Dowswell, and F. E. Viteri, "Intermittent oral iron supplementation during pregnancy," (in en), *Cochrane Database of Systematic Reviews,* no. 7, 2012 2012, doi: 10.1002/14651858.CD009997.
[14] M. W. Bourassa *et al.*, "Review of the evidence regarding the use of antenatal multiple micronutrient supplementation in low- and middle-income countries," (in en), *Annals of the New York Academy of Sciences,* vol. 1444, no. 1, pp. 6-21, 2019 2019, doi: 10.1111/nyas.14121.
[15] M. Shekar, J. Kakietek, J. Dayton Eberwein, and D. Walters, "An Investment Framework for Nutrition: Reaching the Global Targets for Stunting, Anemia, Breastfeeding and Wasting," The World Bank Group, Washington DC, 2017 2017. Accessed: 2020/08/28/21:08:34. [Online]. Available: https://www.worldbank.org/en/topic/nutrition/publication/an-investment-framework-for-nutrition-reaching-the-global-targets-for-stunting-anemia-breastfeeding-wasting
[16] K. G. Dewey *et al.*, "Effectiveness of a Lipid-Based Nutrient Supplement Intervention in Bangladesh: Pregnancy and Birth Outcomes," FHI 360/Food and Nutrition Technical Assistance III Project (FANTA), Washington, DC, 2016.
[17] World Health Organization. "Vitamin A supplementation." http://www.who.int/elena/titles/full_recommendations/vitamina_supp/en/ (accessed February, 2020).
[18] African Strategies for Health, "Strengthening IPTp Service Delivery Through Facility-Initiated Supervision," Management Sciences for Health, Arlington, VA, 2016. [Online]. Available: https://www.msh.org/sites/default/files/iptp_tool.pdf
[19] World Health Organization, "Guideline: Preventive chemotherapy to control soil-transmitted helminth infections in at-risk population groups," World Health Organization, Geneva, Switzerland, 978-92-4-155011-6, 2017 2017.
[20] World Health Organization, "Guideline: Delayed umbilical cord clamping for improved maternal and infant health and nutrition outcomes," World Health Organization, Geneva, Switzerland, 2014 2014.
[21] World Health Organization, "Pocket book of hospital care for children: guidelines for the management of common childhood illnesses," World Health Organization, Geneva, Switzerland, 978-92-4-154837-3, 2013 2013.
[22] E. Ng and A. D. Loewy, "Position Statement: Guidelines for vitamin K prophylaxis in newborns," *Canadian Family Physician,* vol. 64, no. 10, pp. 736-739, 2018/10// 2018. [Online]. Available: https://www.ncbi.nlm.nih.gov/pmc/articles/PMC6184976/.
[23] Committee on Fetus and Newborn, "Controversies Concerning Vitamin K and the Newborn," (in en), *Pediatrics,* vol. 112, no. 1, pp. 191-192, 2003/07/01/ 2003, doi: 10.1542/peds.112.1.191.
[24] World Health Organization, "WHO recommendations on interventions to improve preterm birth outcomes," World Health Organization, Geneva, Switzerland, 2015 2015.
[25] Maternal and Child Health Integrated Program, "Kangaroo Mother Care: Implementation Guide," Save the Children and The White Ribbon Alliance, Washington, DC, 2012 2012. [Online]. Available: https://www.mchip.net/sites/default/files/MCHIP%20KMC%20Guide_English.pdf
[26] World Health Organization, "Kangaroo mother care: A practical guide," World Health Organization, Geneva, Switzerland, 2003 2003.
[27] World Health Organization, "Recommendations for management of common childhood conditions: newborn conditions, dysentery, pneumonia, oxygen use and delivery, common causes of fever, severe acute malnutrition and supportive care," World Health Organization, Geneva, Switzerland, 978-92-4-150282-5, 2012 2012. Accessed: 2020/08/31/14:01:29. [Online]. Available: http://whqlibdoc.who.int/publications/2012/9789241502825_eng.pdf
[28] World Health Organization, "WHO recommendations on intrapartum care for a positive childbirth experience," World Health Organization, Geneva, Switzerland, 978-92-4-155021-5, 2018 2018.
[29] World Health Organization and The United Nations Children's Fund, "Protecting, promoting and supporting breastfeeding in facilities providing maternity and newborn services: the revised Baby-friendly Hospital Initiative," World Health Organization, Geneva, Switzerland, 2018.
[30] World Health Organization, "Guideline: counselling of women to improve breastfeeding practices," World Health Organization, Geneva, Switzerland, 2018, vol. Licence: CC BY-NC-SA 3.0 IGO.
[31] World Health Organization, "Global Strategy for Infant and Young Child Feeding," World Health Organization, Geneva, Switzerland, 2003 2003.
[32] Pan American Health Organization, "Guiding principles for complementary feeding of the breastfed child," Pan American Health Organization, Washington DC, 2002.
[33] World Health Organization, "Integrated Management of Childhood Illness: Chart Booklet," World Health Organization, Geneva, Switzerland, 2014 2014.
[34] Vitamin Angels, "Reference Manual for Administration of Deworming to Preschool-Age Children in Vitamin A Distribution Projects," Vitamin Angels, 2019 2019.
[35] A. Ashworth, R. Shrimpton, and K. Jamil, "Growth monitoring and promotion: review of evidence of impact," (in en), *Maternal & Child Nutrition,* vol. 4, no. s1, pp. 86-117, 2008 2008, doi: 10.1111/j.1740-8709.2007.00125.x.
[36] N. Mangasaryan, M. Arabi, and W. Schultink, "Revisiting the Concept of Growth Monitoring and its Possible Role in Community-Based Nutrition Programs," *Food and Nutrition Bulletin,* vol. 32, no. 1, pp. 42-53, 2011/03/01/ 2011, doi: 10.1177/156482651103200105.
[37] World Health Organization, "Training Course on Child Growth Assessment," World Health Organization, Geneva, Switzerland, 2008 2008.
[38] World Health Organization and The United Nations Children's Fund, "WHO child growth standards and the identification of severe acute malnutrition in infants and children," World Health Organization, Geneva, Switzerland, 2009 2009.
[39] World Health Organization, "WHO child growth standards: length/height-for-age, weight-for-age, weight-for-length, weight-forheight and body mass index-for-age : methods and development," World Health Organization, Geneva, Switzerland, 2006 2006. [Online]. Available: https://www.who.int/childgrowth/standards/Technical_report.pdf
[40] World Health Organization, "Guideline: Update on the management of severe acute malnutrition in infants and children," World Health Organization, Geneva, Switzerland, 978-92-4-150203-0 978-92-4-150641-0 978-92-4-150179-8 978-92-4-150180-4 978-92-4-150176-7 978-92-4-150178-1 978-92-4-150632-8 978-92-4-150820-9, 2013 2013.
[41] World Health Organization, "Guideline: Vitamin A supplementation in infants and children 6-59 months of age," World Health Organization, Geneva, Switzerland, 2011 2011.
[42] Vitamin Angels, "How to Give Vitamin A to Children 6-59 Months," Vitamin Angels, 2017.
[43] World Health Organization, "Guidelines for the inpatient treatment of severely malnourished children," World Health Organization, Geneva, Switzerland, 978-92-4-154609-6, 2003.
[44] World Health Organization, "Implementing the new recommendations on the clinical management of diarrhoea : guidelines for policy makers and programme managers," World Health Organization, Geneva, Switzerland, 2006 2006.
[45] World Health Organization, "The treatment of diarrhoea: a manual for physicians and other senior health workers," World Health Organization, Geneva, Switzerland, 978-92-4-159318-2, 2005 2005.
[46] The United Nations Children's Fund and World Health Organization, "Diarrhoea: why children are still dying and what can be done," United Nations Children's Fund, New York, 978-92-806-4462-3 978-92-4-159841-5, 2009 2009.
[47] World Health Organization, "Guideline: Daily iron supplements in infants and children," World Health Organization, Geneva, Switzerland, 978-92-4-154952-3, 2016 2016. Accessed: 2020/08/31/14:57:47. [Online]. Available: http://www.ncbi.nlm.nih.gov/books/NBK362032/
[48] World Health Organization, "Guideline: Intermittent iron supplementation in preschool and school-age children," World Health Organization, Geneva, Switzerland, 978-92-4-150200-9, 2011 2011. Accessed: 2020/08/31/14:59:28. [Online]. Available: http://whqlibdoc.who.int/publications/2011/9789241502009_eng.pdf
[49] World Health Organization, "Guidelines for the treatment of malaria," World Health Organization, Geneva, Switzerland, 978-92-4-154912-7, 2015 2015.
[50] World Health Organization, "Universal access to malaria diagnostic testing: an operational manual," World Health Organization, Geneva, Switzerland, 978-92-4-150209-2, 2011 2011.
[51] World Health Organization, "WHO guideline: Use of multiple micronutrient powders for point-of-use fortification of foods consumed by infants and young children aged 6-23 months and children aged 2-12 years," World Health Organization, Geneva, Switzerland, 978-92-4-154994-3, 2016 2016.

Supplementary Table 3: Tabulation of item matching from intervention guidelines to SPA/SARA items

Interventions	Exact match	High partial	Low partial	Non-match	Total items	Percent exact match	Percent non-match
**Pregnancy**	55	45	48	56	204	27%	27%
Assessment of anemia during pregnancy	22	7	8	12	49	45%	24%
Blood glucose testing during pregnancy	13	2	3	8	26	50%	31%
Calcium supplementation during pregnancy	2	2	5	7	16	13%	44%
Daily IFA during pregnancy	3	5	5	1	14	21%	7%
Deworming in pregnant women	2	3	2	3	10	20%	30%
Intermittent IFA during pregnancy	3	4	5	2	14	21%	14%
IPT during pregnancy	6	1	2	1	10	60%	10%
Maternal balanced energy and protein supplementation	3	3	4	8	18	17%	44%
MMS during pregnancy	0	2	4	6	12	0%	50%
Nutrition education and counselling during pregnancy	3	11	6	3	23	13%	13%
Vitamin A supplementation during pregnancy	0	5	4	5	14	0%	36%
**Infant/Newborn**	16	6	5	57	84	19%	68%
Delayed cord clamping	2	1	1	8	12	17%	67%
Kangaroo mother care	6	2	1	34	43	14%	79%
Skin-to-skin contact	1	1	0	3	5	20%	60%
Vitamin K administration	7	2	3	12	24	29%	50%
**Child**	131	72	71	201	475	28%	42%
Assessment and treatment of anemia in children	17	4	7	13	41	41%	32%
Complementary feeding counselling	4	2	6	3	15	27%	20%
Counselling on feeding for diarrhea	2	5	1	3	11	18%	27%
Deworming in young children aged 12-59 months	3	3	3	7	16	19%	44%
Growth monitoring	10	3	2	4	19	53%	21%
Inpatient treatment of complicated SAM	17	10	2	72	101	17%	71%
Preventative iron supplementation in children aged 6-23 months	17	6	7	9	39	44%	23%
Preventative iron supplementation in young children aged 2-12 years of age	17	7	7	11	42	40%	26%
Multiple Micronutrient Powder for children aged 6-23 months	0	0	4	8	12	0%	67%
Multiple micronutrient supplementation in different forms for children 2-12 years of age	0	0	4	9	13	0%	69%
Oral rehydration solution (ORS) during diarrhea	10	8	3	15	36	28%	42%
Postnatal breastfeeding counselling (for early and exclusive breastfeeding, and PMTCT)	3	4	2	13	22	14%	59%
Screening of acute malnutrition	21	5	2	13	41	51%	32%
SQ-LNS for children	0	0	6	3	9	0%	33%
Treatment of non-complicated SAM	1	3	2	9	15	7%	60%
Vitamin A supplementation in children aged 6-59 months	2	4	9	2	17	12%	12%
Zinc treatment for diarrhea	7	8	4	7	26	27%	27%
**Grand Total**	**204**	**123**	**124**	**314**	**765**	**27%**	**41%**

Supplementary material: Operationalization of nutrition QoC indices

The proposed indices included a few items that currently are not collected within SPA/SARA (e.g., calcium supplements and ready-to-use therapeutic foods (RUTFs)). As such, if an analysis is being conducted using secondary SPA/SARA data, several items should be removed from the indices. **Table 2** presents information on data availability for each item based on a review of the core SPA and SARA questionnaires. If ***SPA*** data is utilized to generate the nutrition quality of care indices for ***pregnant women*** two readiness items (MUAC tape, calcium supplements) must be excluded as they are not collected in the SPA questionnaire. In addition, one provision of care item (asked about a client's last menstrual period) should be omitted as this item is only used for the delivery of calcium supplements, an intervention not captured by the SPA questionnaire. Removing these items from the QoC indices for pregnant women reduces the total number of interventions represented from eight to seven as calcium supplementation is completely omitted. If ***SPA*** data is utilized to generate the nutrition quality of care indices for ***children under five***, two readiness items (MUAC tape, ready-to-use therapeutic foods) and one provision of care item (prescribed or provided RUTF or supplementary foods) must be excluded as they are not collected in the SPA. Removing these items from the QoC indices for children under 5 reduces the total number of interventions represented from eleven to ten as treatment of non-complicated SAM is completely omitted. If ***SARA*** data is utilized to generate the nutrition quality of care indices, only the service readiness indices can be calculated as the SARA survey does include direct observations or exit interviews to assess provision or experience of care items. Furthermore, the service readiness indices will be limited as many of the items in the proposed nutrition QoC indices relating to basic amenities and staff training are not included the in the SARA survey. Additionally, the country specific context and national clinical care guidelines should be consulted when determining items to include in the indices. For example, in non-malaria endemic areas, IPTp items should be removed from the QoC indices for pregnant women.
