# Supplementary material for: Advancing nutrition measurement: Developing quantitative measures of nutrition service quality for pregnant women and children in low‐ and middle‐income country health systems
Source: Matern Child Nutr. 2021 Nov 3;18(1):e13279. doi: 10.1111/mcn.13279 (PMC8710116; doi:10.1111/mcn.13279)
Supplement: Supplementary file 2 — Data S1. Supporting Information [file MCN-18-e13279-s001.pdf]

JOHNS HOPKINS
BLOOMBERG SCHOOL
of PUBLIC HEALTH

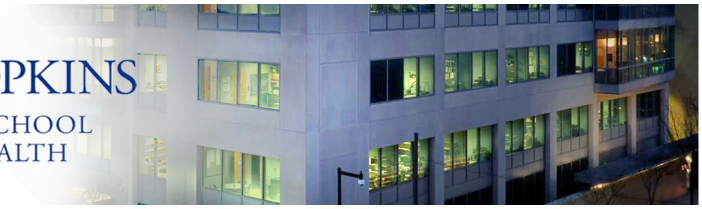

Information

We invite you to participate in a brief online expert survey from the Data for Decisions to Extend Nutrition Transformation (DataDENT) and Improving Measurement and Program Design (Improve) project teams at the Johns Hopkins Bloomberg School of Public Health. (*Funding source: The Bill & Melinda Gates Foundation*) You have been identified as an expert in nutrition with experience working in low- and middle-income countries (LMICs) and as such we would greatly appreciate your contribution to this work.

The survey will take approximately 20-30 minutes to complete and the survey portal will be open until July 15th, 2020.

The survey is part of ongoing efforts to develop indicators for quality-adjusted coverage of nutrition interventions delivered through health systems. While there are many definitions and frameworks for defining quality of care, there is a lack of consensus on summary measures of the quality of nutrition services for pregnant women and children. The goal of this survey is to elicit expert opinion on the items that are important for the provision of high quality nutrition services and to include in measures of health system nutrition service quality. Only nutrition specific interventions delivered at the health care facility are considered for inclusion in the survey.

The survey findings will be used to develop quality of care metrics that can be derived from existing health facility assessment data (e.g. Service Provision Assessment, Service Availability and Readiness Assessment) and may also be used for recommendations to improve health facility surveys. The findings will be used in analyses that we expect will be disseminated in peer-reviewed publications.

If you have any questions, please reach out to Shannon King (sking50@jhu.edu). Many thanks in advance for your participation before the closing date of July 15th, 2020

Do you agree to participate?

By clicking "Yes" I agree to voluntarily participate in the survey. I understand the purpose of the survey. I know that I can stop taking the survey at any point without consequences. I understand that any personal identifying information provided within the survey will remain confidential.

- ○ Yes
- ○ No

QoC Explanation & Expertise

Survey background

In the survey we will ask you to select and rank “items” required for delivery and measurement of high-quality nutrition services through health systems.

The nutrition interventions and items presented were identified through a review of global policy documents (e.g. Essential Nutrition Actions) & standardized health facility questionnaires (e.g. SPA, SARA).

Items are organized according to three dimensions of the WHO Quality of Care Framework for Maternal and Newborn Health: 1) facility readiness, 2) provision of care, and 3) experience of care.

Dimension Description:

Facility readiness refers to the capability of health facilities to provide a service of minimum acceptable standards. It is measured by the availability of both physical resources and human resources. Physical resources include items related to the availability of an appropriate physical environment, and required equipment, supplies, medicines or commodities, and diagnostics. Human resources include items related to the availability of competent, motivated providers including trained staff and clinical guidelines.

Provision of care refers to the quality of delivery of interventions by providers to clients (i.e. the content of care). This includes following evidence-based practices for routine care and management of complications, actionable information systems and functional referral systems. Provision of care is assessed by observing consultations and recording what occurs.

Experience of care refers to the client's experience including effective communication by the care provider about the services provided, client expectations, and client rights; care provided with respect and preservation of dignity; and client access to emotional and social support of their choice.

The survey assumes respondents have knowledge of protocols and HR/material requirements for high-quality delivery of nutrition interventions through LMIC health systems. For which of the following sub-populations do you feel qualified to answer questions about intervention requirements?

(Note: You will only be asked about interventions related to the sub-populations you select below; you may tick both boxes if you have expertise in both areas.)

☐ Pregnant women ☐ Children under 5

Pregnancy readiness

Section 1: Nutrition interventions delivered during pregnancy

Interventions considered to be part of this package include:

- Assessment and treatment of anemia
- Daily or intermittent iron folic acid (IFA) supplementation
- Multiple micronutrient supplementation
- Nutrition education and counselling (including breastfeeding counselling)
- Maternal balanced energy and protein supplementation
- Vitamin A supplementation
- Calcium supplementation

- Blood glucose testing
- Deworming
- Intermittent preventive treatment for malaria in pregnant women (IPTp)

Only nutrition specific interventions delivered at health facilities are considered for inclusion in the survey.

For each quality of care dimension (Facility readiness, Provision of care, Experience of care), a set of items has been identified related to delivery of the package of nutrition services for pregnant women listed above.

We are asking you to identify the importance of each item for **delivery** of a high-quality package of nutrition services for pregnant women.

For each item, please categorize the item by importance based on your expert opinion. If you are not familiar with an item, please place it in the box marked "Don't know".

For the purpose of delivering a high-quality package of nutrition services for pregnant women, the item can be ranked as …

- **Essential**
- **Very important**
- **Somewhat important**
- **Unimportant/Non-informative**
- **Don't know**

Items will appear in a column on the left-hand side with boxes for each rank category on the right-hand side. For each item, simply click the item and drag it into the box of your choice.

Section 1A: Facility readiness

Facility readiness refers to the capability of health facilities to provide a service of

minimum acceptable standards. It is measured by the availability of both physical resources and human resources. Physical resources include items related to the availability of an appropriate physical environment, and required equipment, supplies, medicines or commodities, and diagnostics. Human resources include items related to the availability of competent, motivated providers including trained staff and clinical guidelines.

Please categorize the following **equipment** items based on importance for the provision of a package of high quality nutrition services for pregnant women.

The facility has the following items available...

Items

Adult weighing scale

Alcohol-based hand rub

Auto-disable syringes with needles; single use standard disposable syringes with needles

Disinfectant (environmental)

Disposable gloves

Handwashing soap

Hot air oven/boiling mechanism/autoclave

MUAC tape

Sharps container

Stadiometer or height rod

Waste receptacle (pedal bin) with lid and plastic bin liner

Other, non-hazardous waste receptacle

Visual aids for education

Essential

Very important

Somewhat important

Unimportant

Don't know

Please categorize the following **medicines & commodities** items based on importance for the provision of a package of high quality nutrition services for pregnant women.

The facility has the following items available on-site...

Items

Albendazole/Mebendazole

Balanced Energy and Protein supplements

Calcium supplements

Folic acid tablet (either stand-alone or in combination with iron)

Iron tablets

MMS formulation: UNIMAPP formula

Sulfadoxine-pyrimethamine (SP) for IPTp

Vitamin A capsules

Essential

Very important

Somewhat important

Unimportant

Don't know

Please categorize the following **diagnostic tests** based on importance for the provision of a package of high quality nutrition services for pregnant women.

The facility has the ability to test on-site for…

Items

Blood glucose levels

Hemoglobin levels

Essential

Very important

Somewhat important

Unimportant

Don't know

Please categorize the following **guideline** items based on importance for the provision of high quality nutrition services for pregnant women.

The facility has the following items available...

Items

Guidelines for antenatal care (ANC)

Guidelines for intermittent preventive treatment of malaria during pregnancy

Guidelines for infant and young child feeding (IYCF)

Essential

Very important

Somewhat important

Unimportant

Don't know

Please categorize the following **staff training** items based on importance for the provision of high quality nutrition services for pregnant women.

The facility has at least one staff member trained in the last two years in…

Items

Antenatal care (broad ANC training)

ANC screening (e.g., blood pressure, urine glucose, and protein)

Complications of pregnancy and their management

Counseling for ANC (e.g., nutrition, FP and newborn care)

Intermittent preventive treatment of malaria in pregnancy

Infant and young child feeding

Nutritional assessment of the pregnant woman, such as Body Mass Index calculation and Mid-Upper Arm Circumference measurement

Standard precautions for safe blood collection

Essential

Very important

Somewhat important

Unimportant

Don't know

Please categorize the following **basic amenities** based on importance for the provision of a package of high quality nutrition services for pregnant women.

The facility has the following amenities...

Items	
Place for women to sit/lie down	Essential
Power	
Improved water source	
Room with auditory and visual privacy	
Sanitation facilities	Very important
Communication equipment	
Computer with email/internet	
Emergency transportation	
Clean environment	
	Somewhat important

Unimportant

Don't know

In previous questions we have listed items currently assessed in the standard SPA/SARA facility survey questionnaires. Are any other physical resources items needed for **delivery** of a package of high quality nutrition services for pregnant women that you suggest to add?

- Yes
- No

Please list the items and provide a brief justification for each.

The following is a list of items that you categorized as "essential" in service readiness for the delivery of high quality nutrition services for pregnant women. Of these items, we are interested to know which are **the most critical** that could be used as signals of readiness. Please rank these items relative to one another in the order of their importance for inclusion in a **summary measure** of nutrition service readiness for pregnant women (1 being the most important item).

Items will appear in a single list, simply click the item and drag the item to reorder. Once one item has been reordered, numbers will appear beside each item that designate the rank.

Adult weighing scale

Alcohol-based hand rub

Auto-disable syringes with needles; single use standard disposable syringes with needles

Disinfectant (environmental)

Disposable gloves

Handwashing soaps

Hot air oven/boiling mechanism/autoclave (when disposable is not available/appropriate)

MUAC tape

Sharps container

Stadiometer or height rod

Waste receptacle (pedal bin) with lid and plastic bin liner

Other waste receptacle

Visual aids for education

Albendazole/Mebendazole

Balanced Energy and Protein supplements

Calcium supplements

Folic acid tablet (either stand-alone or in combination with iron)

Iron tablets

MMS formulation: UNIMAPP formula

Sulfadoxine-pyrimethamine (SP) for IPTp

Vitamin A capsules

Diagnostics test for blood glucose levels

Diagnostics test for hemoglobin levels

Guidelines for antenatal care (ANC)

Guidelines for intermittent preventive treatment for malaria during pregnancy

Guidelines for infant and young child feeding (IYCF)

Staff trained in Antenatal care (broad ANC training)

Staff trained in ANC screening (e.g., blood pressure, urine glucose, and protein)

Staff trained in Complications of pregnancy and their management

Staff trained in Counseling for ANC (e.g., nutrition, FP and newborn care)

Staff trained in Intermittent preventive treatment of malaria in pregnancy

Staff trained in Infant and young child feeding

Staff trained in Nutritional assessment of the pregnant woman, such as Body Mass Index calculation and Mid-Upper Arm Circumference measurement

Staff trained in Standard precautions for safe blood collection

Place for women to sit/lie down

Power

Improved water source

Room with auditory and visual privacy

Sanitation facilities

Communication equipment

Computer with email/internet

Emergency transportation

Clean environment

Pregnancy Provision of Care

Section 1B: Provision of care

Provision of care refers to the quality of delivery of interventions by providers to clients (i.e. the content of care). This includes following evidence-based practices for routine care and management of complications, actionable information systems and functional referral systems. Provision of care is assessed by observing consultations and recording what occurs.

Please categorize the following **assessment** items based on importance for the provision of a package of high quality nutrition services for pregnant women.

During ANC visit the provider....

Items

Asked about, performed or referred the client for hemoglobin testing

Asked about when the client's last menstrual period began

Inspected conjunctiva or examined the client for pallor

Essential

Very important

Somewhat important

Unimportant

Don't know

Please categorize the following **intervention** items based on importance for the provision of a package of high quality nutrition services for pregnant women.

During ANC visit the provider ...

Items

Essential

Explained the purpose of iron or folic acid

Advised on potential side effects of IFA

Explained how to take iron or folic acid pills

Provided or prescribed iron pills or folic acid pills or both

Explained the purpose of deworming

Provided albendazole or mebendazole

Explained the purpose of preventative treatment with anti-malaria medicines

Explained potential side effects of IPTp-SP

Provided or prescribed preventive treatment: IPTp-SP

Discussed nutrition (i.e., quantity or quality of food to eat) during the pregnancy

Discussed early initiation and prolonged breastfeeding

Discussed exclusive breastfeeding

Very important

Somewhat important

Unimportant

Don't know

Please categorize the following **documentation** items based on importance for the provision of a package of high quality nutrition services for pregnant women.

During ANC visit the provider ...

Items	Essential	Very important	Somewhat important	Unimportant
Documented IFA supplement provision				
Documented provision of deworming medication				
Documented calcium supplement provision				
Documented balanced energy protein supplement provision				
Documented IPTp-SP provision				
Documented iron supplement provision/prescription				
Documented multiple micronutrient supplement provision				
Documented Vitamin A supplement provision				

Don't know

In previous questions we have listed items currently assessed in the standard SPA/SARA facility survey questionnaires. Are any other provision of care items needed for **delivery** of a package of high quality nutrition services for pregnant women that you suggest to add?

- Yes
- No

Please list the items and provide a brief justification for each.

The following is a list of items that you categorized as "essential" in provision of care for delivery of high quality nutrition services for pregnant women. Of these items, we are interested to know which are **the most critical** that could be used as signals of technical service quality. Please rank these items relative to one another in the order of their importance for inclusion in a **summary measure** of nutrition service quality for pregnant women (1 being the most important item).

Items will appear in a single list, simply click the item and drag the item to reorder. Once one item has been reordered, numbers will appear beside each item that designate the

rank.

Asked about, performed or referred the client for hemoglobin testing

Asked about when the client’s last menstrual period began

Inspected conjunctiva or examined the client for pallor

Explained the purpose of iron or folic acid

Advised on potential side effects of IFA

Explained how to take iron or folic acid pills

Provided or prescribed iron pills or folic acid pills or both

Explained the purpose of deworming

Provided albendazole or mebendazole

Explained the purpose of preventative treatment with anti-malaria medicines

Explained potential side effects of IPTp-SP

Provided or prescribed preventive treatment: IPTp-SP

Discussed nutrition (i.e., quantity or quality of food to eat) during the pregnancy

Discussed early initiation and prolonged breastfeeding

Discussed exclusive breastfeeding

Documented IFA supplement provision

Documented provision of deworming medication

Documented calcium supplement provision

Documented balanced energy protein supplement provision

Documented IPTp-SP provision

Documented iron supplement provision/prescription

Documented multiple micronutrient supplement provision

Documented Vitamin A supplement provision

Pregnancy experience of Care

Section 1C:
Experience of care refers to the client's experience including effective communication by the care provider about the services provided, client expectations, and client rights; care provided with respect and preservation of dignity; and client access to emotional and social support of their choice.

Please categorize the following **experience of care** items based on importance for the provision of a package of high quality nutrition services for pregnant women.

Items

- Client is able to discuss problems or concerns about pregnancy with provider
- Client satisfied with the amount of explanation received about the problem or treatment
- Client satisfied with how the staff treated them
- Client has privacy from having others see the consultation
- Client has privacy from having others hear the consultation
- Client satisfied with the wait time
- Client satisfied with the number of days services are available at the facility
- Client satisfied with the hours of service at the facility
- Client satisfied with the cost for services or

Essential

Very important

Somewhat important

treatments

Client satisfied with the availability of medicines at the facility

Client satisfied with the cleanliness of the facility

Unimportant

Don't know

In previous questions we have listed items currently assessed in the standard SPA/SARA facility survey questionnaires. Are any other experience of care items needed for **delivery** of a package of high quality nutrition services for pregnant women that you suggest to add?

- Yes
- No

Please list the items and provide a brief justification for each.

The following is a list of items that you categorized as “essential” in experience of care for delivery of high quality nutrition services for pregnant women. Of these items, we are interested to know which are **the most critical** that could be used as

signals of interpersonal service quality. Please rank these items relative to one another in the order of their importance for inclusion in a **summary measure** of nutrition service quality for pregnant women (1 being the most important item).

Items will appear in a single list, simply click the item and drag the item to reorder. Once one item has been reordered, numbers will appear beside each item that designate the rank.

Client is able to discuss problems or concerns about pregnancy with provider

Client satisfied with the amount of explanation received about the problem or treatment

Client satisfied with how the staff treated them

Client has privacy from having others see the consultation

Client has privacy from having others hear the consultation

Client satisfied with the wait time

Client satisfied with the number of days services are available at the facility

Client satisfied with the hours of service at the facility

Client satisfied with the cost for services or treatments

Client satisfied with the availability of medicines at the facility

Client satisfied with the cleanliness of the facility

The interventions delivered to pregnant women that we have included in our assessment are: assessment and treatment of anemia, blood glucose testing, calcium supplementation, daily or intermittent iron folic acid (IFA) supplementation, deworming, intermittent preventive treatment for malaria in pregnant women (IPTp), maternal balanced energy and protein supplementation, multiple micronutrient supplementation, nutrition education and counselling (including breastfeeding counselling), and vitamin A supplementation.

Are there any other nutrition interventions delivered through the health system during antenatal care that should be included?

- ○ Yes
- ○ No

Please list the interventions and provide a brief justification for each.

Child readiness

Section 2: Nutrition interventions delivered to children under 5

Interventions included within this section include:

- Growth monitoring and promotion
- Complementary feeding counselling
- Postnatal breastfeeding counselling (for early and exclusive breastfeeding and PMTCT)
- Deworming
- Assessment and treatment of anemia
- Iron supplementation
- Screening for acute malnutrition
- Treatment of non-complicated severe acute malnutrition
- Inpatient treatment of complicated severe acute malnutrition
- SQ-LNS
- Multiple micronutrient powder for complementary feeding

- Multiple micronutrient supplementation tablets
- Vitamin A supplementation
- Oral rehydration solution (ORS) during diarrhea
- Zinc treatment for diarrhea
- Counselling on feeding for diarrhea

Only nutrition specific interventions delivered at health facilities are considered for inclusion in the survey.

For each quality of care dimension (Facility readiness, Provision of care, Experience of care), a set of items has been identified related to delivery of the package of nutrition services for children listed above.

We are asking you to identify the importance of each item for **delivery** of a high-quality package of nutrition services for children.

For each item, please categorize the item by importance based on your expert opinion. If you are not familiar with an item, please place it in the box marked "Don't know".

For the purpose of delivering a high-quality package of nutrition services for children, the item can be ranked as …

- **Essential**
- **Very important**
- **Somewhat important**
- **Unimportant/Non-informative**
- **Don't know**

Items will appear in a column on the left-hand side with boxes for each rank category on the right-hand side. For each item, simply click the item and drag it into the box of your choice.

Section 2A: Facility readiness

Facility readiness refers to the capability of health facilities to provide a service of minimum acceptable standards. It is measured by the availability of both physical resources and human resources. Physical resources include items related to the availability of an appropriate physical environment, and required equipment, supplies, medicines or commodities, and diagnostics. Human resources include items related to the availability of competent, motivated providers including trained staff and clinical guidelines.

Please categorize the following **equipment** items based on importance for the provision of a package of high quality nutrition services for children.

The facility has the following items available...

Items

Alcohol-based hand rub

Auto-disable syringes with needles or single use standard disposable syringes with needles

Blank/unused individual child vaccination cards or booklets

Cup and spoon

Disinfectant (environmental)

Disposable gloves

Growth charts

Handwashing soap

Height or length board

Hot air oven/boiling mechanism/autoclave

Infant weighing scale (100 gram graduation)

Essential

Very important

Somewhat important

Intravenous infusion kit

Nasogastric tube

Regular thermometer

Sharps container

Tape for measuring circumference

Waste receptacle (pedal bin) with lid and plastic bin liner

Other, non-hazardous waste receptacle

Visual aids for teaching care givers

Unimportant

Don't know

Please categorize the following **medicines & commodities** items based on importance for the provision of a package of high quality nutrition services for children.

The facility has the following items available on-site...

Items

Albendazole/Mebendazole

Artemisinin-based combination therapy

F-75 formula

Iron tablets

Multiple micronutrient powders

Multiple micronutrient supplements in tablet form for children

Essential

Very important

Oral rehydration salts (ORS) sachets

Ready-to-use therapeutic foods

Small quantity-lipid nutrition supplement (SQ-LNS)

Vitamin A capsules

Zinc tablets

Ampicillin/gentamycin injection

Ringers lactate

Normal saline (5% dextrose)

Folic acid tablet (stand-alone only)

Insecticide treated bednets (LLITN)

Somewhat important

Unimportant

Don't know

Please categorize the following **diagnostic tests** based on importance for the provision of a package of high quality nutrition services for children.

The facility has the ability to test on-site for...

Items

Malaria (RDT or microscopy)

Essential

Blood chemistry (serum creatinine and liver function tests)

Blood glucose levels

Hemoglobin levels

Very important

Somewhat important

Unimportant

Don't know

Please categorize the following **guideline** items based on importance for the provision of high quality nutrition services for children.

The facility has the following items available...

Items

Guidelines for growth monitoring in children

Guidelines for malaria prevention, testing and diagnosis

Guidelines for Integrated Management of Childhood Illness (IMCI)

Guidelines for infant and young child feeding counseling

Essential

Very important

Somewhat important

Unimportant

Don't know

Please categorize the following **staff training** items based on importance for the provision of high quality nutrition services for children.

The facility has at least one staff member trained in the last two years in…

Items

Integrated Management of Childhood Illness (IMCI)

Case management/treatment of malaria in children

Breastfeeding

Complementary feeding in children

Nutrition counseling for newborn of mother with HIV/AIDS

Diagnosis and/or treatment of diarrhea

Micronutrient deficiencies and/or nutritional assessment

Malaria diagnosis using RDT or microscopy

Malaria treatment and dosing

Essential

Very important

Somewhat important

Unimportant

Don't know

Please categorize the following **basic amenities** based on importance for the provision of a package of high quality nutrition services for children.

The facility has the following amenities...

Items

Power

Improved water source

Room with auditory and visual privacy

Sanitation facilities

Communication equipment

Computer with email/internet

Emergency transportation

Clean environment

Essential

Very important

Somewhat important

Unimportant

Don't know

In previous questions we have listed items currently assessed in the standard SPA/SARA facility survey questionnaires. Are any other physical resources items needed for **delivery** of a package of high quality nutrition services for children that you suggest to add?

- Yes
- No

Please list the items and provide a brief justification for each.

The following is a list of items that you categorized as “essential” in service readiness for delivery of high quality nutrition services for children. Of these items, we are interested to know which are **the most critical** that could be used as signals of readiness. Please rank these items relative to one another in the order of their importance for inclusion in a **summary measure** of nutrition service readiness for children (1 being the most important item).

Items will appear in a single list, simply click the item and drag the item to reorder. Once one item has been reordered, numbers will appear beside each item that designate the rank.

Alcohol-based hand rub

Auto-disable syringes with needles or single use standard disposable syringes with needles

Blank/unused individual child vaccination cards or booklets

Cup and spoon

Disinfectant (environmental)

Disposable gloves

Growth charts

Handwashing soap

Height or length board

Hot air oven/boiling mechanism/autoclave

Infant weighing scale (100 gram graduation)

Intravenous infusion kit

Nasogastric tube

Regular thermometer

Sharps container

Tape for measuring circumference

Waste receptacle (pedal bin) with lid and plastic bin liner

Other, non-hazardous waste receptacle

Visual aids for teaching care givers

Albendazole/Mebendazole

Artemisinin-based combination therapy

F-75 formula

Iron tablets

Multiple micronutrient powders

Multiple micronutrient supplements in tablet form for children

Oral rehydration salts (ORS) sachets

Ready-to-use therapeutic foods

Small quantity-lipid nutrition supplement (SQ-LNS)

Vitamin A capsules

Zinc tablets

Ampicillin/gentamycin injection

Ringers lactate

Normal saline (5% dextrose)

Folic acid tablet (stand-alone only)

Insecticide treated bednets (LLITN)

Diagnostic test for malaria (RDT or microscopy)

Diagnostic test for blood chemistry (serum creatinine and liver function tests)

Diagnostic test for blood glucose levels

Diagnostic test for hemoglobin levels

Guidelines for growth monitoring in children

Guidelines for malaria prevention, testing and diagnosis

Guidelines for Integrated Management of Childhood Illness (IMCI)

Guidelines for infant and young child feeding counseling

Staff trained in Integrated Management of Childhood Illness (IMCI)

Staff trained in Case management/treatment of malaria in children

Staff trained in Breastfeeding

Staff trained in Complementary feeding in children

Staff trained in Nutrition counseling for newborn of mother with HIV/AIDS

Staff trained in Diagnosis and/or treatment of diarrhea

Staff trained in Micronutrient deficiencies and/or nutritional assessment

Staff trained in Malaria diagnosis using RDT or microscopy

Staff trained in Malaria treatment and dosing

Power

Improved water source

Room with auditory and visual privacy

Sanitation facilities

Communication equipment

Computer with email/internet

Emergency transportation

Clean environment

Child provision of care

Section 2B: Provision of care

Provision of care refers to the quality of delivery of interventions by providers to clients (i.e. the content of care). This includes following evidence-based practices for routine care and management of complications, actionable information systems and functional referral systems. Provision of care is assessed by observing consultations and recording what occurs.

Please categorize the following **assessment** items based on importance for the provision of a package of high quality nutrition services for children.

During sick child visit the provider....

Items

- Assessed previous deworming history
- Asked about feeding or breastfeeding habits or practices for child during illness
- Asked about normal breastfeeding feeding habits or practices when the child is not ill
- Asked about normal feeding habits or practices when the child is not ill
- Assessed previous Vitamin A supplementation history
- Weighed the child
- Pressed both feet to check for edema
- Took child's temperature by thermometer
- Felt the child for fever or body hotness

Essential

Very important

Somewhat important

Counted respiration (breaths) for 60 seconds

Auscultated child (listen to chest with stethoscope) or count pulse

Checked skin turgor for dehydration (e.g. pinch abdominal skin)

Looked into child's mouth

Checked for neck stiffness

Looked in child's ear

Felt behind child's ear

Checked for enlarged lymph nodes in 2 or more of the following sites: neck, axillae, groin

Assessed palm pallor

Looked at the child’s immunization card or asked caretaker about child vaccination history

Offered the child something to drink or asked the mother to put the child to the breast

Plotted child weight on growth curve

Asked if the child is unable to drink or breastfeed

Asked if the child has vomited

Asked if the child has diarrhea

Asked if the child has convulsions

Asked if the child has a fever

Unimportant

Don't know

Asked if the child vomits everything

Asked about presence of cough, or difficulties breathing (e.g. fast breathing or chest-in drawing)

Please categorize the following **diagnosis** items based on importance for the provision of a package of high quality nutrition services for children.

During sick child visit the provider ...

Items

Provided malaria testing results

Essential

Very important

Somewhat important

Unimportant

Don't know

Please categorize the following **intervention** items based on importance for the provision of a package of high quality nutrition services for children.

During sick child visit the provider …

Items

Mentioned the child's weight or growth to the caretaker, or discussed growth chart

Provided general information about feeding or breastfeeding the child even when not sick

Provided education on home treatment and preparation of ORS

Prescribed home ORT

Prescribed or provided initial ORT in facility

Prescribed or provided extra feeding liquids

Essential

Very important

Prescribed or provided RUTF or supplementary foods

Prescribed zinc

Advised and encouraged adherence on continued use of zinc

Told the caretaker to continue feeding the child during this illness

Provided instruction on correct dosing of iron supplementation in children

Provided/prescribed iron supplement

Provided ACT for malaria treatment if child diagnosed with malaria

Provided/prescribed Vitamin A

Administered the correct dose of deworming medication

Discussed follow-up visit for the sick child

Referred the child to appropriate level of care

Prescribed or provided multiple micronutrient powders

Prescribed or provided multiple micronutrient supplements

Prescribed or provided SQ-LNS

Provided instructions on correct supplement dosing and use of SQ-LNS

Somewhat important

Unimportant

Don't know

Please categorize the following **documentation** items based on importance for the provision of a package of high quality nutrition services for children.

During sick child visit the provider ...

Items	Essential	Very important	Somewhat important	Unimportant
Documented deworming medication provision				
Documented iron supplement provision				
Documented Vitamin A supplement provision				
Documented multiple micronutrient powder provision				
Documented multiple micronutrient supplement provision				
Documented SQ-LNS provision				

Don't know

In previous questions we have listed items currently assessed in the standard SPA/SARA facility survey questionnaires. Are any other provision of care items needed for **delivery** of a package of high quality nutrition services for children that you suggest to add?

- Yes
- No

Please list the items and provide a brief justification for each.

The following is a list of items that you categorized as "essential" in provision of care for delivery of high quality nutrition services for children. Of these items, we are interested to know which are **the most critical** that could be used as signals of technical service quality. Please rank these items relative to one another in the order of their importance for inclusion in a **summary measure** of nutrition service quality for children (1 being the most important item).

Items will appear in a single list, simply click the item and drag the item to reorder. Once one item has been reordered, numbers will appear beside each item that designate the rank.

Assessed previous deworming history

Asked about feeding or breastfeeding habits or practices for child during illness

Asked about normal breastfeeding feeding habits or practices when the child is not ill

Asked about normal feeding habits or practices when the child is not ill

Assessed previous Vitamin A supplementation history

Weighed the child

Pressed both feet to check for edema

Took child's temperature by thermometer

Felt the child for fever or body hotness

Counted respiration (breaths) for 60 seconds

Auscultated child (listen to chest with stethoscope) or count pulse

Checked skin turgor for dehydration (e.g. pinch abdominal skin)

Looked into child's mouth

Checked for neck stiffness

Looked in child's ear

Felt behind child's ear

Checked for enlarged lymph nodes in 2 or more of the following sites: neck, axillae, groin

Assessed palm pallor

Looked at the child’s immunization card or asked caretaker about child vaccination history

Offered the child something to drink or asked the mother to put the child to the breast

Plotted child weight on growth curve

Asked if the child is unable to drink or breastfeed

Asked if the child has vomited

Asked if the child has diarrhea

Asked if the child has convulsions

Asked if the child has a fever

Asked if the child vomits everything

Asked about presence of cough, or difficulties breathing (e.g. fast breathing or chest-in drawing)

Provided malaria testing results

Mentioned the child's weight or growth to the caretaker, or discussed growth chart

Provided general information about feeding or breastfeeding the child even when not sick

Provided education on home treatment and preparation of ORS

Prescribed home ORT

Prescribed or provided initial ORT in facility

Prescribed or provided extra feeding liquids

Prescribed or provided RUTF or supplementary foods

Prescribed zinc

Advised and encouraged adherence on continued use of zinc

Told the caretaker to continue feeding the child during this illness

Provided instruction on correct dosing of iron supplementation in children

Provided/prescribed iron supplement

Provided ACT for malaria treatment if child diagnosed with malaria

Provided/prescribed Vitamin A

Administered the correct dose of deworming medication

Discussed follow-up visit for the sick child

Referred the child to appropriate level of care

Prescribed or provided multiple micronutrient powders

Prescribed or provided multiple micronutrient supplements

Prescribed or provided SQ-LNS

Provided instructions on correct supplement dosing and use of SQ-LNS

Documented deworming medication provision

Documented iron supplement provision

Documented Vitamin A supplement provision

Documented multiple micronutrient powder provision

Documented multiple micronutrient supplement provision

Documented SQ-LNS provision

Child Experience of Care

Section 2C:

Experience of care refers to the client's experience including effective communication by the care provider about the services provided, client expectations, and client rights; care provided with respect and preservation of dignity; and client access to emotional and social support of their choice.

Please categorize the following **experience of care** items based on importance for the provision of a package of high quality nutrition services for children.

Items	Essential
Client is able to discuss problems or concerns about child's illness with provider	
Client satisfied with the amount of explanation received about the problem or treatment	

Client satisfied with how the staff treated them

Client has privacy from having others see the consultation

Client has privacy from having others hear the consultation

Client satisfied with the wait time

Client satisfied with the number of days services are available at the facility

Client satisfied with the hours of service at the facility

Client satisfied with the cost for services or treatments

Client satisfied with the availability of medicines at the facility

Client satisfied with the cleanliness of the facility

Very important

Somewhat important

Unimportant

Don't know

In previous questions we have listed items currently assessed in the standard SPA/SARA facility survey questionnaires. Are any other experience of care items needed for **delivery** of a package of high quality nutrition services for children that you suggest to add?

Yes

No

Please list the items and provide a brief justification for each.

The following is a list of items that you categorized as “essential” in experience of care for delivery of high quality nutrition services for children. Of these items, we are interested to know which are **the most critical** that could be used as signals of interpersonal service quality. Please rank these items relative to one another in the order of their importance for inclusion in a **summary measure** of nutrition service quality for children (1 being the most important item).

Items will appear in a single list, simply click the item and drag the item to reorder. Once one item has been reordered, numbers will appear beside each item that designate the rank.

Client is able to discuss problems or concerns about child’s illness with provider

Client satisfied with the amount of explanation received about the problem or treatment

Client satisfied with how the staff treated them

Client has privacy from having others see the consultation

Client has privacy from having others hear the consultation

Client satisfied with the wait time

Client satisfied with the number of days services are available at the facility

Client satisfied with the hours of service at the facility

Client satisfied with the cost for services or treatments

Client satisfied with the availability of medicines at the facility

Client satisfied with the cleanliness of the facility

The interventions delivered to children that we have included in our assessment are: growth monitoring and promotion, complementary feeding counselling, postnatal breastfeeding counselling (for early and exclusive breastfeeding and PMTCT), deworming, assessment and treatment of anemia, iron supplementation, screening for acute malnutrition, treatment of non-complicated severe acute malnutrition, inpatient treatment of complicated severe acute malnutrition, SQ-LNS, multiple micronutrient powder for complementary feeding, multiple micronutrient supplementation tablets, vitamin A supplementation, oral rehydration solution (ORS) during diarrhea, zinc treatment for diarrhea, and counselling on feeding for diarrhea

Are there any other nutrition interventions delivered through the health system for children that should be included?

- Yes
- No

Please list the interventions and provide a brief justification for each.

Respondent Information

Section 3: Respondent Information
Please answer the following questions about yourself and your current role. This information will be used for internal purposes and will not be disseminated.

Name:

Email address

Organization:

Position title:

Country work location (City, country)

Years of experience in nutrition

In this survey we utilized a drag and drop categorization question type instead of a Likert scale question type. To inform future survey efforts, which question type do you prefer?

- ○ Drag and drop categorization
- ○ Likert scale

Powered by Qualtrics
